# Enzymology and Structural Basis of Glycosyltransferases Involved in Saponin C28 Carboxylic Acid *O*‑d‑Fucosylation

**DOI:** 10.1021/jacsau.5c00907

**Published:** 2025-11-24

**Authors:** Graham A. Hudson, Jose H. Pereira, Peter H. Winegar, David M. FitzGerald, Andy DeGiovanni, Xiaoyue Chen, Xixi Zhao, Maria C. T. Astolfi, James Reed, Amr El-Demerdash, Martin Rejzek, Shingo Kikuchi, Anne Osbourn, Henrik V. Scheller, Paul D. Adams, Jay D. Keasling

**Affiliations:** † 124489 Joint BioEnergy Institute, Lawrence Berkeley National Laboratory, Emeryville, California 94608, United States; ‡ Biological Systems and Engineering, Lawrence Berkeley National Laboratory, Berkeley, California 94720, United States; § California Institute for Quantitative Biosciences (QB3 Institute), 1438 University of California, Berkeley, California 94720, United States; ∥ Department of Molecular and Cell Biology, University of California, Berkeley, California 94720, United States; ⊥ Molecular Biophysics and Integrated Bioimaging Division, Lawrence Berkeley National Laboratory, Berkeley, California 94720, United States; # Environmental Genomics and Systems Biology Division, 1666 Lawrence Berkeley National Laboratory, 1 Cyclotron Road, Berkeley, California 94720, United States; ∇ Department of Plant and Microbial Biology, University of California, Berkeley, California 94720, United States; ○ Department of Bioengineering, University of California, Berkeley, California 94720, United States; ◆ John Innes Centre, Norwich Research Park, Norwich NR4 7UH, U.K.; ¶ Department of Chemical and Biomolecular Engineering, University of California, Berkeley, California 94720, United States; ■ The Novo Nordisk Foundation Center for Biosustainability, Technical University Denmark, Kemitorvet, Building 220, 2800 Kongens Lyngby, Denmark

**Keywords:** Saponin biosynthesis, saponin glycosylation, glycodiversification, glycosyltransferase enzymology, fucosyltransferases, GT1, protein structure, X-ray crystallography

## Abstract

Saponins are a class of natural products composed of an oxidized triterpene core adorned with glycosylations, ultimately giving rise to medicinally important compounds bearing bioactivity that includes, but is not limited to, anti-inflammatory, antimicrobial, antifungal, antiarrhythmic, and immunostimulatory activities. QS-21 is a prominent immunostimulatory saponin and is a critical adjuvant component of several FDA-approved vaccines. One linchpin modification in the biosynthesis and bioactivity of several saponins, including QS-21, is *O*-d-fucosylation via an ester linkage. In QS-21, the C28-COOH *O*-d-fucose residue is part of a linear oligosaccharide that is an integral component of the “core pharmacophore” responsible for its immunomodulatory activity. In this work, we performed in-depth in vitro enzymological characterization of two glycosyltransferases involved in C28-COOH *O*-d-fucosylation during the maturation of two saponin natural products: QsFucT from QS-21 biosynthesis and SvFucT from vaccaroside biosynthesis. QsFucT was previously shown to be a UDP-4-keto-6-deoxy-d-glucosyltransferase; our data reveal that the taxonomically distant SvFucT also functions as a UDP-4-keto-6-deoxy-d-glucosyltransferase and that both glycosyltransferases act on a triterpene acceptor with low-micromolar affinity. Substrate scope studies demonstrate that both enzymes are highly permissive with regard to both the triterpene acceptor and, unexpectedly, the UDP-sugar donor. These data also reveal that the conserved C3-OH branched trisaccharide of QS-21 and other saponins may serve an unusual biosynthetic role in protecting the C23 aldehyde from spurious reduction during biosynthesis. In addition, we crystallized and solved the structures of QsFucT and SvFucT, providing the first structural characterization of 4-keto-6-deoxy-d-glucosyltranferases in the glycosyltransferase family 1 (GT1) class of enzymes and used these structures to explore the importance of conserved residues in the active site. These data suggest that both QsFucT and SvFucT could be leveraged to rapidly explore saponin chemical space and glycodiversify these important medicinal compounds through engineered biosynthesis or in vitro enzymatic synthesis, possibly leading to novel analogs with enhanced physicochemical or pharmacological properties.

## Graphical abstract

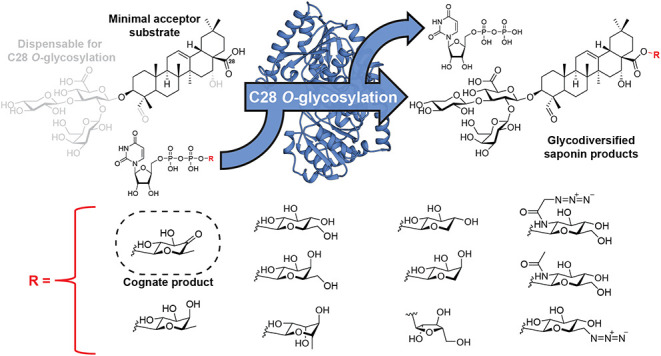

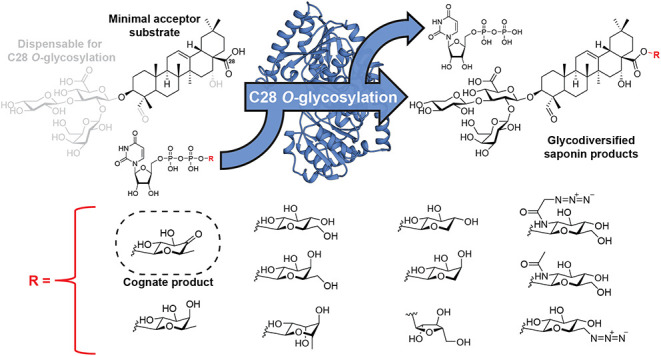

## Introduction

Saponins are a class of natural products (NPs) isolated from producing plants and select marine invertebrates.^1,2^ These NPs are biosynthesized by iteratively and site-specifically oxidizing and glycosylating a triterpene core with possible ancillary modifications such as acylation, giving rise to structurally complex NPs (Figure 1A). Many saponins present attractive bioactivities that lend themselves to possible therapeutic use, including, but not limited to, anti-inflammatories, antimicrobials, antifungals, antiarrhythmic drugs, and immunostimulatory adjuvants.^3–6^ QS-7 and QS-21 are immunomodulatory saponins natively produced by and isolated from *Quillaja saponaria* (the Chilean soapbark tree), with QS-21 being a critical component of several vaccine formulations, either approved or in clinical trials, for the prevention of influenza, malaria, shingles, respiratory syncytial virus (RSV), herpes simplex virus type 2 (HSV-2), Ebola, and COVID-19.^7^ Medicinal chemistry efforts have revealed that, despite its formidable structure, only a modest subset of modifications constitute the “core pharmacophore” necessary and sufficient for QS-21/QS-7’s immunomodulatory bioactivity. Specifically, this “core pharmacophore” includes C16α hydroxylation of the β-amyrin triterpene skeleton; C28-COOH *O*-glycosylation with a linear trisaccharide consisting of d-fucose (d-Fuc), l-rhamnose (l-Rha), and d-xylose (d-Xyl); and C4 *O*-acetylation of d-Fuc (Figure 1A).^8^

**Figure 1:**
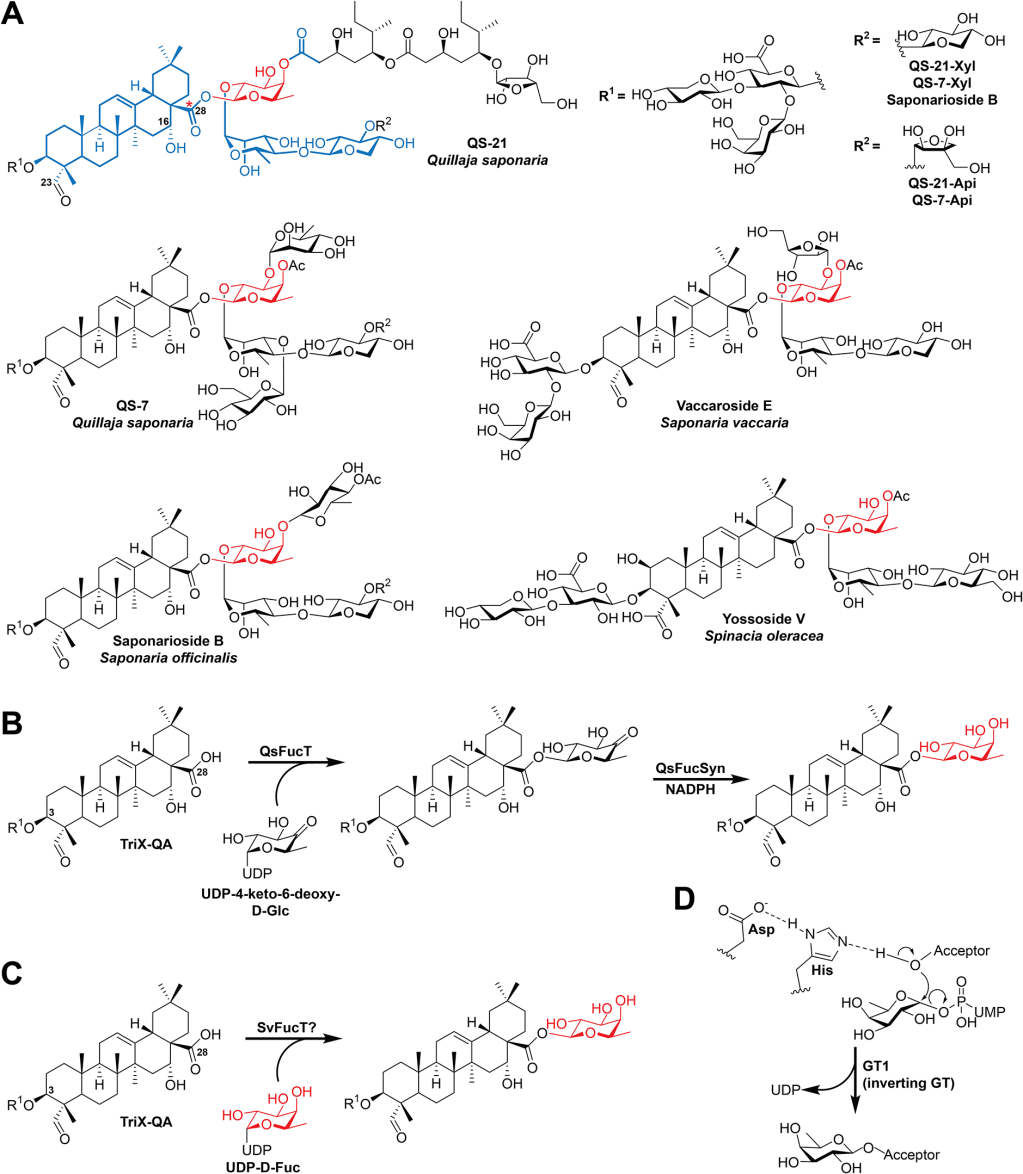
**Saponins featuring C28-COOH *O*-** d-fucosylation. (A) Structures of various saponins that feature C28-COOH *O*-d-fucosylation as a critical biosynthetic step. d-Fuc is emphasized in red, and the “core pharmacophore” of QS-21/QS-7, which also includes d-Fuc, is denoted in blue. The base-labile C28 ester bond, which can be hydrolyzed to yield the quillaic acid core bearing the C3-OH O-linked branched trisaccharide (TriX-QA), is denoted with a red asterisk. (B) C28-COOH *O*-d-fucosylation in the biosynthesis of QS-21/QS-7 proceeds through a two-step process, where 4-keto-6-deoxy-d-Glc is attached by QsFucT prior to reduction by a dedicated reductase, QsFucSyn, to afford d-Fuc.^13^ (C) Possible route of C28-COOH *O*-d-fucosylation in vaccaroside biosynthesis, directly utilizing UDP-d-Fuc. (D) Catalytic mechanism of GT1s, which results in inversion of stereochemistry at the C1 anomeric carbon. UDP, uridine diphosphate; UMP, uridine monophosphate.

Given the integral role that the C28-COOH *O*-linked trisaccharide plays in QS-21/QS-7’s immunostimulatory activity, glycodiversification of these sugars is an attractive avenue to discover novel analogs of QS-21/QS-7 that may have altered pharmacokinetic properties, enhanced bioactivity, or abrogated hemolytic toxicity. Total chemical synthesis has been used to synthesize QS-21 and analogs thereof; however, due to the prevalence of stereocenters and complex/low-yielding chemistry involved in precise installation of the numerous glycosidic bonds, chemical synthesis remains a challenging and relatively low-throughput approach for exploring saponin chemical space. For example, the first total chemical synthesis of QS-21 required 76 steps from quillaic acid and resulted in a vanishingly small overall yield.^9^ Semisynthetic strategies have also been employed to generate analogs of QS-21 by starting with advanced biosynthetic intermediates and installing modifications to alter the identity of the specific sugar residues or the composition/length of the fucose-attached acyl group.^8^ However, in both cases, syntheses rely on complex and often low-efficiency transformations to effectively install glycosidic bonds with intended linkages.^10,11^ This can render glycodiversification infeasible due to the exponential nature of chemical space when attempting to survey all permutations of a glycosidic chain utilizing multiple constituent sugars.

Leveraging biosynthetic enzymes for (chemo)­enzymatic synthesis of saponins and their analogs is a third approach that exploits the exquisite catalytic efficiency and site selectivity imbued onto enzymes through millions of years of evolution. Combined with modern laboratory-assisted evolution techniques guided by machine learning, biosynthetic enzymes can be reprogrammed to efficiently accept alternative substrates while retaining their catalytic efficiency and site selectivity.^12^ However, these investigations greatly benefit from, and often require, basal characterization of the enzyme to know its native substrate scope, kinetic properties, active site architecture, etc. Recently, the full biosynthetic pathways for QS-7 and QS-21 have been elucidated by functional expression in *Nicotiana benthamiana* (tobacco).^13,14^ Contemporary with these efforts has been the characterization of several other saponin pathways beyond QS-21/QS-7 that also feature C28-COOH *O*-d-fucosylation, including yossosides, vaccarosides, and saponariosides (Figure 1A).^15–17^ While the constituent enzymes that give rise to these saponins have been identified, little to no in-depth enzymological or structural biological characterization has been performed on them. Given the crucial role C28-COOH *O*-d-fucosylation plays in the immunomodulatory activity of QS-21/QS-7 and the broader implications of understanding these enzymes across other saponin families, we sought to comprehensively characterize the glycosyltransferases involved in C28-COOH *O*-d-fucosylation from QS-21/QS-7 biosynthesis, QsFucT (UGT74BX1), and from vaccaroside biosynthesis, SvFucT (UGT74CD2).

QsFucT and SvFucT are members of the glycosyltransferase family 1 (GT1) family of GTs. Members of this family feature a conserved His-Asp catalytic dyad hypothesized to deprotonate the sugar acceptor, rendering it more nucleophilic to attack the sugar donor, an activated uridine diphosphate (UDP)-sugar. The nucleophile of the acceptor attacks the anomeric C1 of the sugar opposite of its phosphoester linkage to UDP in an S_N_2 mechanism, resulting in the formation of a new glycosidic bond between the acceptor and sugar donor as well as ejection of UDP (Figure 1D).^18^ This results in inversion of stereochemistry at the sugar’s anomeric C1 position; as such, GT1s are often termed inverting GTs. Of the characterized GT1s, greater than 80% have been shown to be glucosyltransferases, with the remainder being predominantly galactosyl-, rhamnosyl-, xylosyl-, arabinosyl-, and glucuronosyl-transferases. This overrepresentation of glucosyltransferases both in nature and in characterized GT1s intrinsically limits the ability to accurately predict and precisely engineer UDP-sugar substrate preference.^18^ Nevertheless, significant insight has been gleaned regarding the biophysical underpinnings of UDP-sugar recruitment in characterized GT1s. Sugar recognition by GT1s is largely driven by a C-terminal domain that features a highly conserved plant secondary product glycosylation (PSPG) motif which interacts with the UDP moiety as well as with the sugar itself (Figure S2); however, other structural determinants beyond the PSPG have been shown to be pivotal in UDP-sugar recognition, thereby complicating de novo substrate scope prediction and engineering.^19–23^ Comparatively little is known regarding GT1 acceptor recognition, which is mediated by a large and poorly conserved nonpolar pocket situated in the N-terminal domain that is thought to dictate acceptor binding conformation through subtle differences in hydrophobicity and electronic interactions.^18,20,24^ In general, there is a pressing need for in-depth characterization of GT1s to develop robust predictive models for acceptor and donor substrate scope and enable engineering efforts.

Both QsFucT and SvFucT are involved in the installation of an ester-linked C28-COOH *O*-d-Fuc residue during biosynthesis of QS-21/QS-7 and vaccarosides, respectively.^13,15^ Despite being initially suspected to be a fucosyltransferase, QsFucT was unexpectedly shown to utilize UDP-4-keto-6-deoxy-d-glucose, an unprecedented UDP-sugar donor traditionally associated with the biosynthesis of UDP-l-rhamnose, which is subsequently reduced by a dedicated short-chain dehydrogenase/reductase, QsFucSyn, thereby yielding the reduced d-Fuc residue (Figure 1B).^13^ It was also shown that QsFucSyn is incapable of biosynthesizing discrete UDP-d-Fuc from UDP-4-keto-6-deoxy-d-Glc.^13^ This paradigm, where 4-keto-6-deoxy-d-Glc is first attached to an acceptor and reduced by a dedicated reductase, was also shown to extend to the biosynthesis of saponariosides and likely to yossosides as well; in the latter case, intermediates isolated from heterologous expression of the yossoside fucosyltransferase SOAP6 in *N. benthamiana* resulted in the detection of products bearing both a ketodeoxyhexose and d-Fuc.^16,17^ In contrast, transient expression of SvFucT in tobacco resulted in detection of saponins bearing the fully reduced d-Fuc, and it remains unclear whether this pathway also involves an intermediate bearing 4-keto-6-deoxy-d-Glc (Figure 1C). Furthermore, expression of SvNMD, a member of the neomenthol dehydrogenase family, was shown to both biosynthetically produce UDP-d-Fuc and enhance production of fucosylated vaccaroside intermediates.^15^ These data suggest that QsFucT and SvFucT may have “divergent convergent” biosynthetic routes that differ in pathway but nonetheless result in a C28-COOH *O*-fucosylated product; this hypothesis is partially supported by these enzymes being only 42.9% identical and 53.3% similar (Figure S2 and Table S3). For detectable C28-COOH *O*-glycosylation activity, both enzymes required C3-OH *O*-d-glucuronosylation of the quillaic acid aglycone prior to C28-COOH *O*-glycosylation; this requirement was also demonstrated for the medicagenic core of yossosides, which similarly bear an ester-linked C28-COOH *O*-d-Fuc but only on intermediates also bearing a C3-OH *O*-d-glucuronosylation.^13,15,17^ These foundational studies underscore a further need to gain detailed insight into how saponin C28-COOH *O*-d-fucosylation is accomplished and reconcile these seemingly divergent biosynthetic routes as a prerequisite to the biosynthetic engineering of natural and novel C28-COOH *O*-d-fucosylated saponins.

In this work, we performed in-depth enzymological and structural characterization of both QsFucT and SvFucT. To enable these endeavors, we established a novel enzymatic pathway for the scalable synthesis of UDP-d-Fuc, which is not commercially available, directly from UDP-d-Glc. In vitro glycosylation and kinetic assays demonstrated that both QsFucT and SvFucT natively function as UDP-4-keto-6-deoxy-d-glucosyltransferases. We further characterized the substrate scope of both enzymes with regard to triterpene acceptors and UDP-sugar donors, revealing that both enzymes can act on simple oleanane aglycones and exhibit unusually permissive UDP-sugar substrate scopes, greatly enhancing their potential for use in the biosynthetic or enzymatic glycodiversification of saponins. These studies also serendipitously revealed that the C3-OH *O*-linked branched trisaccharide of QS-21/QS-7 likely plays a role in preventing spurious reduction of the C23 aldehyde of the quillaic acid acceptor by QsFucSyn. Lastly, we crystallized and determined the X-ray structures of both enzymes, providing the founding crystallographic structures for GT1 4-keto-6-deoxyglucosyltransferases. Using these data, we explored the importance of conserved residues in both enzymes’ active sites.

## Results and Discussion

### Base Hydrolysis of QS-21 Yields an Ideal Acceptor for Investigating QsFucT and SvFucT

To enable in-depth enzymological characterization of QsFucT and SvFucT, a suitable β-amyrin-based triterpene saponin acceptor bearing, at minimum, C3-OH *O*-glucuronosylation and C28 carboxylic acid was required. While several saponins are commercially available that fit these minimum criteria (calenduloside E [C3-OH *O*-glucuronosylated oleanolic acid] and C3-OH *O*-glucuronosylated quillaic acid), their very poor aqueous solubility (≪1 mg/mL) could result in substrate self-association and/or require the use of cosolvents that may denature enzymes during the course of in vitro assays and confound experimental results. Given that the C28-COOH O-linked saccharide of QS-21/QS-7 is joined to the quillaic acid core by a base-labile ester linkage while the quillaic acid core and C3-OH O-linked trisaccharide are base-stable (Figure 1A), we predicted that commercially available QS-21 could be base-hydrolyzed to afford the biosynthetic intermediate composed of quillaic acid with the full C3-OH *O*-trisaccharide (3-*O*-{β-d-xylopyranosyl-(1→3)-[β-d-galactopyranosyl-(1→2)]-β-d-glucopyranosiduronic acid}-quillaic acid, hereafter “TriX-QA”; Figure 1A–C), thus providing an ideal acceptor for characterization of QsFucT and SvFucT that should be fully water-soluble at concentrations to enable enzymological characterization. Previous endeavors by Gin and co-workers have leveraged base hydrolysis of bulk *Q. saponaria* saponins to provide TriX-QA in scalable quantities pursuant to downstream semisynthetic derivatization.^25^

Several milligrams of commercially sourced QS-21 were dissolved in a solution of excess potassium hydroxide and heated to hydrolyze the ester linkage followed by neutralization and purification using solid-phase extraction. From 5 mg of QS-21, 2.2 mg of TriX-QA was isolated, representing an ∼92% yield. The resulting solid was analyzed by LC-MS and, importantly, was chemically indistinguishable from TriX-QA isolated from heterologous production in *N. benthamiana* (Figure S3).^13^

### Scaled Enzymatic Synthesis of UDP-4-keto-6-deoxy-d-Glc and UDP-d-Fuc

Beyond having a suitable saponin acceptor, in vitro characterization of QsFucT and SvFucT required serviceable amounts (i.e., several milligrams) of possible cognate UDP-sugar donors UDP-4-keto-6-deoxy-d-Glc and UDP-d-Fuc. Based on previous studies, we overexpressed and purified *Acanthocystis turfacea* chlorella virus 1 UDP-glucose-4,6-dehydratase (ATCV-1-UG46DH), which has been shown to robustly convert UDP-d-Glc to UDP-4-keto-6-deoxy-d-Glc.^13,26^ Treatment of UDP-d-Glc with ATCV-1-UG46DH in the presence of NAD^+^ resulted in full conversion of UDP-d-Glc to UDP-4-keto-6-deoxy-d-Glc using only 0.7% catalyst loading (ratio of enzyme to substrate concentrations) in an overnight reaction (Figure S4). Subsequent purification by HPLC resulted in isolation of 5.2 mg of UDP-4-keto-6-deoxy-d-Glc from 7.9 mg of UDP-d-Glc, representing ∼68% yield (Figure S5).

UDP-d-Fuc is not available commercially, and previous investigations have relied on enzymatic synthesis resembling natural sugar salvage pathways in which free d-Fuc sugar is first phosphorylated (using ATP) by an engineered galactose kinase (GalK) to produce d-fucose-1-phosphate (d-Fuc-1-P). Subsequently, galactose-1-phosphate uridyltransferase (GalPUT) transfers UDP from UDP-d-Glc to d-Fuc-1-P, thereby generating UDP-d-Fuc and d-glucose-1-phosphate.^27^ This enzymatic route suffers from the drawback that efficient conversion is dependent on also including regenerative systems for both ATP and UDP-d-Glc, as (super)­stoichiometric quantities of these reactants frustrate the yield due to both enzymatic inhibition and the reverse uridyltransferase reaction.^28^ We hypothesized that an alternative route utilizing the direct biosynthesis of UDP-d-Fuc from UDP-d-Glc was possible. Pursuant to testing this, we overexpressed and purified SvNMD as well as WsbK from *Geobacillus tepidamans* GS5-97^T^; the latter has previously been shown to convert dTDP-4-keto-6-deoxy-d-Glc to dTDP-d-Fuc.^29^ Both enzymes were tested in a one-pot enzymatic synthesis combining ATCV-1-UG46DH (at 0.7% catalyst loading) and SvNMD or WsbK (at 0.1% catalyst loading), and the reaction products were assessed via LC-MS. In both cases, a new product peak with a mass and retention time identical to that of a bona fide UDP-d-Fuc standard was observed; however, only approximately 15% of the UDP-4-keto-6-deoxy-d-Glc intermediate was converted into UDP-d-Fuc by SvNMD, while WsbK achieved 95% conversion (Figure S6). This one-pot enzymatic reaction utilizing WsbK for reduction was scaled, and the produced UDP-d-Fuc was subsequently purified, resulting in isolation of 9.2 mg of UDP-d-Fuc from 13.6 mg of UDP-d-Glc starting material, representing ∼70% yield (Figure S7). The efficiency of WsbK combined with the advantage of mitigating regeneration systems associated with GalK/GalPUT enzymatic synthesis makes this strategy an attractive alternative to produce UDP-d-Fuc for future studies of d-fucosyltransferases.

### In Vitro Glycosylation of TriX-QA by QsFucT and SvFucT with UDP-4-keto-6-deoxy-d-Glc and UDP-d-Fuc

Having serviceable quantities of a TriX-QA, UDP-4-keto-6-deoxy-d-Glc, and UDP-d-Fuc on-hand, we were now poised to investigate whether QsFucT and SvFucT function as fucosyltransferases or are 4-keto-6-deoxyglucosyltransferases (Figure 2A). First, TriX-QA was converted with UDP-4-keto-6-deoxy-d-Glc in the presence of either QsFucT or SvFucT overnight, and the reaction products were analyzed by LC-MS. For both enzymes, the starting material peak was significantly reduced in size, and four new product peaks were observed (Figure 2B,C). The two later-eluting product peaks had masses consistent with TriX-QA bearing 4-keto-6-deoxy-d-Glc, while the remaining early-eluting species had masses corresponding to the *gem*-diol hydrate (Figures 2B,C and S8). These two products, each bearing two separable yet isobaric species, likely reflect isomerization of saponin-attached 4-keto-6-deoxy-d-Glc occurring through an enolate intermediate.^13^ Including QsFucSyn in the glycosylation reaction results in the near complete disappearance of starting material and convergence of all four intermediate product peaks onto a single species with a mass consistent with that of TriX-QA-Fuc, consistent with previous findings for QsFucT (Figures 2 and S8).^13^ The significant increase in product accumulation upon inclusion of QsFucSyn suggests that both enzymes may suffer significant product inhibition by the 4-keto-6-deoxy-d-Glc-bearing intermediate that is relieved upon reduction to the d-Fuc-bearing counterpart. Performing in vitro glycosylation utilizing UDP-d-Fuc as the sugar donor resulted in the appearance of a peak with retention time and mass identical to those produced in the presence of UDP-4-keto-6-deoxy-d-Glc and QsFucSyn with comparable efficiency (Figures 2 and S8). These glycosylation assays were unable to distinguish whether QsFucT and SvFucT were 4-keto-6-deoxy-d-glucosyltransferases or d-fucosyltransferases because the vast majority of starting material were consumed in the presence of both UDP-sugars. To resolve this ambiguity, we conducted a more granular analysis by determining the kinetic parameters of QsFucT and SvFucT for the saponin acceptor and for both UDP-sugars.

**Figure 2:**
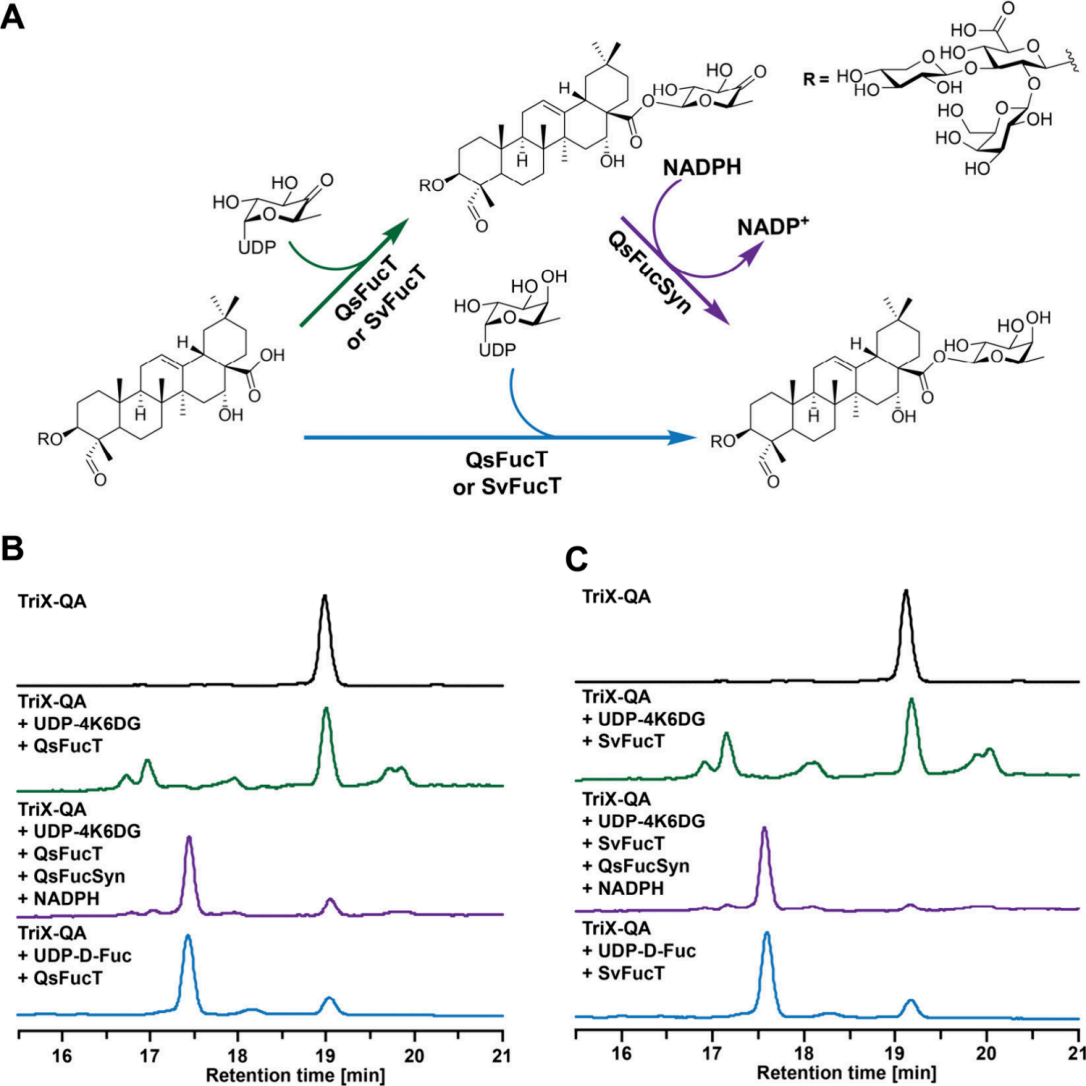
**Glycosylation assays of QsFucT and SvFucT on TriX-QA using UDP-4-keto-6-deoxy-** d-Glc or UDP-d-Fuc. (A) Reaction diagram for glycosylation assays using either UDP-4-keto-6-deoxy-d-Glc, which after attachment to the saponin core undergoes reduction by QsFucSyn to afford d-Fuc, or discrete UDP-d-Fuc, resulting in the fucosylated product.^13^ (B) A220 HPLC chromatograms of QsFucT reactions. (C) A220 HPLC chromatograms of SvFucT reactions. Mass spectra for product peaks can be found in Figure S8. UDP-4K6DG, UDP-4-keto-6-deoxy-d-Glc.

### Kinetic Analysis of QsFucT and SvFucT for TriX-QA, UDP-4-keto-6-deoxy-d-Glc, and UDP-d-Fuc

Given that both QsFucT and SvFucT were able to effectively glycosylate using UDP-4-keto-6-deoxy-d-Glc and UDP-d-Fuc in previous assays, thus preventing any conclusions regarding their substrate scope, we next performed kinetic analyses on both enzymes with regard to TriX-QA and both UDP-sugars. Glycosyltransferases utilizing activated nucleotide sugars eject a stoichiometric equivalent of the corresponding nucleotide diphosphate for each product formed. For GT1s utilizing UDP-sugars, a reaction can be quenched at a given time point and the ejected UDP can then be enzymatically coupled to regenerate ATP from ADP. Subsequently, regenerated ATP can be used to generate a luminescent signal, by action of luciferase, that is directly proportional to the amount of UDP present at the time of reaction quenching. This UDP-coupled luciferase output forms the basis of the Uridine Diphosphate-Glo (UDP-Glo) assay for quantifying UDP via a stable luminescence signal.^30^ Using the UDP-Glo assay, we analyzed kinetic experiments and derived Michaelis–Menten parameters for TriX-QA, UDP-4-keto-6-deoxy-d-Glc, and UDP-d-Fuc (Table 1 and Figures S9 and S10).

**Table 1:** Kinetic Parameters of QsFucT and SvFucT for the Acceptor TriX-QA and UDP-Sugar Donors UDP-4-keto-6-deoxy-d-Glc and UDP-d-Fuc as Determined by the UDP-Glo Assay and Fit to the Michaelis–Menten Model^a^

	QsFucT			SvFucT		
substrate	*K*_M_ (μM)	*k*_cat_ (s^–1^)	*k*_cat_/*K*_M_ (mM^–1^ s^–1^)	*K*_M_ (μM)	*k*_cat_ (s^–1^)	*k*_cat_/*K*_M_ (mM^–1^ s^–1^)
TriX-QA	15 ± 1	1.4 ± 0.1	∼100	20 ± 1	19.1 ± 0.1	∼1000
UDP-4-keto-6-deoxy-d-Glc	58 ± 4	2.2 ± 0.1	∼40	46 ± 4	26.2 ± 0.2	∼600
UDP-d-Fuc	690 ± 110	1.1 ± 10	∼2	500 ± 80	13.3 ± 0.2	∼30

a Reaction velocity vs substrate concentration plots may be found in Figures S9 and S10. Parameters for TriX-QA were collected in the presence of an excess of UDP-4-keto-6-deoxy-d-Glc (600 μM). Reported error is the standard error. Experiments were performed in triplicate.

These data reveal that both QsFucT and SvFucT are unequivocally 4-keto-6-deoxy-d-glucosyltransferases, are biosynthetically isofunctional, and more effectively utilize UDP-4-keto-6-deoxy-d-Glc over UDP-d-Fuc by a factor of ∼20 based on the observed catalytic efficiencies (*k*_cat_/*K*_M_). While both enzymes bind their saponin acceptor and cognate UDP-sugar donor in the low-micromolar range, SvFucT appears to be a significantly faster enzyme that is approximately 10-fold more efficient than QsFucT at functionalizing TriX-QA with 4-keto-6-deoxy-d-Glc or UDP-d-Fuc. The observed kinetic properties of QsFucT are of similar magnitude to those of other characterized GT1s, while SvFucT appears to be exceptionally efficient.^31–35^ The enhanced catalytic properties of SvFucT may prove to be useful for optimizing the heterologous production of saponins or for enzymatic synthesis. Having finally revealed the cognate UDP-sugar for both enzymes, we next aimed to delineate the substrate scope with regard to the triterpene/saponin acceptor.

### Acceptor Substrate Scope of QsFucT and SvFucT

Previous investigations into the acceptor substrate scope of GT1s involved in C28-COOH *O*-d-fucosylation have been limited to in vivo transient expression of SOAP6 (from yossoside biosynthesis), UGT74CD1 (“SoFucT”, from saponarioside biosynthesis), SvFucT, and QsFucT in *N. benthamiana* pursuant to functionally characterizing enzymes involved in the biosynthesis of yossosides, saponariosides, vaccarosides, and QS-21/QS-7, respectively.^13,15–17^ In all four cases, biosynthetic intermediates were only observed to bear C28-COOH *O*-d-fucosylation if intermediates also bore at minimum C3-OH *O*-d-glucuronosylation (Figure 1B,C). These observations are consistent with saponins isolated from these producing plants in that no congeners have been characterized bearing C28-COOH *O*-glycosylation in the absence of C3-OH *O*-glycosylation and suggest that C3 tailoring is necessary and sufficient to enable C28-COOH *O*-glycosylation in vivo. Two competing hypotheses have been proposed to explain the apparent prerequisite for C3-OH *O*-glycosylation to precede C28-COOH *O*-glycosylation. The first is that C28 GTs recognize all or part of the C3-OH O-linked saccharide, with d-GlcA playing a crucial role in substrate binding, as it is necessary and sufficient to elicit fucosylation in planta. Alternatively, since the hydrophobic aglycones are thought to be sequestered to the endoplasmic reticulum (ER) and thus inaccessible to cytoplasmic enzymes (including the C28 GTs), the initial C3-OH *O*-d-glucuronosylation, performed by an integral transmembrane glucuronosyltransferase, may render the produced intermediate sufficiently hydrophilic to escape the ER and subsequently be transformed by cytoplasmic GTs.^15,17^ To probe these hypotheses, we tested the ability of both QsFucT and SvFucT to C28-COOH *O*-glycosylate a variety of triterpene acceptors, including quillaic acid (QA), C3-OH *O*-d-glucuronosylated oleanolic acid (calenduloside E, CE), and C3-OH 4-*O*-methyl-d-glucuronosylated gypsogenin (MeGlcA-Gyp), and analyzed their reaction products by LC-MS (Figure 3). The significant hydrophobicity of these acceptors required dissolving them in MeOH and adding them to the reaction mixture immediately before the addition of enzymes to mitigate the potential for precipitation of the substrate. QsFucSyn and NADPH were included in these reactions to avoid potential product inhibition by the 4-keto-6-deoxy-d-Glc-bearing intermediates. Surprisingly, both QsFucT and SvFucT were able to glycosylate each of the substrates tested. Most notably, quillaic acid was fully converted by both enzymes into a species with a mass consistent with that of C28-COOH *O*-d-fucosylated quillaic acid. Taken together, these results suggest that C3-OH *O*-glycosylation preceding C28-COOH *O*-glycosylation in saponin biosynthesis is likely due to spatial separation of C28-COOH GTs from the hydrophobic aglycone, which remains sequestered in the ER until acted on by the integral transmembrane glucuronosyltransferase. Additionally, oxidations at C23 and C16, present in quillaic acid but not in calenduloside E, are dispensable, suggesting that oleanolic acid would be the minimalistic substrate for QsFucT and SvFucT.

**Figure 3:**
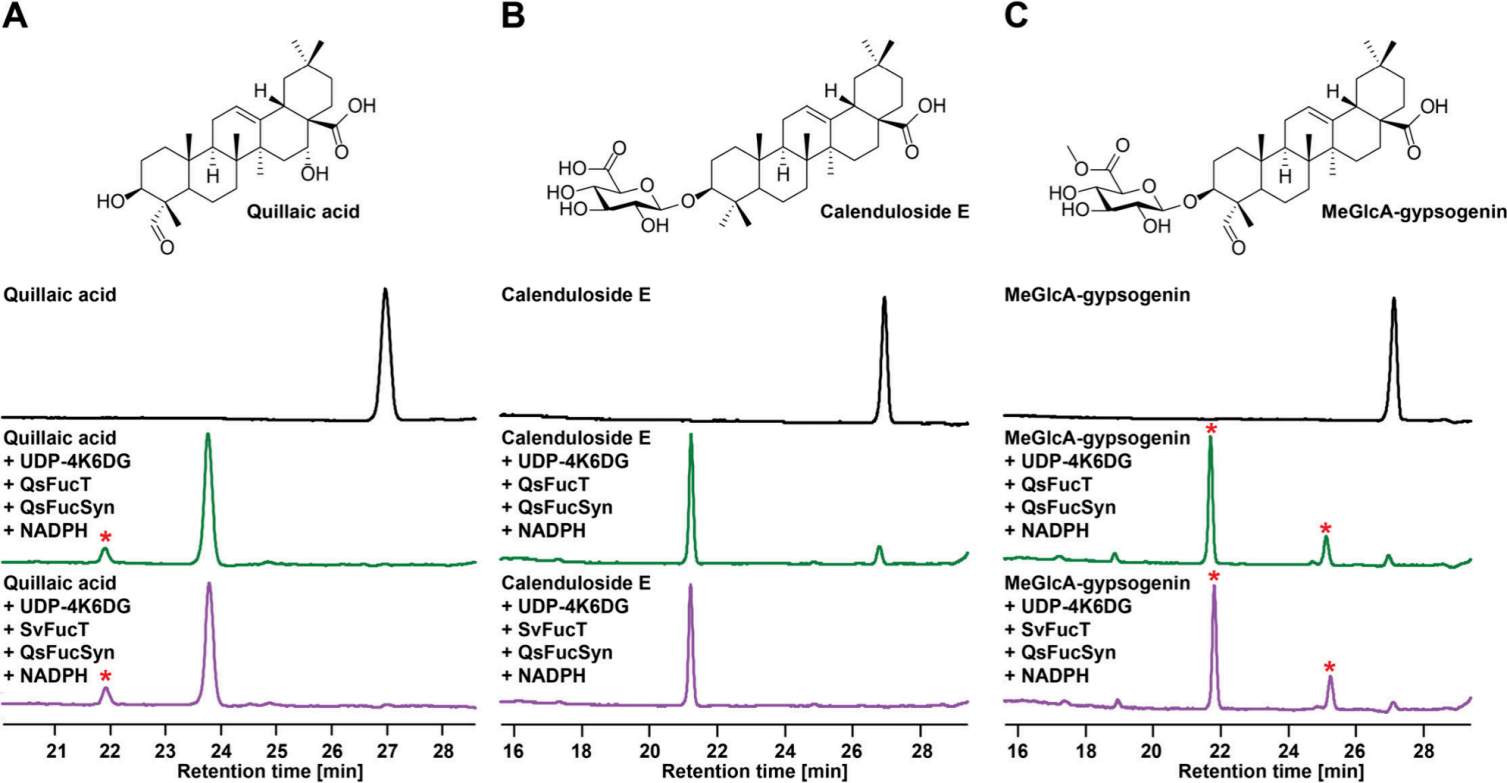
**In vitro glycosylation assays on triterpene/saponin acceptors by QsFucT and SvFucT.** Shown are A220 HPLC chromatograms of starting material or reaction products for reactions of QsFucT or SvFucT with (A) quillaic acid, (B) calenduloside E, or (C) MeGlcA-gypsogenin. Red asterisks denote unexpected product peaks with masses corresponding to a formal reduction (+2 Da) of starting material or fucosylated product. Mass spectra for the observed products may be found in Figure S11. UDP-4K6DG, UDP-4-keto-6-deoxy-d-Glc.

### The C3 Trisaccharide of TriX-QA Prevents Spurious Reduction of the C23 Aldehyde by QsFucSyn

In the course of performing acceptor substrate scope studies, product peaks were observed with masses 2 Da heavier than the expected fucosylated product for QA and MeGlcA-gypsogenin, suggesting that an unexpected formal reduction had occurred; in the case of MeGlcA-gypsogenin, the major product had an observed mass consistent with MeGlcA-gypsogenin being both fucosylated and formally reduced. Furthermore, a formally reduced starting material was also observed. In contrast, no additional product peak was observed for acceptors TriX-QA and calenduloside E (Figures 2, 3, and S11). Common to all tested acceptors is a C11–C12 double bond, while QA and MeGlcA-QA also bear a C23 aldehyde group. We hypothesized that a spurious reduction occurred at either the alkene or aldehyde and was mediated either chemically by the large excess of NADPH or enzymatically by the reductase QsFucSyn. To probe this phenomenon, previously tested acceptors (TriX-QA, QA, MeGlcA-gypsogenin, and CE) as well as additional oleanane aglycones bearing differential oxidation at C16 and C23 (oleanolic acid [OA], echinocystic acid [EA], hederagenin, and gypsogenin) were tested for reduction by NADPH alone or in the presence of QsFucSyn. While no product peaks were observed in reactions with NADPH alone for any acceptor, all tested acceptors bearing a C23 aldehyde with the exception of TriX-QA (QA, MeGlcA-gypsogenin, and gypsogenin) produced a product peak with a mass +2 Da from the starting material (Figures 4 and S12). In contrast, all acceptors lacking a C23 aldehyde (CE, OA, EA, and hederagenin) were completely nonreactive toward QsFucSyn (Figure S12). These data are consistent with QsFucSyn spuriously reducing the C23 aldehyde to the corresponding alcohol. This explanation is further corroborated by the product of gypsogenin being chemically indistinguishable from hederagenin (Figure S12E).

**Figure 4:**
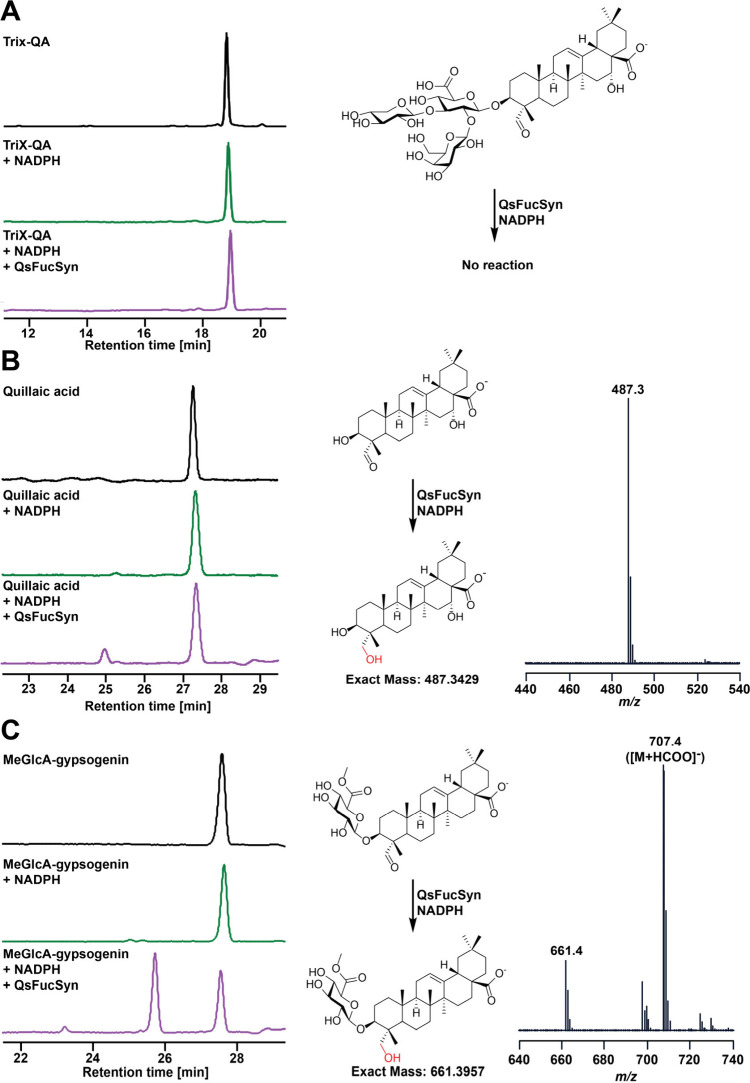
**In vitro acceptor reduction assay by QsFucSyn.** Shown are A220 HPLC chromatograms of starting material or reaction products, chemical structures of reactants and products (shown as deprotonated species observed by ESI-MS), and mass spectra for products if observed. (A) TriX-QA nonreaction with QsFucSyn. (B) QA reaction with QsFucSyn. (C) MeGlcA-gypsogenin reaction with QsFucSyn. MeGlcA-gypsogenin’s product MS base peak corresponds to a formate adduct. Additional tested acceptors are listed in Figure S12.

Of the tested substrates, MeGlcA-QA was the most efficiently reduced by QsFucSyn compared to aglycones QA and gypsogenin, suggesting that aqueous solubility may play a role in substrate accessibility and demonstrating that the C3 *O*-glucuronic acid alone cannot prevent spurious reduction of the C23 aldehyde. In stark contrast, zero reduction was observed for TriX-QA (Figure 2B,C). These observations suggest that the C3-OH branched saccharide of saponariosides, vaccarosides, and QS-21/QS-7 may play a conserved role in sterically protecting the C23 aldehyde from erroneous reduction during their biosynthesis. This steric protection may have ramifications for the biosynthetic or chemoenzymatic synthesis of new-to-nature saponins where a C23 aldehyde is desired in the absence of a branched C3-OH *O*-trisaccharide and may require engineering of QsFucSyn (or related homologues) to impart more stringent regioselectivity.

### UDP-Sugar Substrate Scope of QsFucT and SvFucT

We next sought to evaluate the ability of QsFucT and SvFucT to utilize additional noncognate sugars beyond UDP-d-Fuc. Given that the C28-COOH *O*-linked linear saccharide of QS-21/QS-7 is an indispensable component of the “core pharmacophore” required for immunomodulatory activity, the ability to install noncognate sugars would provide a facile means of glycodiversifying these important therapeutics and generating novel analogs with potentially altered bioactivity, toxicity, or physicochemical properties. While characterized plant GT1s have shown considerable promiscuity with regard to acceptor substrate, these GTs often only efficiently recognize their cognate UDP-sugar donor.^18^ To delineate the UDP-sugar substrate scope of QsFucT and SvFucT, we performed in vitro glycosylation assays on TriX-QA using a large excess (5 mM, 25 molar equiv relative to TriX-QA acceptor) of various noncognate UDP-sugar donors and analyzed the reaction products by LC-MS. Both QsFucT and SvFucT were able to utilize a variety of UDP-sugars, including hexoses (d-Glc, d-Gal, d-Fuc), pentoses (l-Ara*p*, l-Ara*f*, d-Xyl), and unexpectedly, 6-azido-6-deoxy-d-Glc. Neither GT demonstrated activity with the UDP-uronic acids d-GlcA and d-GalA (Table 2 and Figures 2, S13, and S34).

**Table 2:** Conversion of TriX-QA to the Corresponding C28-COOH *O*-Glycosylated Product by QsFucT or SvFucT Using Various Noncognate UDP-Sugars^a^

	conversion (%)	
UDP-sugar	QsFucT	SvFucT
UDP-d-Glc	69.2	71.7
UDP-d-Gal	74.0	74.9
UDP-l-Rha	0	47.1
UDP-d-Fuc	81.3	83.2
UDP-d-Xyl	74.0	73.2
UDP-l-Ara*p*	83.6	86.1
UDP-l-Ara*f*	12.7	19.6
UDP-d-GlcA	0	0
UDP-d-GalA	0	0
UDP-d-GlcNAc	0	4.9
UDP-d-GlcNAz	1.2	51.8
UDP-6-azido-6-deoxy-d-Glc	79.9	80.6

a Percent conversion was estimated by taking the ratio of the A220 peak area for the product to the sum of the product and residual starting material peak areas. Product chromatograms and mass spectra may be found in Figures S13–S34.

Surprisingly, although they are biosynthetically isofunctional in their native contexts, several clear differences in the substrate scope emerged comparing QsFucT and SvFucT. Specifically, SvFucT was able to effectively utilize UDP-l-Rha and UDP-d-GlcNAz (*N*-azidoacetylglucosamine) and had trace activity utilizing UDP-d-GlcNAc, while QsFucT had no activity using UDP-l-Rha or UDP-d-GlcNAc and extremely trace (∼1%) conversion utilizing UDP-d-GlcNAz.

We next performed a more granular kinetic analysis for both enzymes with UDP-sugars that showed appreciable (>45% conversion) glycosylation activity. All of the tested sugars presented *K*_M_ values in the moderate- to high-micromolar range with stark differences in catalytic efficiency (Table 3). Consistent with previous nonkinetic glycosylation assays, UDP-l-Ara*p* is the most well-tolerated of the tested UDP-sugars, ostensibly due to its structural similarity with UDP-d-Fuc (i.e., l-Ara*p* is C5-desmethyl-d-Fuc). A general trend noted for both enzymes is that UDP-sugars with axial C4 hydroxyl groups (i.e., d-Gal, d-Fuc, l-Ara*p*, and l-Rha) had consistently higher reaction velocities than UDP-sugars with equatorial C4 hydroxyl groups (i.e., d-Glc and d-Xyl) (Table 3 and Figures S35 and S36). In other characterized GT1s, UDP-sugar C4 stereochemistry has been demonstrated to be important in substrate recognition and is mediated in part by interactions with the terminal residue of the PSPG.^18,23^ Glucosyltransferases typically present a terminal His residue, while galactosyltransferases present Gln. Both QsFucT and SvFucT, with PSPGs terminating in Gln, are consistent with these previous studies due to their preferential use of UDP-sugars with axial C4 hydroxyl groups (Figure S2). However, our data also suggest that substitution at C6 may play a crucial role for both QsFucT and SvFucT UDP-sugar recognition, as evidenced by the lack of reactivity toward UDP-uronic acids as well as the presence of a visible reaction velocity plateau for UDP-6-azido-6-deoxy-d-Glc which was not observed for UDP-d-Glc (Figures S35 and S36).

**Table 3:** Kinetic Parameters of QsFucT and SvFucT for Noncognate UDP-Sugar Donors for the C28-COOH *O*-Glycosylation of TriX-QA^a^

	QsFucT			SvFucT		
UDP-sugar donor	*K*_M_ (μM)	*k*_cat_ (s^–1^)	*k*_cat_/*K*_M_ (mM^–1^ s^–1^)	*K*_M_ (μM)	*k*_cat_ (s^–1^)	*k*_cat_/*K*_M_ (mM^–1^ s^–1^)
UDP-d-Glc	N.D.^b^	N.D.^b^	N.D.^b^	N.D.^b^	N.D.^b^	N.D.^b^
UDP-d-Xyl	280 ± 20	0.4 ± 0.1	∼2	370 ± 50	2.0 ± 0.0(2)	∼5
UDP-6-azido-6-deoxy-d-Glc	330 ± 40	0.0(07) ± 0.1	∼0.0(2)	330 ± 90	0.4 ± 0.1	∼1
UDP-d-GlcNAz	N.D.^c^	N.D.^c^	N.D.^c^	N.D.^b^	N.D.^b^	N.D.^b^
UDP-d-Gal	N.D.^b^	N.D.^b^	N.D.^b^	N.D.^b^	N.D.^b^	N.D.^b^
UDP-l-Ara*p*	380 ± 20	1.7 ± 0.1	∼5	790 ± 100	12.2 ± 1.0	∼15
UDP-l-Rha	N.D.^c^	N.D.^c^	N.D.^c^	820 ± 90	7.9 ± 0.1	∼10

a Kinetic curves may be found in Figures S35 and S36. Experiments were performed in triplicate. N.D., not determined.

b A reaction velocity plateau was not observed in kinetic assays.

c UDP-sugar was not kinetically assayed because of insignificant turnover in glycosylation assays.

These results demonstrate that both QsFucT and SvFucT exhibit considerable flexibility in UDP-sugar substrate scope and could be leveraged to glycodiversify saponin intermediates toward generating novel analogs. These enzymes could also be utilized for rapid semisynthetic derivatization of saponins or their attachment to antibodies using click chemistry (i.e., through azide–alkyne cycloaddition). Laboratory evolution experiments to improve specificity and enzymatic efficiency toward noncognate UDP-sugar donors could enable these GTs to be utilized as efficient in vitro catalysts or employed for scalable in vivo heterologous production.

### Crystal Structures of QsFucT and SvFucT in Complex with UDP

The crystal structures of QsFucT-UDP and SvFucT-UDP were solved at 2.1 and 1.7 Å resolution, respectively (Figure 5 and Table S4). The general architecture of QsFucT and SvFucT present a typical GT-B fold, which is characterized by two discrete N-terminal and C-terminal Rossmann-like domains joined by a loop region, resulting in a cleft that forms the active site (Figure 5A).^36^ The N-terminal domain comprises residues 1–231 and 1–246, while the C-terminal domain comprises residues 259–457 and 276–473 for QsFucT and SvFucT, respectively; the intervening residues form the interdomain loop. Consistent with other GT1 structures, there is a high level of the overall conserved tertiary structure. Structural changes are more evident in the N-terminal domain for residues proximal to the active site, which have evolved to accommodate different acceptors.^37^ Superposition of the solved structures for QsFucT and SvFucT based on the position of α carbons showed an RMSD of 1.658 Å and highlights the highly conserved GT-B fold.

**Figure 5:**
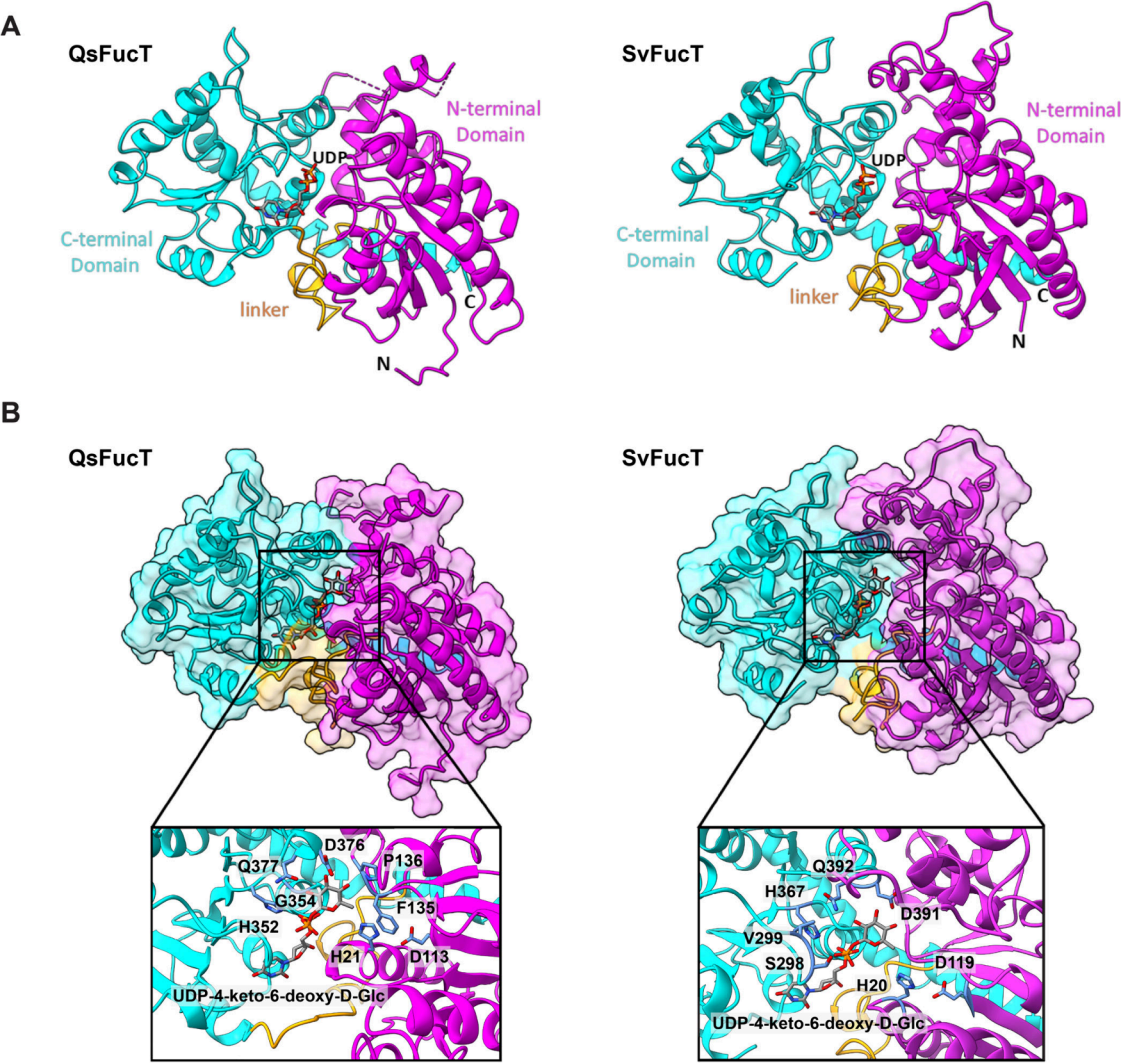
**Structural analysis of QsFucT and SvFucT.** (A) Cartoon representations of the overall crystal structures of QsFucT (left) and SvFucT (right) bound to UDP. Structural features shown include the N-terminal domain (magenta), the C-terminal domain (cyan), and the interdomain linker (orange). The UDP nucleotide is shown in stick representation and resides in a cleft between the N-terminal and C-terminal domains. (B) Surface models of QsFucT (left) and SvFucT (right) with cognate substrate UDP-4-keto-6-deoxy-d-glucose modeled into the active site. The positions of UDP in the solved crystal structure were used to position UDP-4-keto-6-deoxy-d-Glc. The insets depict a zoomed-in view of the UDP-sugar binding site for both enzymes, detailing residues that are anticipated to be in contact with the UDP-sugar and may play a role in substrate discrimination. A more detailed depiction of the shown active sites may be found in Figure S38.

As members of the GT1 family, the peptide sequences of QsFucT and SvFucT feature a conserved His-Asp dyad thought to play a critical role in facilitating catalysis (Figures 1D and S2). Our crystallographic data of these enzymes confirmed that these residues are both positioned in the same active site position compared to other crystal structures of GT1 family members and demonstrate a hydrogen bond between the carboxylate oxygen of Asp and the Nδ1-H of His that is 2.2 and 2.3 Å in QsFucT and SvFucT, respectively (Figure 5B). To probe the importance of these conserved residues in adding 4-keto-6-deoxy-d-Glc onto the oleanane core, we generated variants of QsFucT and SvFucT where the putative catalytic dyad residues were substituted with alanine and presumed to interfere with the canonical GT1 mechanism (Figure 1D): QsFucT H21A, QsFucT D113N, SvFucT H20A, and SvFucT D119N. These variants were assayed for their ability to glycosylate TriX-QA. Under our standard assay conditions, which utilizes 20 μM enzyme, all four variants tested were approximately as active as their wild-type counterparts, with the exception of SvFucT H20A, which demonstrated slightly less conversion (Figure S37A,B). This result was initially surprising, given the critical role these residues play in the catalytic mechanism of other GT1s. We hypothesized that a lack of observable impact when substituting these residues may be masked by the unnaturally high enzyme concentrations in our assays, and therefore, we performed these assays again using either 200 nM QsFucT or 20 nM SvFucT (0.1% and 0.01% catalyst loading, respectively). Under these conditions, both wild-type enzymes were able to fully convert TriX-QA; however, both enzymes had substantially less conversion when the active site histidine was substituted (25.6% for QsFucT H21A and 52.6% for SvFucT H20A). Furthermore, QsFucT D113N demonstrated 73.3% conversion, while the corresponding SvFucT variant (D119N) was insensitive to this substitution (Figure S37C,D). Similar observations have been made for isoflavone 7-*O*-glucosyltransferase GmlF7GT, which glycosylates the phenolic C4-OH of the flavonoids genistein and daidzin, and UGT74F2, which glucosylates the phenolic hydroxyl or the carboxylic acid of salicylic acid. As with QsFucT and SvFucT, both GmlF7GT and UGT74F2 showed diminished, but not ablated, in vitro activity when the dyad histidine was substituted with alanine; notably, UGT74F2 was only able to produce the glucosyl ester via COOH glucosylation.^32^ Similar to the carboxylic acid acceptor of TriX-QA, the carboxylic acid of salicylic acid and the phenolic C4-OH of genistein and daidzin are expected to have a significant population of deprotonated species under physiological conditions (p*K*_a_ of C4-OH = 7.2 and 7.5 for genistein and daidzin, respectively) while the phenolic hydroxyl of salicylic acid would be essentially exclusively protonated (p*K*_a_ = 13.6).^38,39^ Taken together, these data suggest that the catalytic histidine is important for efficient glycosylation under physiologically relevant conditions but is dispensable under the unnaturally high enzyme loading achievable in vitro and may reflect that ester-forming GT1s lacking the catalytic histidine can still function by placing a predeprotonated nucleophile in near-attack conformation. This hypothesis is further supported by other GT1s that act on less acidic acceptors absolutely requiring the catalytic histidine for conversion.^19,33,40^

### Analysis of the UDP-Sugar Binding Site of QsFucT and SvFucT

Residue interactions between GT1s and their UDP-sugar donors are key determinants in determining substrate affinity and the stringency of substrate scope.^41^ To interrogate these linchpin interactions, we attempted to soak crystals of QsFucT and SvFucT with ligands UDP-4-keto-6-deoxy-d-Glc or UDP-d-Fuc. In all cases, electron density was only observed for UDP with a notable absence of any density for the sugar moiety, suggesting that the crystallized enzyme remains active and is capable of hydrolyzing UDP-sugars with efficiency that precludes acquiring holo enzymes bearing density of a full UDP-sugar. Similar results have been observed for crystallography endeavors with other GT1s.^42^ To obviate this limitation and probe which residues may be involved in UDP-sugar donor specificity, models of QsFucT and SvFucT with UDP-4-keto-6-deoxy-d-Glc docked were created by inferring the sugar position based on the bona fide coordinates of bound UDP. These models revealed possible interactions between the enzymes and the UDP-sugar. In QsFucT, residues His352 (coordinated by Thr351) and Ser357 may be involved in hydrogen bonding to the diphosphate, Asp376/Gln377 may be involved in hydrogen bonding to the sugar, and Phe135/Pro136/Gly354 may contribute hydrophobic interactions. Analogously, in SvFucT, residues His367 (coordinated by Thr366) and Ser372 may hydrogen-bond to the diphosphate, Asp391/Gln392 may hydrogen-bond to the sugar, and Ser298/Val299 may provide hydrophobic interactions (Figures 5B and S38).

To probe the importance of potential interactions, we substituted each with alanine and assayed their ability to glycosylate TriX-QA using lowered enzyme concentrations, as was done for substitutions to the catalytic dyad. While none of the substitutions totally ablated enzymatic activity, several resulted in lower conversion (Figure S39 and Table S5). In QsFucT, Thr351 coordinates His352, which appears to provide crucial interactions with the diphosphate of UDP, and alanine substitution of these residues resulted in 51.8% and 36.3% conversion, respectively. In contrast, homologous substitutions in SvFucT elicited full conversion, suggesting that these interactions may play a greater role in sugar binding in QsFucT compared to SvFucT. Conversely, substitution of Ser357 in QsFucT still resulted in full conversion, whereas substitution of Ser372 in SvFucT resulted in 76.6% conversion; this may be attributable to the hydrogen bonding between the Thr hydroxyl and the diphosphate group of UDP observable in SvFucT but absent in QsFucT. Most notably, substitution of Asp376 or Gln377 in QsFucT or Asp391 or Gln392 in SvFucT resulted in dramatically reduced turnover (19.8% for QsFucT D376A, 42.1% for QsFucT Q377A, 25.7% for SvFucT D391A, and 31.1% for SvFucT Q392A). These residues constitute the final two residues of each enzyme’s PSPG and are thought to form linchpin interactions with the C3 and C4 substituents of the sugar. Alanine substitution of these final PSPG residues in GT1s GmlF7GT, VvGT1, and UGT71G1 resulted in complete or near-complete loss of catalytic activity.^19,31,32^ The retention of activity by QsFucT and SvFucT suggests that these interactions, while important, are not the sole drivers for sugar binding. Precise investigation of binding interactions by QsFucT and SvFucT will require crystallization of a binary or ternary complex and, because of the ability of the crystallized enzyme to nonproductively hydrolyze the UDP-sugar donor, may entail the synthesis of a nonhydrolyzable analog such as one that replaces the glycosidic bond with a phosphonate linkage.

## Conclusions

Saponins are an expansive class of glycosylated triterpene NPs with remarkable bioactivities, foremost being the immunomodulatory activity of QS-21, which has saved countless lives due to its incorporation in vital vaccine formulations. Several classes of saponins (QS-21/QS-7, yossosides, vaccarosides, and saponariosides) feature C28-COOH *O*-d-fucosylation as a critical transformation in the biosynthetic maturation of these compounds and, in the case of QS-21 and QS-7, are an integral part of the “core pharmacophore” that gives rise to adjuvant bioactivity. Due to their chemical complexity, namely, intricate glycosylation patterns and abundance of stereocenters, saponin chemical space remains challenging to rapidly explore to seek novel analogs with enhanced properties. Engineered enzymatic (bio)­synthesis is a promising avenue for glycodiversifying these important medicinal NPs; however, these endeavors are predicated on detailed enzymological knowledge of each biosynthetic transformation.

In this work, we performed enzymological and structural biology studies on two GTs involved in the C28-COOH *O*-d-fucosylation of saponins: QsFucT from QS-21/QS-7 biosynthesis and SvFucT from vaccaroside biosynthesis. In vitro glycosylation reactions revealed that both enzymes, despite their names, operate as UDP-4-keto-6-deoxy-d-glucosyltransferases and esterify 4-keto-6-deoxy-d-Glc onto the C28 carboxylic acid of a triterpene/saponin core prior to reduction to afford d-Fuc. Because of this, we suggest that care be taken when referring to these enzymes and that an alternate name (e.g., 4K6DGlcT) may be warranted. Furthermore, we demonstrated that both enzymes have fairly relaxed substrate scopes regarding the triterpene acceptor, seeming to require only a β-amyrin triterpene skeleton featuring a C28 carboxylic acid (i.e., oleanolic acid). These experiments also revealed that the conserved branched trisaccharides of QS-21/QS-7, vaccarosides, and saponariosides may play a role in sterically protecting the C23 aldehyde from spurious reduction during biosynthesis. Substrate promiscuity was also observed with regard to the UDP-sugar donor, where both enzymes were able to utilize a variety of sugars, including those bearing azido functionalities, albeit significantly less efficiently compared to the cognate UDP-sugar substrate. Lastly, we performed X-ray crystallography for both enzymes, providing the founding crystallographic structures for UDP-4-keto-6-deoxy-d-glucosyltransferase GT1s, and utilized these structural data to probe the importance of conserved residues in the active site.

Collectively, these data paint an encouraging picture for engineering saponin 4-keto-6-deoxy-d-glucosyltransferases to effectively utilize alternate triterpene acceptors or UDP-sugar donors as part of saponin glycodiversification campaigns. The intrinsic malleability of both QsFucT and SvFucT with regard to acceptor and donor substrates renders both enzymes ideal candidates for modern in silico evolution approaches such as low-*N* zero-shot methods (e.g., EVOLVEpro^12^) to quickly survey protein sequence space and identify variants with enhanced specificity toward noncognate substrates. Both in silico evolution and activity prediction will undoubtedly be aided by the availability of bona fide crystallographic structures, and we anticipate that this study will accelerate future saponin discovery and engineering efforts.

## Supplementary Material



## Notes

Materials and methods, gene sequences and oligonucleotide primers, SDS-PAGE gel of proteins, additional HPLC­(MS) chromatograms and associated mass spectra, enzyme kinetics data, enzyme variant conversion data, and crystallographic refinement data (DOCX)

## Notes

The crystallographic structures of QsFucT and SvFucT have been deposited in the Research Collaboratory for Structural Bioinformatics Protein Data Bank (RCSB PDB) under accession codes 9PF3 and 9PFA, respectively.

## ◇

School of Chemistry, Pharmacy and Pharmacology, University of East Anglia, Norwich Research Park, Norwich NR4 7TQ, U.K., and Chemistry Department, Faculty of Science, Mansoura University, Mansoura 35516, Egypt
