# Supplementary material for: Enzymology and Structural Basis of Glycosyltransferases Involved in Saponin C28 Carboxylic Acid O‑d‑Fucosylation
Source: JACS Au. 2025 Nov 24;5(12):6011–24. doi: 10.1021/jacsau.5c00907 (PMC12728629; doi:10.1021/jacsau.5c00907)
Supplement: Supplementary file 1 [file au5c00907_si_001.docx]

**Supporting Information for Publication**

**Enzymology and Structural Basis of Glycosyltransferases Involved in Saponin C28 Carboxylic Acid *O*-D-fucosylation**Graham A. Hudson^a,b,c^, Jose H. Pereira^a,d^, Peter H. Winegar^a,b,c^, David M. FitzGerald^a,b,d^, Andy DeGiovanni^a,e^, Xiaoyue Chen^a,f,g^, Xixi Zhao^a,b,c^, Maria C.T. Astolfi^a,b,h^, James Reed^i^, Amr El-Demerdash^i,l,m^, Martin Rejzek^i^, Shingo Kikuchi^i^, Anne Osbourn^i^, Henrik V. Scheller^a,f,g^, Paul D. Adams^a,e,j^, and Jay D. Keasling^a,b,c,j,k^*

^a^ Joint BioEnergy Institute, Lawrence Berkeley National Laboratory, Emeryville, CA, 94608, USA

^b^ Biological Systems and Engineering, Lawrence Berkeley National Laboratory, Berkeley, CA, 94720, USA

^c^ California Institute for Quantitative Biosciences (QB3 Institute), University of California, Berkeley, CA, 94720, USA

^d^ Department of Molecular and Cell Biology, University of California, Berkeley, CA, 94720, USA

^e^ Molecular Biophysics and Integrated Bioimaging Division, Lawrence Berkeley National Laboratory, Berkeley, CA, 94720, USA.

^f^ Environmental Genomics and Systems Biology Division, Lawrence Berkeley National Laboratory, 1 Cyclotron Road, Berkeley, CA, 94720, USA

^g^ Department of Plant and Microbial Biology, University of California, Berkeley, CA, 94720, USA

^h^ Department of Bioengineering, University of California Berkeley, Berkeley, CA, 94720, USA.

^i^ John Innes Centre, Norwich Research Park, Norwich, NR4 7UH, UK

^j^ Department of Chemical and Biomolecular Engineering, University of California, Berkeley, CA, 94720, USA

^k^ The Novo Nordisk Foundation Center for Biosustainability, Technical University Denmark, Kemitorvet, Building 220, Kongens, Lyngby, 2800, Denmark

^l^ Current address: School of Chemistry, Pharmacy and Pharmacology, University of East Anglia, Norwich Research Park, Norwich NR4 7TQ, UK.

^m^ Current address: Chemistry Department, Faculty of Science, Mansoura University, Mansoura-35516-Egypt

* Corresponding author:

Jay D. Keasling ([keasling@berkeley.edu](mailto:keasling@berkeley.edu)), phone: 1-510-642-4862, fax: 1-510-495-2630

**Table of Contents**

[**Experimental Methods** 3](#_Toc212027266)

[**Table S1:** Sequences of synthesized genes used in this study. 8](#_Toc212027267)

[**Table S2:** Sequences of oligonucleotide primers used in this study. 10](#_Toc212027268)

[**Figure S1:** SDS-PAGE analysis of proteins used for enzymatic studies. 11](#_Toc212027269)

[**Figure S2**: Multiple sequence alignment of glycosyltransferases involved in saponin C28 fucosylation. 12](#_Toc212027270)

[**Table S3:** Similarity-identity matrix of glycosyltransferases involved in saponin C28 fucosylation. 13](#_Toc212027271)

[**Figure S3:** LC-MS purity analysis of TriX-QA from QS-21 hydrolysis. 14](#_Toc212027272)

[**Figure S4:** In vitro enzymatic synthesis of UDP-4-keto-6-deoxy-D-Glc from UDP-D-Glc. 15](#_Toc212027273)

[**Figure S5:** LC-MS purity analysis of enzymatically synthesized, purified UDP-4-keto-6-deoxy-D-Glc. 16](#_Toc212027274)

[**Figure S6:** In vitro enzymatic synthesis of UDP-D-Fuc from UDP-D-Glc. 17](#_Toc212027275)

[**Figure S7:** LC-MS purity analysis of enzymatically synthesized, purified UDP-D-Fuc. 18](#_Toc212027276)

[**Figure S8:** Mass spectrometry analysis of QsFucT and SvFucT reaction products using UDP-4-keto-6-deoxy-D-Glc or UDP-D-Fuc. 19](#_Toc212027277)

[**Figure S9:** Kinetic assays of QsFucT with TriX-QA, UDP-4-keto-6-deoxy-D-Glc, UDP-D-Fuc. 21](#_Toc212027278)

[**Figure S10:** Kinetic assays of SvFucT with TriX-QA, UDP-4-keto-6-deoxy-D-Glc, UDP-D-Fuc. 22](#_Toc212027279)

[**Figure S11:** Mass spectra for products observed in Main Text Figure 3. 23](#_Toc212027280)

[**Figure S12:** In vitro reduction assay by FucSyn on various terpene/saponin acceptors. 24](#_Toc212027281)

[**Figure S13:** In vitro glycosylation of TriX-QA with UDP-D-Glc by QsFucT. 26](#_Toc212027282)

[**Figure S14:** In vitro glycosylation of TriX-QA with UDP-D-Gal by QsFucT. 27](#_Toc212027283)

[**Figure S15:** In vitro glycosylation of TriX-QA with UDP-L-Rha by QsFucT. 28](#_Toc212027284)

[**Figure S16:** In vitro glycosylation of TriX-QA with UDP-D-Xyl by QsFucT. 29](#_Toc212027285)

[**Figure S17:** In vitro glycosylation of TriX-QA with UDP-L-Ara*p* by QsFucT. 30](#_Toc212027286)

[**Figure S18:** In vitro glycosylation of TriX-QA with UDP-L-Ara*f* by QsFucT. 31](#_Toc212027287)

[**Figure S19:** In vitro glycosylation of TriX-QA with UDP-D-GlcA by QsFucT. 32](#_Toc212027288)

[**Figure S20:** In vitro glycosylation of TriX-QA with UDP-D-GalA by QsFucT. 33](#_Toc212027289)

[**Figure S21:** In vitro glycosylation of TriX-QA with UDP-D-GlcNAc by QsFucT. 34](#_Toc212027290)

[**Figure S22:** In vitro glycosylation of TriX-QA with UDP-D-GlcNAz by QsFucT. 35](#_Toc212027291)

[**Figure S23:** In vitro glycosylation of TriX-QA with UDP-6-azido-6-deoxy-D-Glc by QsFucT. 36](#_Toc212027292)

[**Figure S24:** In vitro glycosylation of TriX-QA with UDP-D-Glc by SvFucT. 37](#_Toc212027293)

[**Figure S25:** In vitro glycosylation of TriX-QA with UDP-D-Gal by SvFucT. 38](#_Toc212027294)

[**Figure S26:** In vitro glycosylation of TriX-QA with UDP-L-Rha by SvFucT. 39](#_Toc212027295)

[**Figure S27:** In vitro glycosylation of TriX-QA with UDP-D-Xyl by SvFucT. 40](#_Toc212027296)

[**Figure S28:** In vitro glycosylation of TriX-QA with UDP-L-Ara*p* by SvFucT. 41](#_Toc212027297)

[**Figure S29:** In vitro glycosylation of TriX-QA with UDP-L-Ara*f* by SvFucT. 42](#_Toc212027298)

[**Figure S30:** In vitro glycosylation of TriX-QA with UDP-D-GlcA by SvFucT. 43](#_Toc212027299)

[**Figure S31:** In vitro glycosylation of TriX-QA with UDP-D-GalA by SvFucT. 44](#_Toc212027300)

[**Figure S32:** In vitro glycosylation of TriX-QA with UDP-D-GlcNAc by SvFucT. 45](#_Toc212027301)

[**Figure S33:** In vitro glycosylation of TriX-QA with UDP-D-GlcNAz by SvFucT. 46](#_Toc212027302)

[**Figure S34:** In vitro glycosylation of TriX-QA with UDP-6-azido-6-deoxy-D-Glc by SvFucT. 47](#_Toc212027303)

[**Figure S35:** Kinetic assays of QsFucT with non-cognate UDP-sugar donors. 48](#_Toc212027304)

[**Figure S36:** Kinetic assays of SvFucT with non-cognate UDP-sugar donors. 49](#_Toc212027305)

[**Figure S37:** In vitro glycosylation of TriX-QA by QsFucT and SvFucT catalytic dyad substitution variants. 51](#_Toc212027306)

[**Table S4:** Crystallographic statistics data for refinement of QsFucT and SvFucT 52](#_Toc212027307)

[**Figure S38:** Detailed depiction of the active sites of QsFucT and SvFucT. 53](#_Toc212027308)

[**Figure S39:** In vitro glycosylation of TriX-QA by additional active site variants. 54](#_Toc212027309)

[**Table S5:** Conversion data of tested QsFucT and SvFucT active site variants. 55](#_Toc212027310)

[**Supporting Information References** 56](#_Toc212027311)

Experimental Methods

**General materials and methods**. In the course of all experiments described, no unexpected or unusually high safety hazards were encountered. General reagents used for molecular biology experiments were purchased from New England Biolabs (NEB, Ipswitch, MA), ThermoFisher Scientific (Waltham, MA), or Gold Biotechnology (St. Louis, MO). UDP-sugars were purchased from BioSynth (Compton, England, UK), MedChemExpress (Monmouth Junction, NY), or Glycodepot (Atlanta, GA). Triterpenes and saponins were purchased from MedChemExpress. For all experiments, stock solutions of TriX-QA and QS-21 were dissolved in water at a concentration of 1 mg/mL while other triterpenes/saponins were dissolved in MeOH at a concentration of 1 mg/mL. UDP-sugar stock solutions were dissolved in water at a concentration of 10 mM.

**Molecular biology techniques**. Oligonucleotides and synthesized genes were purchased from Integrated DNA Technologies (Coralville, IA). Genes for QsFucT, SvFucT, WsbK, and QsFucSyn were codon optimized for expression in *Escherichia coli* while genes for ATCV-1-UG46DH and SvNMD were codon optimized for expression in *Saccharomyces cerevisiae* using Integrated DNA Technology’s codon optimization tool; sequences for synthesized genes may be found in **Table S1** and oligonucleotide primer sequences may be found in **Table S2**. Polymerase chain reactions (PCRs) to amplify the pET28-MBP plasmid backbone or genes were performed using NEB Q5 Hot Start polymerase according to the manufacturer’s instructions. PCRs were separated on a 1.1% agarose gel and amplicon sizes compared to the GeneRuler 1kb plus ladder (ThermoFisher). Bands containing the desired amplicons were individually excised from the gel and purified using the Zymoclean Gel DNA Recovery Kit (Zymo Research, Irvine, CA). Amplicons were ligated into the pET28-MBP backbone by Gibson Assembly using NEBuilder HiFi DNA Assembly master mix (NEB) according to manufacturer instructions. The assembled plasmids were then transformed into XL1-Blue chemically competent *E. coli*, plated on lysogeny broth (LB) agar plates supplemented with 50 μg/mL kanamycin, and incubated for 24 h at 37 °C. Single colonies were used to inoculate 5 mL of LB supplemented with 50 μg/mL kanamycin which was then incubated with shaking for 16 h at 37 °C. Cells were harvested and the plasmids isolated using the QIAGEN QIAprep Spin Miniprep Kit (QIAGEN, Redwood City, CA). Purified plasmids were sequence verified using Next Generation Sequencing by Plasmidsaurus (Arcadia, CA).

**MBP-tagged enzyme overexpression and purification**. *E. coli* BL21(DE3) harboring a pACYC-Duet plasmid containing the Cpn10 and Cpn60 chaperonins from psychrophile *Oleispira antarctica* were transformed with a pET28 plasmid encoding the MBP-tagged enzyme intended for overexpression and purification. Cells were grown for 24 h on LB agar plates supplemented with 50 μg/mL kanamycin at 37 °C. Single colonies were used to inoculate 10 mL of LB supplemented with 50 μg/mL kanamycin and 34 μg/mL chloramphenicol and grown at 37 °C for ~16 h. This culture was then used to inoculate 500 mL of Terrific Broth (24 g/L yeast extract, 12 g/L tryptone, 0.4% glycerol (v/v), 17 mM KH_2_PO_4_, and 72 mM K_2_HPO_4_) supplemented with 38 μg/mL kanamycin and 25 μg/mL chloramphenicol and grown to an optical density at 600 nm (OD_600_) of ~1.5 before being placed on ice for 15 min. Protein expression was then induced by the addition of 0.4 mM isopropyl β-D-1-thiogalactopyranoside (IPTG); 2 mM MgSO_4_ was also added at this time to enhance terminal cell density. Protein overexpression was carried out for 24 h at 12 °C. Subsequently, cells were harvested by centrifugation (4,000 relative centrifugal force (r.c.f.) for 20 min). Cell pellets were transferred to 50 mL conical tubes, flash frozen in liquid nitrogen, and stored at -80 °C for a maximum of one week prior to affinity purification.

Cell pellets were resuspended in lysis buffer (50 mM Tris-HCl pH 7.5, 150 mM NaCl, 2.5% glycerol (v/v), and 0.1% Triton X-100 (v/v)) containing 4 mg/mL lysozyme. Resuspended cells were incubated at 4 °C on a nutator for 30 min at which point cells were further homogenized by sonication (three rounds of 45 s with 10 min nutation periods at 4 °C). Insoluble debris was removed by centrifugation (45,000 r.c.f. for 45 min). The supernatant was then applied to a pre-equilibrated amylose resin (~10 mL of resin). After lysate had flowed through the column, the column was washed with 10 column volumes of lysis buffer supplemented with 0.5 mM tris-(2-carboxyethyl)-phosphine (TCEP) followed by 10 column volumes of wash buffer (lysis supplemented with 0.5 mM TCEP but lacking Triton X-100). MBP-tagged proteins were eluted using elution buffer (50 mM Tris-HCl pH 7.5, 300 mM NaCl, 2.5% glycerol (v/v), 10 mM maltose, 0.5 mM TCEP). The eluent was concentrated using an appropriate molecular weight cut-off (MWCO) Amicon Ultra centrifugal filter (EMD Millipore) and subsequently buffer exchanged four times with 10× volumes of protein storage buffer (50 mM HEPES pH 7.5, 300 mM NaCl, 2.5% glycerol (v/v), 0.5 mM TCEP). Protein concentrations were determined using a NanoDrop ND-1000 spectrophotometer (Thermo Scientific, Waltham, MA) by absorbance at 280 nm and using computed extinction coefficients from the ExPASy ProtParam tool; <https://web.expasy.org/protparam/>).^1^ Protein purity and truncation were assessed by Coomassie-stained SDS-PAGE (**Figure S1**). Purified protein was aliquoted, flash frozen in liquid nitrogen, and stored at -80 °C until used for in vitro reactions.

**Small scale in vitro fucose enzymatic synthesis assay**. The ability for SvNMD or wskB to reduce UDP-4-keto-6-deoxy-D-Glc to UDP-D-Fuc was tested in vitro using 10 mM UDP-D-Glc, 10 mM NAD^+^, 10 mM NADH, 10 mM NADPH, 100 μM MBP-ATCV-1-UG46DH, and 10 μM MBP-SvNMD or MBP-WsbK in GT assay buffer in a 1.7 mL Eppendorf tube (50 μL final reaction volume). The reaction was incubated at room temperature for 16 h. The enzymatic reaction was quenched and enzymes precipitated by the addition of four volumetric equivalents of MeOH. The precipitated enzymes were removed by centrifugation (21,000 r.c.f. for 5 min) and the supernatant transferred to a new 1.7 mL Eppendorf tube. The supernatant was dried under vacuum using a CentriVap benchtop concentrator (LabConco, Kansas City, MO). Reaction products were dissolved in 50 μL H_2_O prior to analytical LC-MS analysis.

**Analytical LC-MS of UDP-sugars**. UDP-4-keto-6-deoxy-D-Glc and UDP-D-Fuc were analyzed by high-performance liquid chromatography mass spectrometry (HPLC-MS) on an Agilent 1260 Infinity II HPLC equipped with an Agilent MSD iQ single quadrupole detector. 2 μL of reconstituted reaction product was injected onto and separated using a HyperCarb porous graphitic column (150 x 1 mm, 5 μm particle size, Thermo Scientific, 35005-151030) held at 50 °C, operating at a flow rate of 0.1 mL/min, and separated using the following gradient with eluent A consistent of 0.3% (v/v) ammonium formate pH 9.0 and eluent B consisting of MeCN:

| **Time (min)** | **%B** |
| --- | --- |
| 0 | 2 |
| 20 | 15 |
| 26 | 50 |
| 27 | 90 |
| 30 | 90 |
| 31 | 2 |
| 50 | 2 |

The mass detector settings were as follows: 13.0 L/min drying gas, 350 °C drying gas temperature, 60 psi nebulizer gas pressure, and -6000 V capillary voltage. UDP-sugars were detected by mass spectrometry using negative mode scan 100 to 1000 m/z with a fragmentor voltage of 110 V as well as by monitoring absorbance at 220 and 260 nm. Data were analyzed using OpenLab CDS Data Analysis utility (Agilent).

**Scaled enzymatic synthesis of UDP-4-keto-6-deoxy-D-Glc.** UDP-4-keto-6-deoxy-D-Glc was enzymatically synthesized by combining 10 mM UDP-D-Glc, 10 mM NAD^+^, and 72 μM MBP-ATCV-1-UG46DH in GT assay buffer in a 1.7 mL Eppendorf tube (1400 μL final reaction volume) and incubated at room temperature for 3 h. The enzymatic reaction was quenched and enzymes precipitated by the addition of four volumetric equivalents of MeCN. The precipitated enzymes were removed by centrifugation (21,000 r.c.f. for 5 min) and the supernatant transferred to a 50 mL conical vial. The supernatant was then flash frozen in liquid nitrogen before being freeze dried on a FreeZone 6 lyophilizer (LabConco). UDP-4-keto-6-deoxy-D-Glc was then dissolved in water and purified by HPLC using a porous graphitic column as described below to afford 5.2 mg of UDP-4-keto-6-deoxy-D-Glc diammonium salt (~68% yield).

**Scaled enzymatic synthesis of UDP-D-Fuc.** UDP-D-Fuc was enzymatically synthesized by combining 10 mM UDP-D-Glc, 10 mM NAD^+^, 10 mM NADPH, 10 μM MBP-ATCV-1-UG46DH, and 10 μM MBP-WsbK in GT assay buffer in a 50 mL conical tube (2.4 mL final reaction volume) and incubated at room temperature for 16 h. The enzymatic reaction was quenched and enzymes precipitated by the addition of four volumetric equivalents of MeOH. The precipitated enzymes were removed by centrifugation (21,000 r.c.f. for 5 min) and the supernatant transferred to a 50 mL conical vial. The supernatant was dried under vacuum using a CentriVap benchtop concentrator (LabConco). The resulting solid was dissolved in water and purified by HPLC using a porous graphitic column as described below to afford 9.2 mg of UDP-D-Fuc diammonium salt (~78% yield).

**Scaled HPLC purification of UDP-sugars**. Enzymatically synthesized UDP-4-keto-6-deoxy-D-Glc were purified using an Agilent 1260 HPLC system equipped with a HyperCarb porous graphitic column (150 x 4.6 mm, 5 μm particle size, Thermo Scientific, 35005-154630) operating at a flow rate of 1 mL/min and separated using the following gradient with eluent A consisting of 0.3% ammonium formate pH 9.0 and eluent B consisting of MeCN:

| **Time (min)** | **%B** |
| --- | --- |
| 0 | 2 |
| 20 | 15 |
| 26 | 50 |
| 27 | 90 |
| 30 | 90 |
| 31 | 2 |
| 50 | 2 |

Fractions containing the product were collected by hand by monitoring absorbance at 260 nm and verified to contain product by direct infusion into an Agilent iQ MSD detector (UDP-4-keto-6-deoxy-D-Glc Rt = 17.0 min and UDP-D-Fuc Rt = 13.6 min). UDP-4-keto-6-deoxy-D-Glc eluted as a smear, likely due to rapid equilibrium between the keto-form and gem-diol hydrate. Fractions containing the purified UDP-sugar were flash frozen in liquid nitrogen before being freeze dried on a FreeZone 6 lyophilizer (LabConco). The resulting dried powder was assayed for yield gravimetrically and, after being dissolved in water, verified spectrophotometrically using a NanoDrop ND-1000 spectrophotometer (Thermo Scientific) by A260 absorbance (using the reported extinction coefficient for UDP of 9.8 × 10^3^ M^-1^ cm^-1^).^2^

**Base hydrolysis of QS-21 to produce TriX-QA**. Approximately 5 mg of QS-21 (MedChemExpress) was dissolved in 1 mL of 345 mM potassium hydroxide in a 2 mL screw-cap vial and incubated at 80 °C for 6 h on a heat block. The reaction was then neutralized with an equal volume of 345 mM formic acid before being applied to a 2000 mg C_18_ HyperSep SPE cartridge (ThermoFisher) equilibrated with water. The flow through was collected and the column washed with 5 mL of water followed by 5 mL volumes of 5%, 25%, 50%, 75%, and 100% MeCN. Elution fractions were analyzed by HPLC-MS and the fractions containing TriX-QA (5% and 25%) were combined and dried under vacuum in a CentriVap benchtop concentrator (LabConco), yielding approximately 2.2 mg of TriX-QA (~92% yield).

**In vitro glycosylation assay using UDP-4-keto-6-deoxy-D-Glc and UDP-D-Fuc.** In vitro glycosylation was carried out in 1.7 mL Eppendorf tubes using 20 μM MBP-QsFucT or MBP-SvFucT, 200 μM TriX-QA, and 1 mM of UDP-4-keto-6-deoxy-D-Glc or UDP-D-Fuc in GT assay buffer with a final volume of 25 μL. For reactions containing QsFucSyn as indicated, MBP-QsFucSyn and NADPH were added to final concentrations of 20 μM and 2 mM, respectively. Reactions were allowed to incubate at room temperature for 16 h. Subsequently, enzymes were precipitated by the addition of 100 μL of MeOH followed by vortexing. Precipitated enzymes were removed by centrifugation (21,000 r.c.f. for 5 min.), transferred to a new 1.7 mL Eppendorf tube and dried under vacuum in a CentriVap benchtop concentrator (LabConco). The dried supernatants were resuspended in 25 μL of 80% MeOH using a combination of vortex resuspension and sonication before being transferred to a sample vial for LC-MS analysis. For assessing the activity of alanine-substituted variants, the enzyme concentration was adjusted to 200 nM or 20 nM for QsFucT or SvFucT, respectively, unless otherwise noted.

**In vitro glycosylation assay using non-cognate UDP-sugars.** In vitro glycosylation was carried as described above with the exception of using 5 mM non-cognate UDP-sugar (UDP-D-Glc, UDP-D-Gal, UDP-D-Xyl, UDP-6-azido-6-deoxy-D-Glc, UDP-D-GlcNAz, UDP-L-Ara*p*, UDP-L-Ara*f*, UDP-L-Rha, UDP-D-GlcA, or UDP-D-GalA).

**In vitro reduction assays by NADPH or QsFucSyn.** In vitro reduction assays were carried out in 1.7 mL Eppendorf tubes using 20 μM MBP-QsFucSyn, 200 μM triterpene/saponin, and 2 mM NADPH in GT assay buffer with a final volume of 25 μL. Control reactions to test for reduction by NADPH alone omitted MBP-QsFucSyn. Reactions were incubated at room temperature for 16 h. Subsequently, enzymes were precipitated by the addition of 100 μL of MeOH followed by vortexing. Precipitated enzymes were removed by centrifugation (21,000 r.c.f. for 5 min.), transferred to a new 1.7 mL Eppendorf tube and dried under vacuum in a CentriVap benchtop concentrator (LabConco). The dried supernatants were resuspended in 25 μL of 80% MeOH using a combination of vortex resuspension and sonication before being transferred to a sample vial for LC-MS analysis.

**LC-MS analysis of in vitro glycosylation or reduction reactions**. In vitro glycosylation samples were analyzed by HPLC-MS on an Agilent 1260 Infinity II HPLC equipped with an Agilent MSD iQ single quadrupole detector. 10 μL of sample was injected onto and separated using an EC UHPLC Nucleodur C_18_ Htec column (100 x 2 mm, 1.8 μm particle size, Machery-Nagel, Allentown, PA; part number 760306.20) at ambient temperature operating at a flow rate of 0.3 mL/min and using the following gradient with eluent A consisting of 0.1% formic acid and eluent B consisting of MeCN with 0.1% formic acid:

| **Time (min)** | **%B** |
| --- | --- |
| 0 | 15 |
| 1.5 | 15 |
| 26 | 60 |
| 26.5 | 100 |
| 33 | 100 |
| 35 | 15 |
| 50 | 15 |

The mass detector settings were as follows: 11.0 L/min drying gas, 325 °C drying gas temperature, 50 psi nebulizer gas pressure, and -3500 V capillary voltage. Glycosylation reactants and products were detected by mass spectrometry using negative mode scan from 400 to 1450 m/z with a fragmentor voltage of 120 V as well as by monitoring absorbance at 215 nm. Data were analyzed using OpenLab CDS Data Analysis utility (Agilent) and conversion estimated by dividing the A_215_ product peak area by the sum of A_215_ peak areas for product and starting material.

**Kinetic assay for saponin acceptor.** Kinetic parameters for MBP-QsFucT and MBP-SvFucT with regard to the saponin acceptor (TriX-QA) were acquired using the UDP-Glo Kit (Promega, Madison, WA; part number V6961). The UDP-Glo kit uses UDP produced by the glycosyltransferase reaction to regenerate ATP and, via luciferase, generate a stable luminescent signal that is proportional to the amount of UDP. On a 96-well PCR plate, TriX-QA was serially diluted 12 times in GT reaction buffer containing 600 μM UDP-4-keto-6-deoxy-D-Glc from a starting concentration of 1 mM in triplicate, providing a series of TriX-QA concentrations ranging from 500 nM to 1000 μM. Next, 4 μL of the serial dilutions were transferred to a Hard-Shell 384-well PCR plate with white wells (Bio-Rad, Hercules, CA; part number HSP3805) and the glycosyltransferase reaction was initiated by adding 1 μL of glycosyltransferase to a final concentration of 50 nM for MBP-QsFucT or 5 nM MBP-SvFucT. After 10 minutes of incubation at 25 °C, the glycosyltransferase reaction was quenched with 5 μL of UDP-Glo Nucleotide Detection Reagent. Blanks containing the acceptor and UDP-sugar were also reacted with UDP-Glo Nucleotide Detection Reagent to enable subtraction of background signal from any UDP present that was not a reaction product. In addition, serially diluted UDP solution (starting concentration 50 μM) was added to the 384-well plate and reacted with Nucleotide Detection Reagent. The plate was incubated at 25 °C for 60 minutes before luminescence measurement using a BioTek Synergy H1 plate reader. The luminescence probe height was calibrated before each reading using the luminescence signal from 50 mM UDP. Luminescence data points for high concentrations of UDP-sugar exhibiting a significantly decreased reaction velocity relative to the next dilution in the series were assumed to be exhibiting substrate inhibition and were thus excluded from analysis.^3^ Background-subtracted luminescence vs. TriX-QA concentration data were plotted in OriginPro 2022b and non-linear curve fitting performed using the Michaelis-Menten function with the Orthogonal Distance Regression iteration algorithm, providing K_M_ and V_max_. Calculated V_max_ values were converted to concentration of UDP using the UDP standard curve and used to calculate k_cat_.

**Kinetic assay for UDP-sugar donors.** Kinetic parameters for MBP- QsFucT and MBP-SvFucT with regard to UDP-sugar donors were acquired as described above with the following modifications. The concentration of TriX-QA was held constant at 100 μM and the starting concentration of the UDP-sugar being assayed was 1,000 μM, 2,500 μM, or 5,000 μM for UDP-4-keto-6-deoxy-D-Glc, UDP-D-Fuc, and all other sugars, respectively. Blanks containing the UDP-sugar dilution series but lacking enzyme were also reacted with UDP-Glo Nucleotide Detection Reagent to enable subtraction of background signal from any UDP present that was not a reaction product. The final concentration of MBP-QsFucT or MBP-SvFucT in glycosyltransferase reactions was 12.5 nM or 2.5 nM, respectively.

**Expression and Purification of FucT for Crystallization.** *E.coli* BL21(DE3) were transformed with a pET28 plasmid encoding the TEV-cleavable MBP-tagged FucT enzyme intended for overexpression and purification. Cells were grown for 24 h on LB agar plates supplemented with 50 μg/mL kanamycin at 37 °C. Single colonies were used to inoculate 10 mL of LB supplemented with 50 μg/mL kanamycin and grown at 37 °C for ~16 h. This culture was then used to inoculate 1 L of Terrific Broth supplemented with 50 μg/mL kanamycin and grown at 37 °C until an OD_600_ of ~1.0. The flasks were chilled on ice to 20 °C. Protein expression was then induced by the addition of 0.4 mM IPTG. The flasks were then cultured at 200 rpm and 20 °C for approximately 20 h. The frozen *E. coli* pellet was re-suspended in 5 mL/g of 25 mM HEPES pH 7.4, 250 mM NaCl + 2 mM MgCl_2_. 0.3 ug/mL DNaseI, 0.1 mg/mL lysozyme and 1x CalBiochem EDTA free Protease Inhibitor Cocktail Set-V. The re-suspended cells were lysed using the Avestin EmulsiFlex-C3 homogenizer followed by clarification at 40,000 r.c.f. for 40 minutes. The clarified lysate was loaded onto a 5 mL HisTrap column on an AKTA PURE FPLC instrument. The His-tagged fusion protein was eluted by a gradient of 4-50 %B in 20 CV. The eluted fusion protein was cleaved by TEV protease with dialysis to 25 mM HEPES, pH 7.4, 250 mM NaCl to remove the imidazole. The dialyzed, cleaved protein was put through a 5 mL HisTrap column to separate the MBP from the FucT. The majority of the cleaved FucT bound and eluted from the column with 50 mM imidazole. The eluate was put through a 5mL MBPTrap column to remove the remaining un-cleaved fusion protein. The FucT was then further purified by injection on a 10x300 S75 Increase column (Cytiva) that was equilibrated in 25 mM HEPES pH 7.4, 250 mM NaCl. The cleanest elution fractions were pooled, dialyzed to 25 mM HEPES pH 7.4, 150mM NaCl and concentrated to 10.4 mg/mL.

**Crystallization, X-Ray data collection and structure determination of QsFucT and SvFucT.** QsFucT and SvFucT samples were concentrated to 10.4 and 10.3 mg/ml, respectively. QsFucT and SvFucT were screened against the crystallization set of solutions: Berkeley Screen,^4^ MCSG-1 (Anatrace), ShotGun (Molecular Dimensions), PEG/Ion, Index, Crystal Screen, and PEGRx (Hampton Research). Crystals of QsFucT were found in MCSG-1 screen condition B2 composed of 0.2 M sodium chloride, 0.1 M BIS-Tris (pH 5.5) and 25% (w/v) PEG 3,350. Crystals of SvFucT were found in Berkeley screen condition B9 composed of 0.2 M magnesium chloride, 0.1 M Bis-Tris (pH 6.5) and 25% (w/v) PEG 3,350. The cofactor UDP was soaked with the crystals of QsFucT and SvFucT for 3 h at 10 mM final concentration. The crystals of QsFucT and SvFucT were placed in a reservoir solution containing 20% (v/v) glycerol, then flash-cooled in liquid nitrogen. The X-ray data set for QsFucT and SvFucT were collected at the Berkeley Center for Structural Biology beamlines 8.2.2 and 5.0.1 at the Advanced Light Source at Lawrence Berkeley National Laboratory. The diffraction data were processed using the program Xia2.^5^ The crystal structure of QsFucT and SvFucT were solved by molecular replacement with the program PHASER^6^ using as an initial coordinates model generated by ALPHAFOLD.^7^ The atomic positions obtained from the molecular replacement were used to initiate refinement using within the Phenix suite.^8^ Structure refinement was performed using the phenix.refine program.^9^ Manual rebuilding was done using COOT.^10^ Root-mean-square deviations from ideal geometries for bond lengths, bond angles, and dihedral angles were calculated with phenix.refine.^9^ The stereochemical quality of the final model of QsFucT and SvFucT were assessed by the program MOLPROBITY.^11^ Summary of crystal parameters, data collection, and refinement statistics can be found in **Table S4.**

Table S1: Sequences of synthesized genes used in this study.

All sequences are provided 5′ to 3′. NCBI protein accessions are provided in parentheses.

QsFucT (KAJ7950116.1):

ATGGAAAATGGGCGTGTATATAAGTCACATGTAGTTGTTCTTGCCTTTCACGGGCAGGGACATATCGTCCCGCTTATCCAATTAAGCCGCCGTCTGGCTTGGAAGGGGATTAAGATTACTTTTGCCACGACGCACTCTTGCACGAAAGCTATCCAGACGGGCTCAGACTCAATCTCGCTTCTGTCCATCTATGATGACATTACAGATGGAGGGTTTCAAGGAGAAGGAGGTTTTAAAGGCTTTCTGCAGCGCTTTGAGGCATCCACCACACGCATCTTACATGAGTTTGTCAAAAACCACGAAAATTCGAAGAATCCAGTTAAGTGTTTGATTTATGATGCGAATCTTATCTGGGCTTTAGAAATGGCAAAACAGCTGGGGATTGCGACGGCGGCCTTTGTCTTCCCATCCTGGGCCGCAATTGCGACGTACTACCCTTTTTATTTAGAGGTTTACGCCGACCAACAAATCAAAAAAGTCGACCCGTTTACGATGCCGGACCTTCCTCCGCAATTGGGTTTACCGAATATGGCGAGTCTTGGATCTGACAGTGGGCAGCACTCTCCAATTCTTAAGCTTATGTTGCAGCAACTGGAAAACTTTGGAAAGGCCGACTGGATTCTGTCACACGCCTTCGAGCAGTTTGAACAGGAGGTTCTTGATTGGATGCGCAACATTAGTCCCGTCACCACCATTGGCCCTACTCTTCCCAGTGTGTATCTGGACGGTCGTTTGAAAGATGATACTGATTATGGCTACAACCTGTATAAGCCTGATTCAGATACGTGCATGAAGTGGTTGGATACAAAAGAAACTGAGTCGGTAGTTTACATTTCTTTCGGGAGCGTTGCCGACCTGATCCCCGAACAGATGACTGAAATCACAAATAGTCTGAAAAAAATGTCCAGTAACTTCTTATGGGTTGTTAAGGAGACGGAGAAAAACAACCTGCCGTCAAGTTTCGTCGAAGAGACAAAGGAAAAAGGTTTAGTAGTCACGTGGTGCCCGCAACTTAAAGTCCTTAGCCACCCTGCCGTAGGGTGCTTTATCACGCATTGTGGTACTAACTCTATCTTCGAATCGGTTTGCTTCGCTGTACCGATGGTTGGTATGCCGCAATTCTGTGACCAGATGCCTAATGCTTATTTCATGGAGAAAGTGTGGAAGGTAGGTGTACGCCCGTCATTGGACGACAACGGTGTAGTAACAGGGGAAGAAATTGAGCGTTGCATTAAGGTTGTAACGGAAGGTGAGAGCGGACAAGAAATTAAAAAGAAGCTTGTACAGTGGAAGGAATTAGCTAAGGAGGCCGTGGATGAGGGCGGCTCGTCCGACAAACACATTGACGAATTTATTGCAGGGATTACTACTTAA

SvFucT (unannotated; sequence from Chen et al.)^12^

ATGGCTCATGAGATTAAAAAGATCGAAATTATTGTTTTTCCTTACCATGGACAGGGCCACATGAACACAATGTTGCAATTCGCAAAACGTTTAGCGTGGAAAAATGTGGTCAACGTGACCATCGCAACCACCTTGGGTACTACGAACAAGATGCGCTCACAGGTCGACCAAGTCGATATTAATAGCGCGTGGGCTGCGTCGATTGACCTTGTAAGCGTGTACGATGATACAGACGAGTCGCAGGTGCGCTTCATGGACCGTATGGCTCGTTTTGAAGCGGCTGCGGCCTCAAATTTAGCCACCTTATTAGTCAAAAAAAAAGAACACGCGGACAACAAGGTGCTGTTGGTATATGATGGGAACCTGCCTTGGGCATTGGACGTTGCTCACGGGAAAGGAGTTCGCGGGGCTGCCTTCTTTCCCCAGAGCTGTGCAACAGTTGCGACTTACTATTGCTTGTATAATGAGACCCAAGGGCGCGAAGTAGAGCCCAAATTGCCAGATGTATTTCCCCCTCTTGAGCTGATCCAGCGTAATATCCCTAATGTATATGGAATGAAGTTTCCTGAAGCAGTAGTCCCTAAAAATGGGAAAGAGTATTCGGATTTTGTGTTGTACGTTTTGCGCCAGTGTGTCAACCTGGAGAAATCCGATTTAGTGCTTTTCAATCAATTCGACAAGTTAGTAGAACCGGGGGAGGTCTTACAGTGGATGAGTAAGATCTTTAATGTTAAGACAATCGGTCCTACTCTGCCATCCGCCTATATCGATAAGCGCATCAAAGACGACGTTGCTTACGGATTCCACGCATTCAACTTGGACAATGACACATGCATGAACTGGTTGAGCACCAAGCCTGCTGGATCAGTAATCTATATCGCATTCGGGAGCAGCGTTCACTATTCGGTCGAGCAAATGACCGAAATCGCCGAGGCTTTGCGCTCCCAGCCCAAGAATTTCCTTTGGGCGGTACGTGAGACCGAGCAGGTAAAGCTTCCCGCCGACTTCATCGAACAGACAGCCGACAAGGGGCTGGTATTGGCTTGGTGCCCCCAGTTGGACGTACTGAAACACGAGAGCATCGGCTGTTTTGTAACACATTGCGGCTGGAATTCAATTACTGAAGCGTTAAGCTTCGGGGTGCCTATGCTGTCCGTGCCGCAGTTTCTTGACCAGCCAGTTGACGCGCACTTCGTGGAACAAGTTTGGGGGGCCGGAGTGACAGTTAAGCGTAATGAGGACGGGTTTGTAACTCGCGACGAGATCATCCGCTGCTTGGACGAGCTTAACGATGGAGAGCGTGCTGCTGAGATTAAGGCCAACGTGGCACGTTGGAAGGTGTTGGCCAAAGAAGCGTTGGAGGAAGGGGGATCATCTGACAAGCATATTGATGAGATTATTGAATGGGTGAGTTCTTTCTGA

ATCV-1-UG46DH (YP_001427025.1)

ATGAATTCCCAAGAGTACACACCCAAAAGCGTATTGGTGACGGGTGGAGCGGGTTTTATCGGGTCACACGTGGTTATGAAACTTGTTCAAAGGTATCCCGAGTGCAAAGTTGTAGTTCTTGACAAAATGGATTATTGTGCTACGTTGAACAATCTAGCGACGGTCAGAGATGCCCCAAACTTCAAGTTTGTCAAAGGCGATATTCAAAGTACAGATCTTCTAGCTCATGTTCTTAAGCAGGAGAAAATAGATACGATTATGCACTTTGCCGCTCAAACACATGTTGACAACAGCTTCGGCAATAGCCTTGCATTCACGATGAATAACGTGTATGGCACTCACGTTCTATTAGAATGTGCTAGGCTATATGGCGGCGTTCAAAGGTTCATAAATGTTAGCACGGATGAGGTCTATGGCGAATCCAGCTTGGGAAAAAAAGAAGGCTTGGATGAGCACTCCAGCTTAGAGCCGACTAACCCCTACGCAGCCGCAAAGGCGGGGGCTGAAATGATGGCTAGGGCATACCATACAAGTTATAAACTTCCTGTTATTGTGACCCGTGGGAACAACGTGTATGGTCCACATCAATTTCCGGAGAAAATGATTCCTAAGTTTATATTGCGTGCAACGAGAGGTTTGGACCTGCCTATTCACGGAGACGGTGGGGCATTAAGGAGCTACCTATATGTCGATGACGTGGCCGAGGCCTATATAACCATACTTCTGAAGGGGAACGTGGGTGAGACTTATAATATCGGAACTCAAAAGGAGAGATCCGTAGTAGACGTGGCGCACGATATTTGCAAAATTTTTAACCGTGACTCTGATACTGCAATATGGCACGTCAAAGATAGGGCCTTTAACGATCGTCGTTACTTCATTAGCGATAAGAAGTTATTAGATCTAGGATGGCAGGAAAAGACCACGTGGGAGGATGGGCTGAAGCAAACTGTGGGATGGTATCTACAACATGCAACTAGGAGTTACTGGGATCATGGTAACATGGAATTGGCATTAGACGCCCATCCAACATTGCAGGTTCCAAAATTCTAG

SvNMD (unannotated; sequence from Chen et al.)^12^

ATGGCCGAGGCTCCCTCCTTCCTAGCCCAGAAAAGGTATGCCGTGGTTACAGGAGCAAACAAGGGCTTGGGTCTAGAAATCTCACGTCAACTAGCCTCTCAGGGGGTCGTTGTACTATTAACCAGTCGTGATGAAAAGCGTGGATTCGAAGCCATAGAAGAACTAAAAAAGAGCGGCATAAACTCAGAAAATTTAGTATATCATCAGCTAGATGTAACGAATCCGAGCAGTTTCGCCAGTTTAGCTGATTTCGTAAAGGCTAAGTTTGGCAAGTTAGATGTGCTAGTTAATAACGCGGGGATATCCGGCGTCATTGTGGACTATCCTGCCCTAATGGAAGCGATAAGGAACCGTGGCGCAGAAATAAACTATGACGGGGTAATGAAGCAGACGTATGAACTGGCTGAGGAATGCCTACAGACAAACTATTACGGTGTGAAAAGAACAATCAATGCACTGTTACCTCTTCTACAGCTATCAGATTCCCCCAGGATCGTTAACGTCAGTAGTGATGTTGGACTGTTGAAGAAAATCCCGGGTGAGAGGATAAGGGAAGCATTAGGCAACGCAGAAAAGCTAACTGAGGAGTCTGTGGATGGTTTGCTGGATGAGTTCTTGACTGATTTTAAGGAGGGCAAGATTGCCGAGAACGGTTGGCCTACGTTCAAGTCAGCATATAGTATTTCTAAAGCAGCACTTAACTCTTACACACGTGTATTAGCAAGAACATACCCGAGTATCATTATAAATTGCGTTTGCCCGGGTGTTGTTAAAACAGACATAAACCTGAAAATGGGACACTTGACTGTGGAAGAAGGAGCGGTTGGCCCCCTGAGGCTTGCACTACTGCCACCTGGAAGTCCGTCCGGGCTTTTTTACATGAAGAATGAAATTGCCTCCTTCGAGTGA

WsbK (WP_183252727.1)

ATGAAGCGCATTCTTATTCTTGGTTGCGGATACTTAGGCTTAAACTTGGCGAACTACTTCTGTAAAAAGAACTATGACGTGTCCGTAATCGGTCGCAAGTCCGTTTATAGCAACTTCTTAGAAGAGGAGATCGAATTTATTGAGGACGATATCAAGAATATTAACTCATATAAACACATGTTTAACGAGGAGACGACTGTTATTTACGCGATCGGGTCAATTAACGCTAACAATTACTTTATGGACCTGCGTAATGACATCGAGAATTCCTATATCCCATTTATTAACCTGCTGAATTTCTTGTCGGAGAAGTACATCCAGAAATTCGTTTTTTTATCAAGTGCGGGTACAGTCTATGGTAATGTAAACAAAAATTACATTAGCGAAAACGAGATTCTGAACCCAATTAGCATCTATGGGTTGCAAAAGGCCTTCTTCGAACAATTAATCCGCATCAAGAACAACGAGGCTTCACACTTCCGTTATCTGATCTTCCGCATTTCAAACCCCTATGGCGGTATTAATATTCCCAACAAGAACCAGGGAATCATTCCCACCCTGGTGTACAAGGCCGTCAACAATGAACCATTTGAATTGTGGGCTTCCATCAACACGATTCGCGACTATATCTACATCGACGACTTAAGCGAATTGATCTACAAAACAATTTATTTGGACATTTACAACGAAACATTGAACTTAGGCTCAGGAAAAGGCACTTCTATTAAGCAGCTTATCTCCTTGGTGGAAGAAATCTTGGGCAAAAAGATTACTATCCTTGAAAAGCCACCGATCAAAACGAACGTTCTGAAAAACATCCTGGACATCAGCAAACTGGTGAATACGGTCGGGTACGAGCCGAAAATTTCAATCGAGGAAGGCATTAGCCGTTACATTAATACGATTCTGACCAAGAACATTTTTTAA

QsFucSyn (KAJ7950118.1)

ATGGCAGAGGCTACGCAGCGTTATGCGGTTGTGACCGGGAGCAATAAAGGCATCGGGTTCGGTATTTGCAAGCAGTTAGCAAGTAAAGGGATTAAGGTAGTGTTGACCGCGCGTGACGAAAAGCGTGGCTTGGAGGCCGTTGAAAAATTGAAGGAAATCTCATTGGCTGGTCACGTTGTATTCCATCAGTTAGATGTGTCGGACCCAGCCTCGGTTACATCTCTTGAAGATTTTATTAAAACACAGTTTGGAAAACTTGATATTTTAGTAAATAACGCCGGGATTACGGGAACAACTGTTGATGCCGACGCATTAGCCGCATCCGGATTTGGAACCGGAGGTGAGCGCAAGCCAATTGATTGGTCTAAATTGGTAATTCAAACCTACGAATCTGTTGAGAAGGCTTTCAATACTAACTACTATGGTGGAAAACGCATGACGGAAGCTCTTATTCCGTTGTTACAATTAAGCGACTCCCCGCGTATTGTTAACGTTTCATCGGCTATGGGTCAGCTGGAAAACATCCCAAGCGGGTGGGCGAAGGAGGTTTTGACAGACGTCGATAACTTGACGGAGGGGAAGCTGGATGAGGTTAGCACTCAGTTCTTAAAAGATTTTAAGGAAGGATCCTTAGAAACGAAGGGTTGGCCGTCCTTGATGTCCTCTTACATTGTGAGTAAAGCCGTTTTGAATGCATACACACGTATTTTAGCGAAAAAGTACCCGGCATTTTGCATTAACTGCGTTGATCCCGGGTACGTCAAGACTGATATTAACCACCACACGGGCCAGCTGAGTGTTGACGAGGGTGCCGAATCTCCCGTCCGTCTGGCCCTTCTTCCGAACGGGGGCCCCTCTGGGGTTTTTTTTAGCCGTACAGAAGAGGCACCGTTCTAA

Table S2: Sequences of oligonucleotide primers used in this study.

All sequences are provided 5′ to 3′.

| **Primer name** | **Oligonucleotide sequence** |
| --- | --- |
| pET28-MBP_Backbone_F | CTCGAGCACCACCACCACC |
| pET28-MBP_Backbone_R | GGATCCTTGGAAGTATAGATTTTCACCCTTG |
| QsFucT_F | GAAAATCTATACTTCCAAGGATCCATGGAAAATGGGCGTGTATATAAGTC |
| QsFucT_R | GGTGGTGGTGGTGCTCGAGTTAAGTAGTAATCCCTGCAATAAATTCGTC |
| SvFucT_F | GAAAATCTATACTTCCAAGGATCCATGGCTCATGAGATTAAAAAGATCG |
| SvFucT_R | GGTGGTGGTGGTGCTCGAGTCAGAAAGAACTCACCCATTCAATAATC |
| ATCV-1-UG46DH_F | AATCTATACTTCCAAGGATCCATGAATTCCCAAGAGTACACACC |
| ATCV-1-UG46DH_R | GGTGGTGGTGGTGCTCGAGCTAGAATTTTGGAACCTGCAATGTTG |
| SvNMD_F | CAAGGGTGAAAATCTATACTTCCAAGGATCCATGGCCGAGGCTCCCTC |
| SvNMD_R | GGTGGTGGTGGTGCTCGAGTCACTCGAAGGAGGCAATTTCATTC |
| WsbK_F | GAAAATCTATACTTCCAAGGATCCATGAAGCGCATTCTTATTCTTGGTTGC |
| WsbK_R | GGTGGTGGTGGTGCTCGAGTTAAAAAATGTTCTTGGTCAGAATCGTATTAATG |
| QsFucSyn_F | GAAAATCTATACTTCCAAGGATCCATGGCAGAGGCTACGCAGC |
| QsFucSyn_R | GGTGGTGGTGGTGCTCGAGTTAGAACGGTGCCTCTTCTGTAC |
| QsFucT_H21A_F | GCCTTTCACGGGCAGGGAGCTATCGTCCCGCTTATCCAATTAAG |
| QsFucT_H21A_R | CTTAATTGGATAAGCGGGACGATAGCTCCCTGCCCGTGAAAGGC |
| QsFucT_D113N_F | GAAGAATCCAGTTAAGTGTTTGATTTATAATGCGAATCTTATCTGGGCTTTAG |
| QsFucT_D113N_R | CTAAAGCCCAGATAAGATTCGCATTATAAATCAAACACTTAACTGGATTCTTC |
| SvFucT_H20A_F | GTTTTTCCTTACCATGGACAGGGCGCCATGAACACAATGTTGCAATTCG |
| SvFucT_H20A_R | CGAATTGCAACATTGTGTTCATGGCGCCCTGTCCATGGTAAGGAAAAAC |
| SvFucT_D119N_F | GACAACAAGGTGCTGTTGGTATATAACGGGAACCTGCCTTGGG |
| SvFucT_D119N_R | CCCAAGGCAGGTTCCCGTTATATACCAACAGCACCTTGTTGTC |
| QsFucT_Q337A_F | GTAGTCACGTGGTGCCCGGCACTTAAAGTCCTTAGCCACCCTGC |
| QsFucT_Q337A_R | GCAGGGTGGCTAAGGACTTTAAGTGCCGGGCACCACGTGACTAC |
| QsFucT_T351A_F | TGCCGTAGGGTGCTTTATCGCGCATTGTGGTACTAACTCTATCTTCG |
| QsFucT_T351A_R | CGAAGATAGAGTTAGTACCACAATGCGCGATAAAGCACCCTACGGCA |
| QsFucT_H352A_F | CCGTAGGGTGCTTTATCACGGCTTGTGGTACTAACTCTATCTTCGAATC |
| QsFucT_H352A_R | GATTCGAAGATAGAGTTAGTACCACAAGCCGTGATAAAGCACCCTACGG |
| QsFucT_S357A_F | CTTTATCACGCATTGTGGTACTAACGCTATCTTCGAATCGGTTTGCTTCG |
| QsFucT_S357A_R | CGAAGCAAACCGATTCGAAGATAGCGTTAGTACCACAATGCGTGATAAAG |
| QsFucT_D376A_F | GTTGGTATGCCGCAATTCTGTGCCCAGATGCCTAATGCTTATTTCATG |
| QsFucT_D376A_R | CATGAAATAAGCATTAGGCATCTGGGCACAGAATTGCGGCATACCAAC |
| QsFucT_Q377A_F | GTATGCCGCAATTCTGTGACGCTATGCCTAATGCTTATTTCATGGAG |
| QsFucT_Q377A_R | CTCCATGAAATAAGCATTAGGCATAGCGTCACAGAATTGCGGCATAC |
| SvFucT_Q352A_F | GTATTGGCTTGGTGCCCCGCGTTGGACGTACTGAAACACGAG |
| SvFucT_Q352A_R | CTCGTGTTTCAGTACGTCCAACGCGGGGCACCAAGCCAATAC |
| SvFucT_T366A_F | GCATCGGCTGTTTTGTAGCACATTGCGGCTGGAATTCAATTAC |
| SvFucT_T366A_R | GTAATTGAATTCCAGCCGCAATGTGCTACAAAACAGCCGATGC |
| SvFucT_H367A_F | GCATCGGCTGTTTTGTAACAGCTTGCGGCTGGAATTCAATTAC |
| SvFucT_H367A_R | GTAATTGAATTCCAGCCGCAAGCTGTTACAAAACAGCCGATGC |
| SvFucT_S372A_F | GTAACACATTGCGGCTGGAATGCAATTACTGAAGCGTTAAGCTTCG |
| SvFucT_S372A_R | CGAAGCTTAACGCTTCAGTAATTGCATTCCAGCCGCAATGTGTTAC |
| SvFucT_D391A_F | GTCCGTGCCGCAGTTTCTTGCCCAGCCAGTTGACGCG |
| SvFucT_D391A_R | CGCGTCAACTGGCTGGGCAAGAAACTGCGGCACGGAC |
| SvFucT_Q392A_F | GTGCCGCAGTTTCTTGACGCCCCAGTTGACGCGCAC |
| SvFucT_Q392A_R | GTGCGCGTCAACTGGGGCGTCAAGAAACTGCGGCAC |

Figure S1: SDS-PAGE analysis of proteins used for enzymatic studies.

The protein standard ladder used is the PageRuler Plus Prestained Protein Ladder, 10 to 250 kDa (Thermo Scientific).

**Figure S2**: Multiple sequence alignment of glycosyltransferases involved in saponin C28 fucosylation.

The His-Asp catalytic dyad is denoted with a red asterisk. The plant secondary product glycosyltransferase (PSPG) motif is highlighted in yellow. Sequence alignment was performed using CLUSTAL Omega v1.2.4.^13^ NCBI protein accessions for SOAP6 and UGT74CD1 (“SoFucT”) are KNA07536.1 and WWM48152.1, respectively.


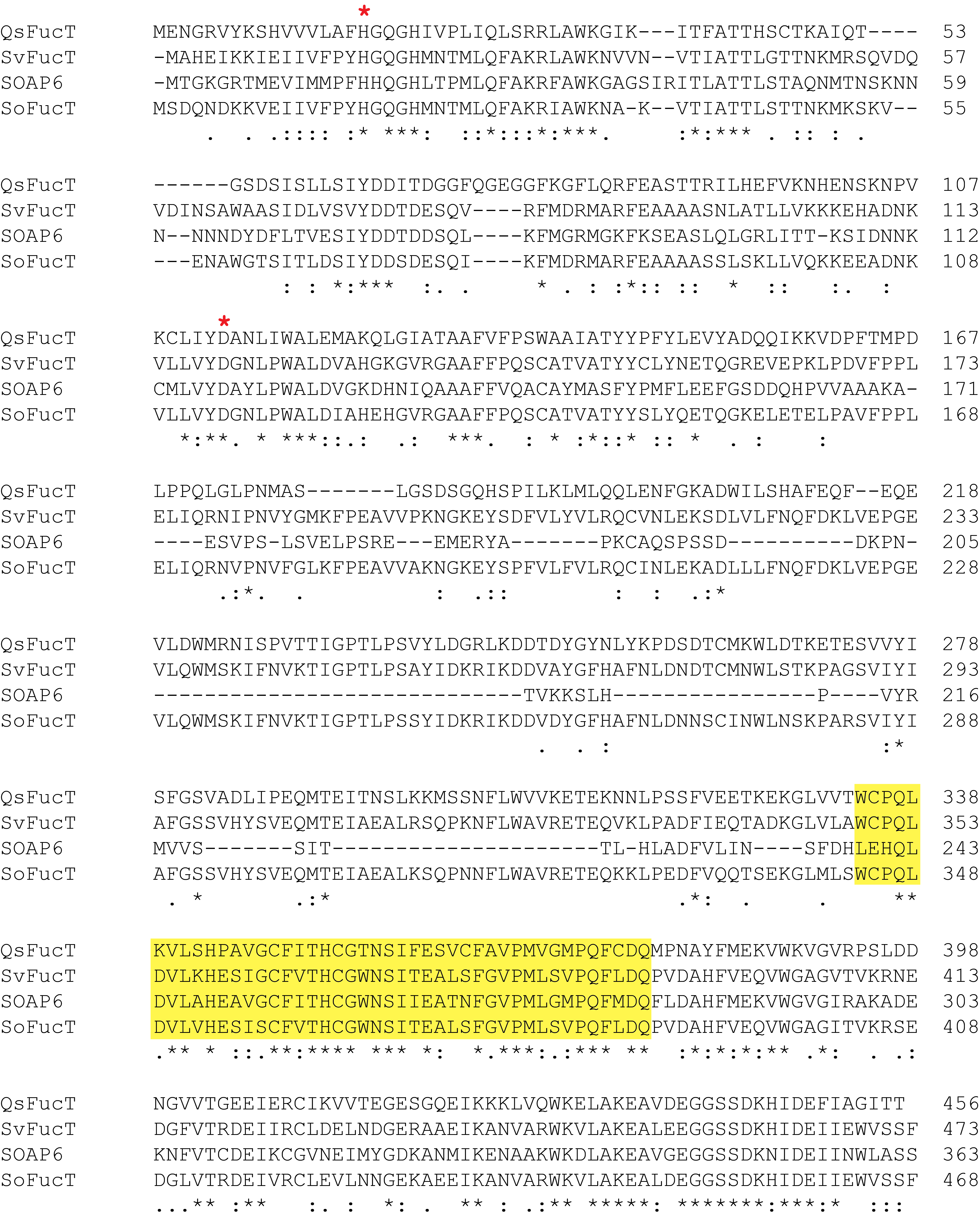


**Table S3:** Similarity-identity matrix of glycosyltransferases involved in saponin C28 fucosylation.

The multiple sequence alignment used to generate these data can be found in **Figure S2**. Percent identity is shown in blue shaded boxes (with underlined numbers) while percent similarity is shown in green shaded boxes.

|  | **QsFucT** | **SvFucT** | **SOAP6** | **SoFucT** |
| --- | --- | --- | --- | --- |
| **QsFucT** |  | 53.3 | 44.2 | 53.3 |
| **SvFucT** | 42.9 |  | 54.9 | 89.3 |
| **SOAP6** | 37.4 | 44.0 |  | 54.7 |
| **SoFucT** | 43.1 | 83.7 | 45.4 |  |

**Figure S3:** LC-MS purity analysis of TriX-QA from QS-21 hydrolysis.

TriX-QA obtained by base hydrolysis of QS-21 resulted in a product that is chemically indistinguishable from TriX-QA heterologously produced in planta. (A) RP-HPLC chromatograms of TriX-QA from QS-21 hydrolysis and from in planta production. (B) ESI-MS spectra of TriX-QA from QS-21 hydrolysis and from in planta production.


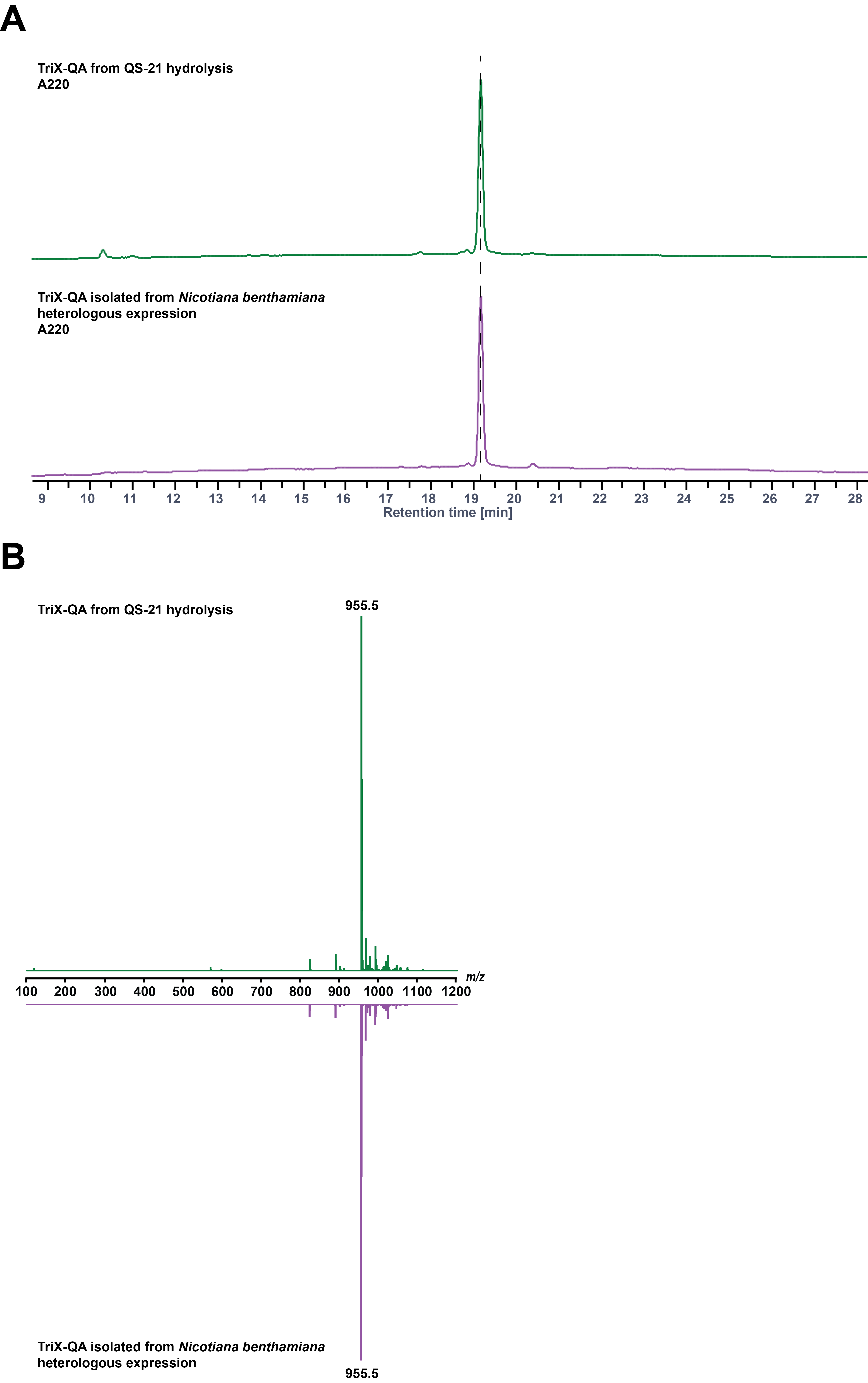


Figure S4: In vitro enzymatic synthesis of UDP-4-keto-6-deoxy-D-Glc from UDP-D-Glc.

(A) HPLC chromatograms of UDP-D-Glc lacking other reaction components (top panel) and its full conversion into UDP-4-keto-6-deoxy-D-Glc upon enzyme treatment (bottom panel). (B) ESI-MS spectra of the new product at Rt = 21.0-23.5 min. UDP-4-keto-6-deoxy-D-Glc exists in rapid equilibrium with its gem-diol hydrate form. (C) Structures of UDP-4-keto-6-deoxy-D-Glc and its gem-diol hydrate shown as the deprotonated forms expected in negative ESI-MS.


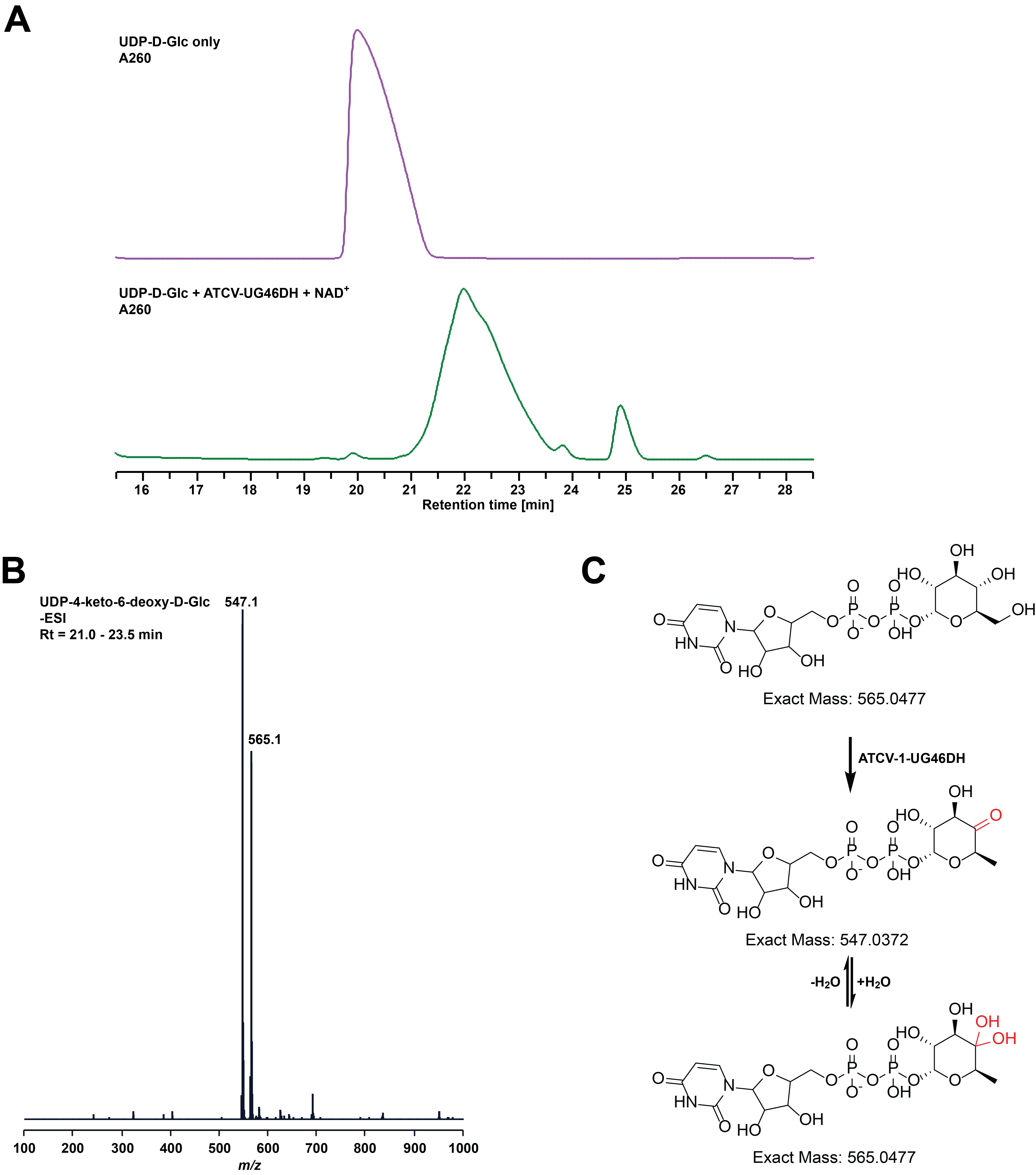


**Figure S5:** LC-MS purity analysis of enzymatically synthesized, purified UDP-4-keto-6-deoxy-D-Glc.

UDP-4-keto-6-deoxy-Glc elutes over several minutes as a broad, smeared peak, likely due to rapid equilibrium between the keto- and geminal diol hydrate forms. Note that MS chromatograms begin at 5 minutes due to solvent delay.


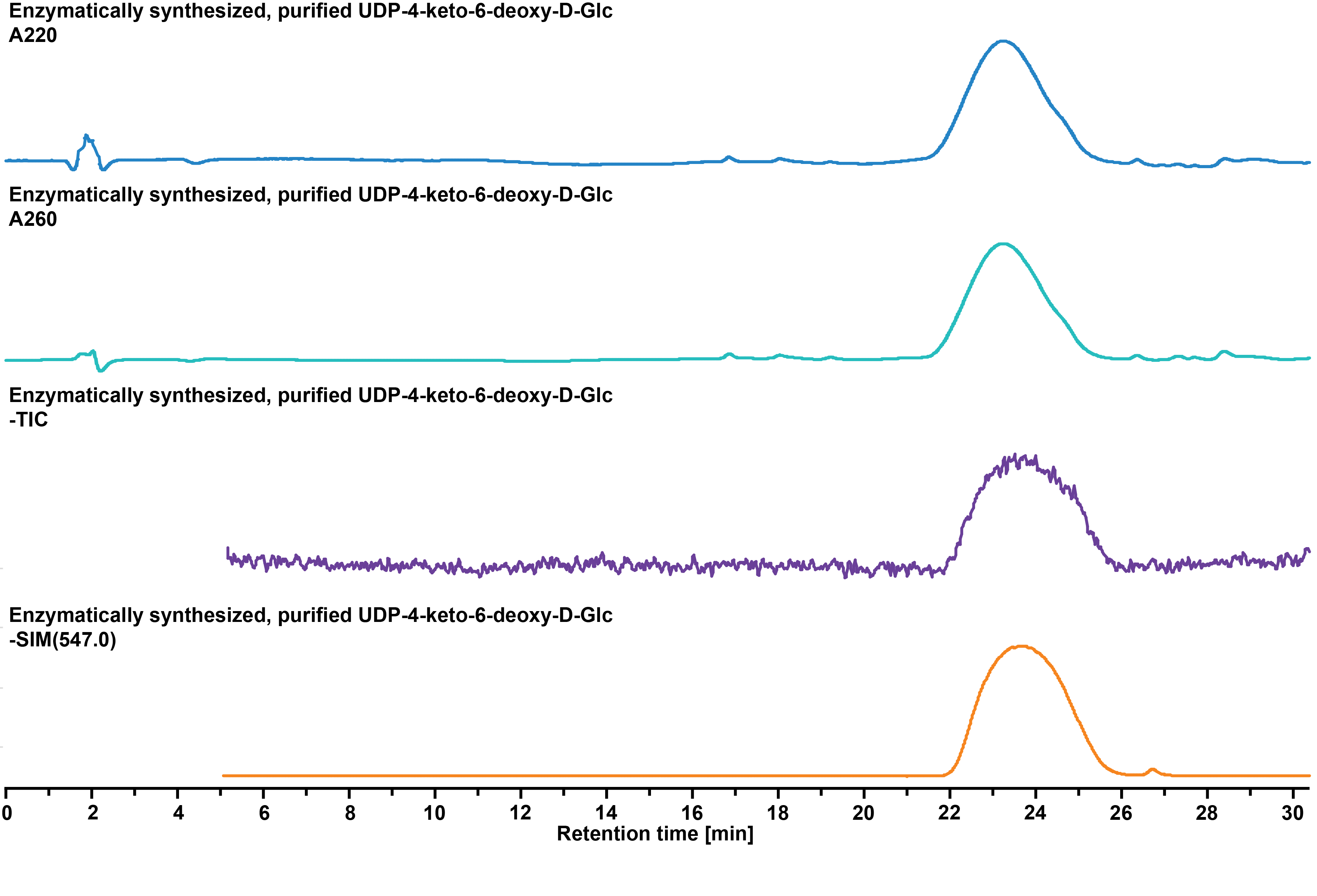


**Figure S6:** In vitro enzymatic synthesis of UDP-D-Fuc from UDP-D-Glc.

(A) HPLC chromatograms of in vitro sugar transformations including UDP-4-keto-6-deoxy-D-Glc produced by ATCV-1-UG46DH (top panel) and UDP-4-keto-6-deoxy-D-Glc treated with either SvNMD or wskB (middle panels). The new product peak at Rt = 19.8 min has an identical retention time as a standard of UDP-D-Fuc (bottom panel). (B) Comparison of ESI-MS spectra of the new product peak with that of UDP-D-Fuc standard. (C) Structure of UDP-D-Fuc shown as the deprotonated form expected in negative ESI-MS.


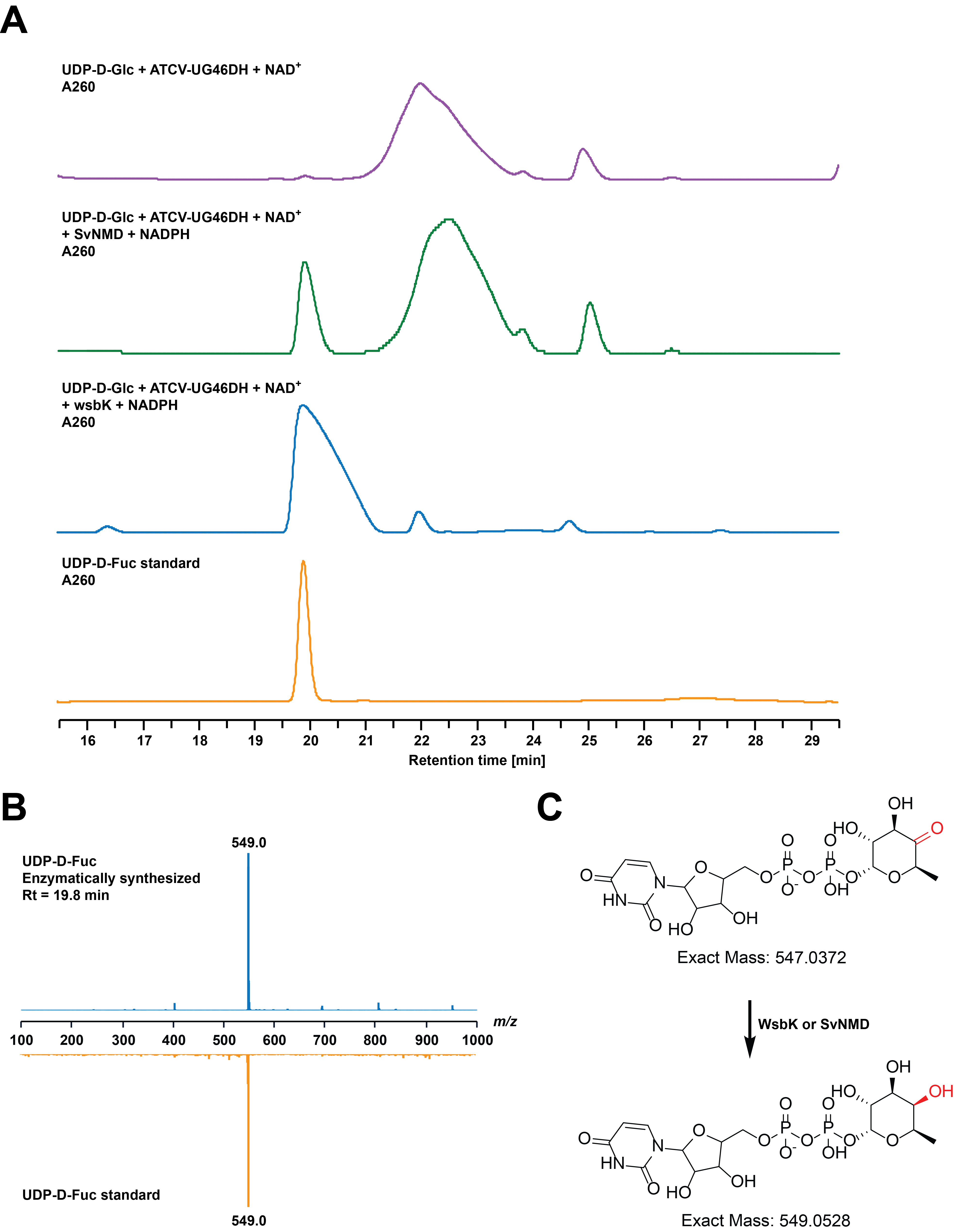


**Figure S7:** LC-MS purity analysis of enzymatically synthesized, purified UDP-D-Fuc.

UDP-D-Fuc synthesized using a combination of ATCV-1-UG46DH and wskB is indistinguishable from a UDP-D-Fuc standard. Note that mass spectrometry chromatograms (negative total ion chromatogram [-TIC] and single ion monitoring [SIM]) do not align with absorbance chromatograms due to delay between absorbance and MS detection.


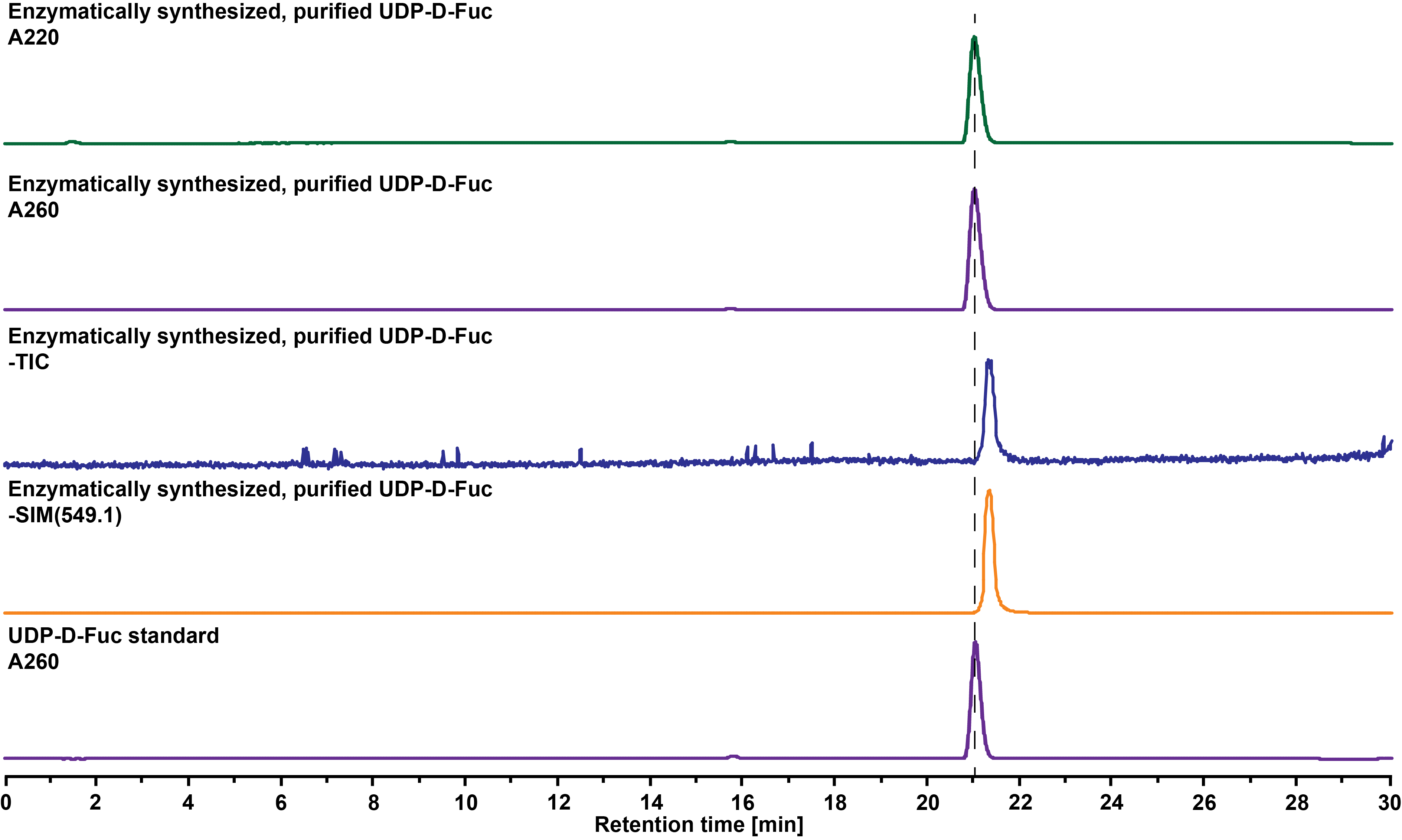


**Figure S8:** Mass spectrometry analysis of QsFucT and SvFucT reaction products using UDP-4-keto-6-deoxy-D-Glc or UDP-D-Fuc.

(A) Reaction scheme for glycosylation assays by QsFucT or SvFucT using either UDP-4-keto-6-deoxy-D-Glc (with or without QsFucSyn) or UDP-D-Fuc. Species shown are in the deprotonated form observed by ESI-MS. (B) Mass spectra of QsFucT reaction products shown in chromatograms of **Figure 2**. (C) Mass spectra of SvFucT reaction products shown in chromatograms of **Figure 2**. UDP-4K6DG, UDP-4-keto-6-deoxy-D-Glc.

**
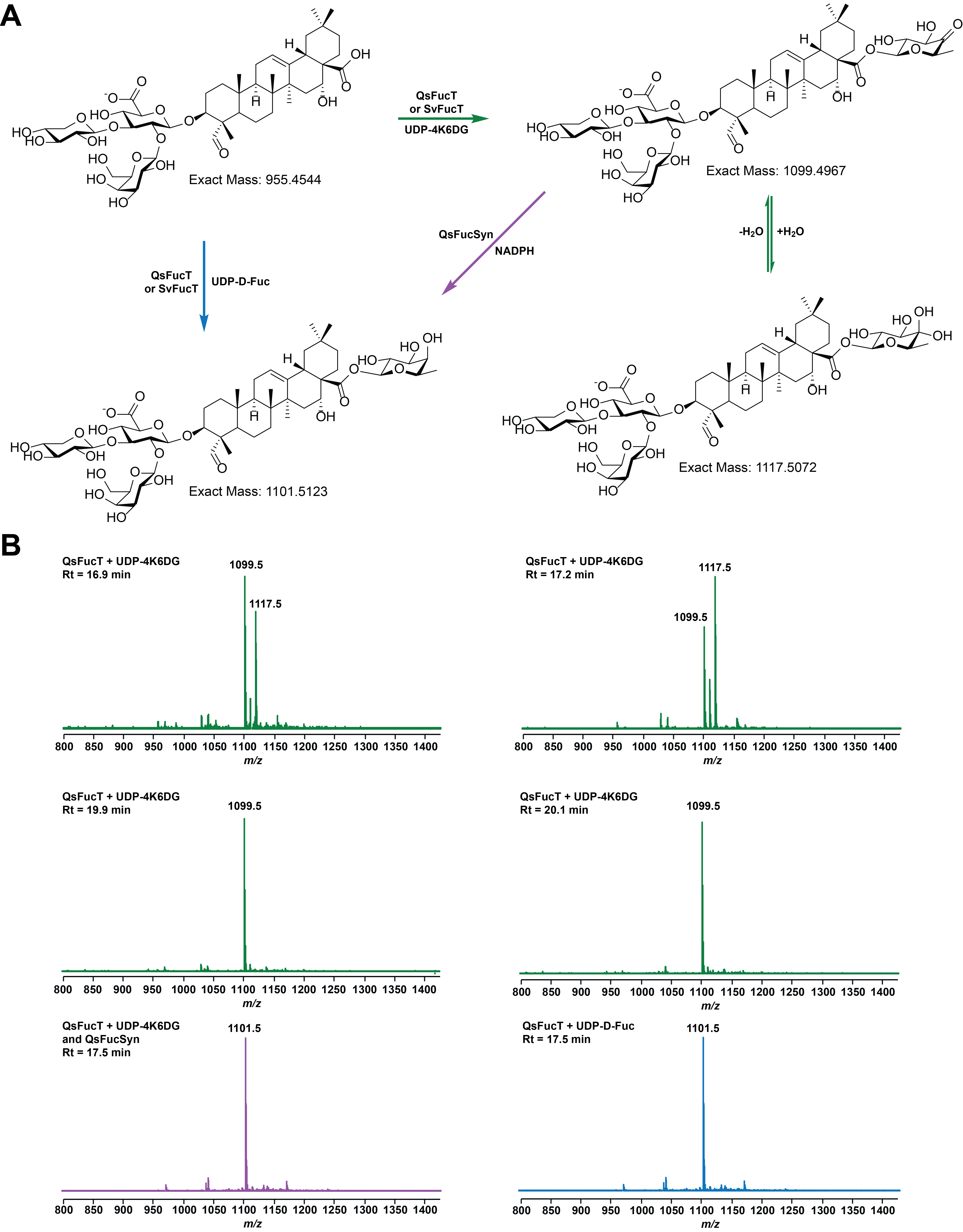
**

**Figure S8** (continued)

**
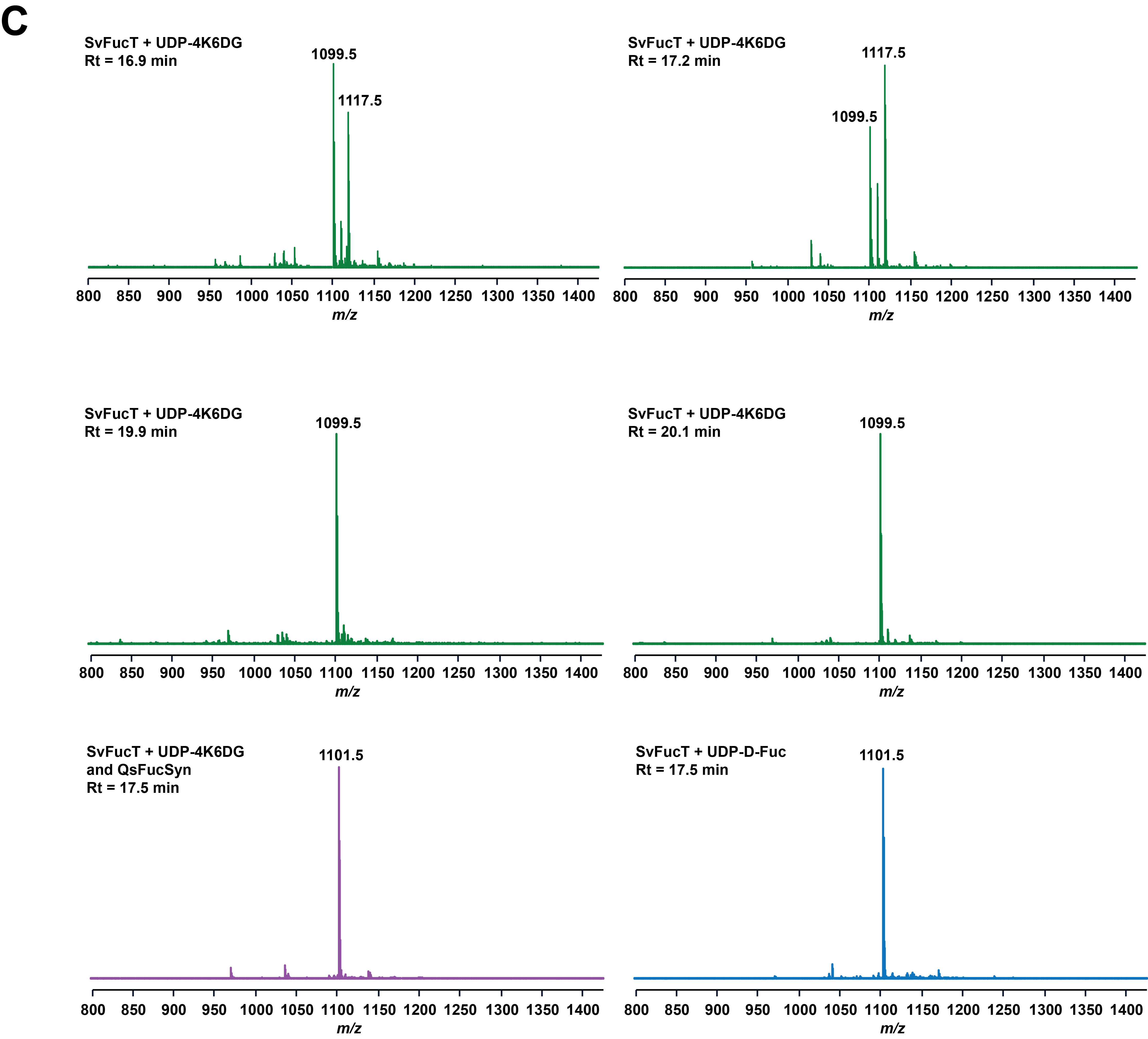
**

**Figure S9:** Kinetic assays of QsFucT with TriX-QA, UDP-4-keto-6-deoxy-D-Glc, UDP-D-Fuc.

Shown are luminescence readings after a 10 minute glycosylation reaction at various reactant concentrations. (A) Kinetics curve for TriX-QA saponin acceptor. (B) Kinetics curve for UDP-4-keto-6-deoxy-D-Glc sugar donor. (C) Kinetics curve for UDP-D-Fuc sugar donor. Luminescence data were fit to the Michaelis-Menten model using OriginPro 2022b with the Orthogonal Distance Regression iteration algorithm. V_max_ in luminescence was converted to μmol UDP generated using a standard curve generated from UDP.


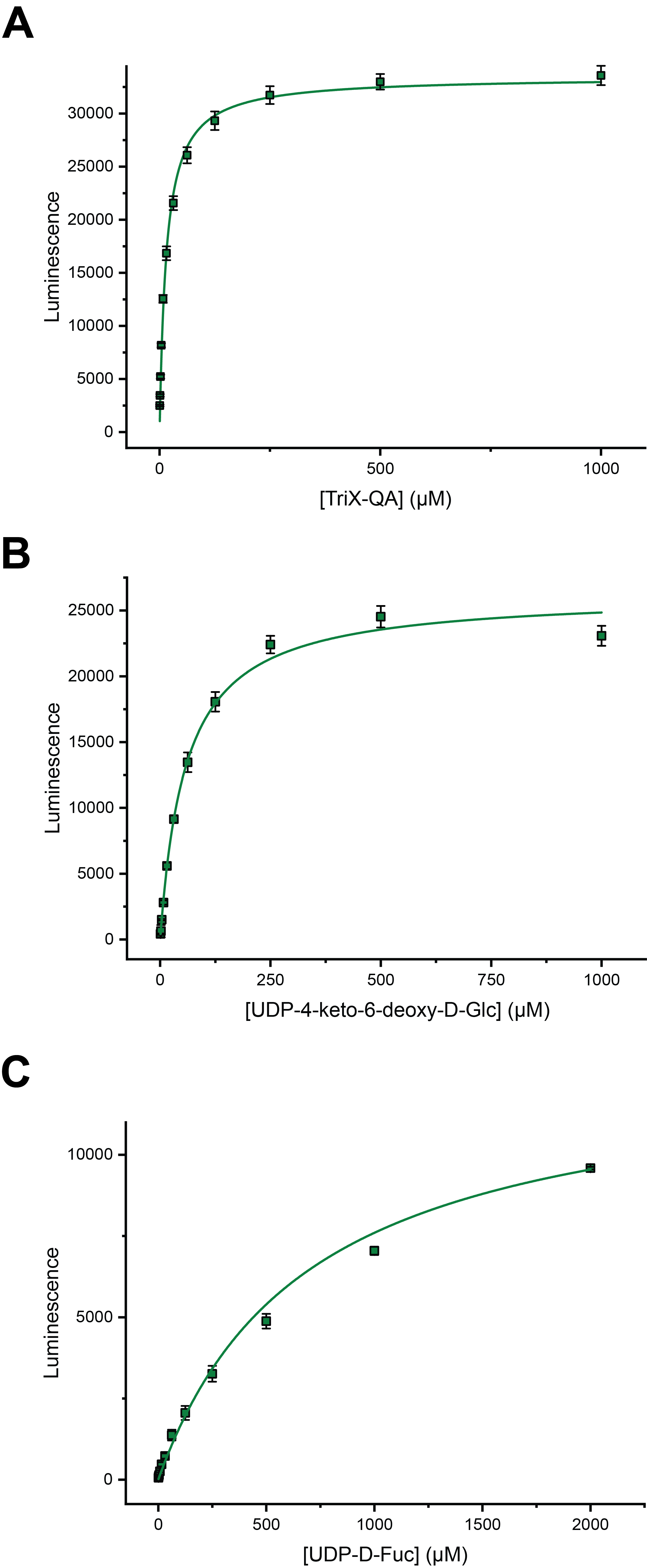


**Figure S10:** Kinetic assays of SvFucT with TriX-QA, UDP-4-keto-6-deoxy-D-Glc, UDP-D-Fuc.

Shown are luminescence readings after a 10 minute glycosylation reaction at various reactant concentrations. (A) Kinetics curve for TriX-QA saponin acceptor. (B) Kinetics curve for UDP-4-keto-6-deoxy-D-Glc sugar donor. (C) Kinetics curve for UDP-D-Fuc sugar donor. Luminescence data were fit to the Michaelis-Menten model using OriginPro 2022b with the Orthogonal Distance Regression iteration algorithm. V_max_ in luminescence was converted to μmol UDP generated using a standard curve generated from UDP.


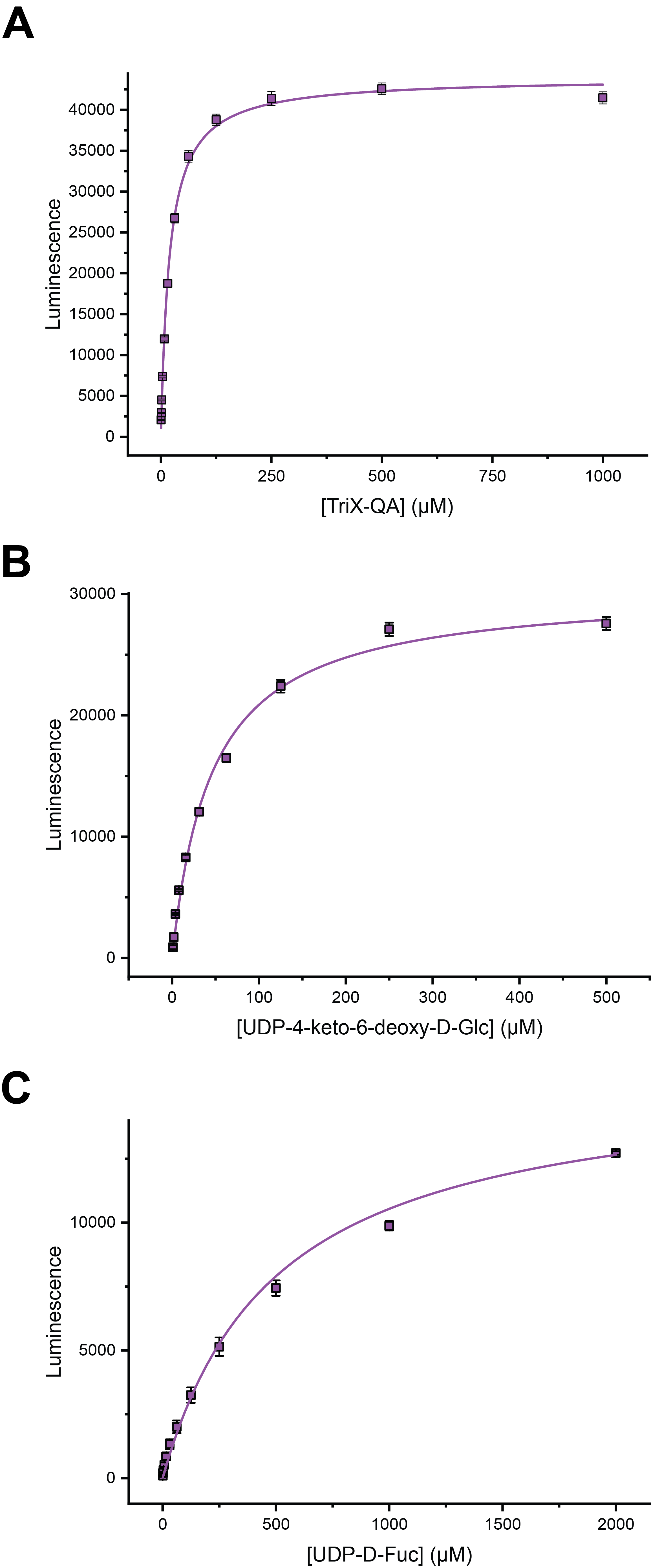


**Figure S11:** Mass spectra for products observed in Main Text Figure 3.

(A) Expected structure and product mass spectra for the fucosylation of quillaic acid [QA]. (B) Expected structure and product mass spectra for the fucosylation of calenduloside E [CE]. (C) Structure of starting material MeGlcA-gypsogenin [MeGlcA-Gyp] and mass spectra observed for its spuriously reduced, but not fucosylated, product. (D) Expected structure and product mass spectra for the fucosylation of MeGlcA-Gyp, which appears solely as an unexpected species that is both fucosylated and formally reduced. QA, MeGlcA-Gyp, and their products appeared as formate adducts in mass spectra.


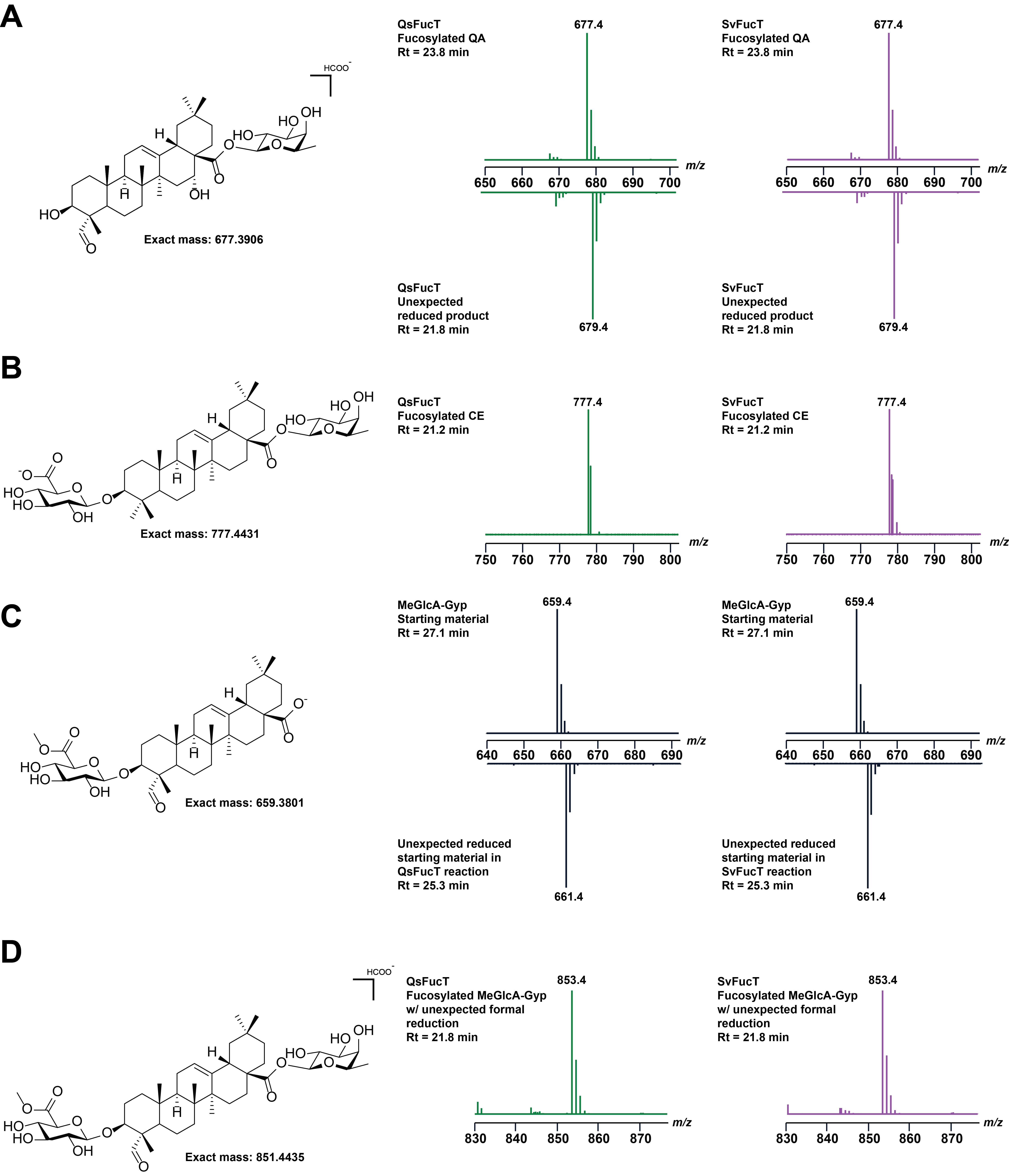


**Figure S12:** In vitro reduction assay by FucSyn on various terpene/saponin acceptors.

For each acceptor tested, the A220 HPLC chromatogram is provided and, if a product was formed, the ESI mass spectrum of the product peak. Acceptors tested but not included in the Main Text are (A) oleanolic acid, (B) echinocystic acid, (C) hederagenin, (D) calenduloside E, (E) gyspogenin, and (F) MeGlcA-gypsogenin. For the gypsogenin reaction, the dashed line shows that the observed product has an identical retention time to a hederagenin standard.


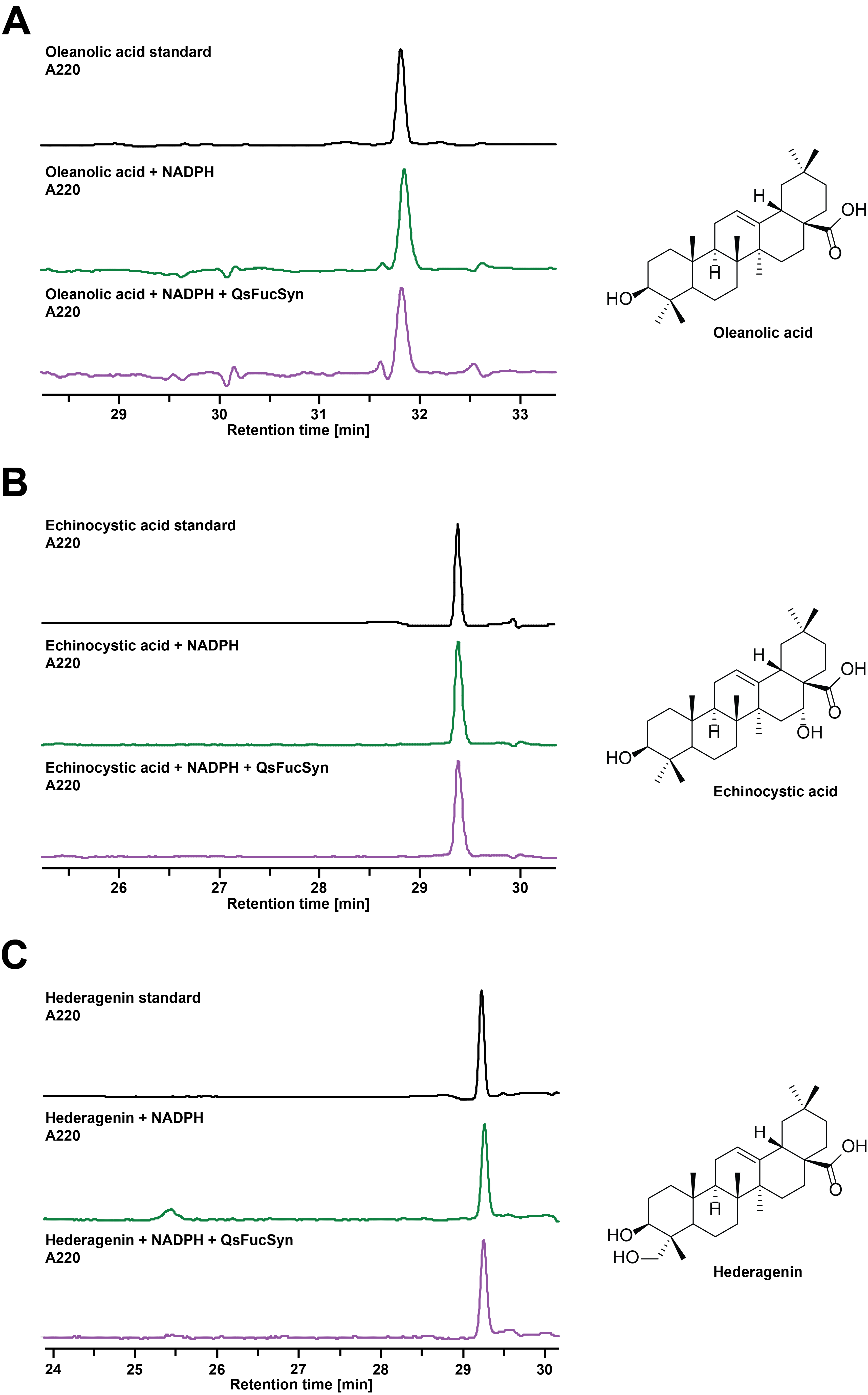


**Figure S12** (continued)


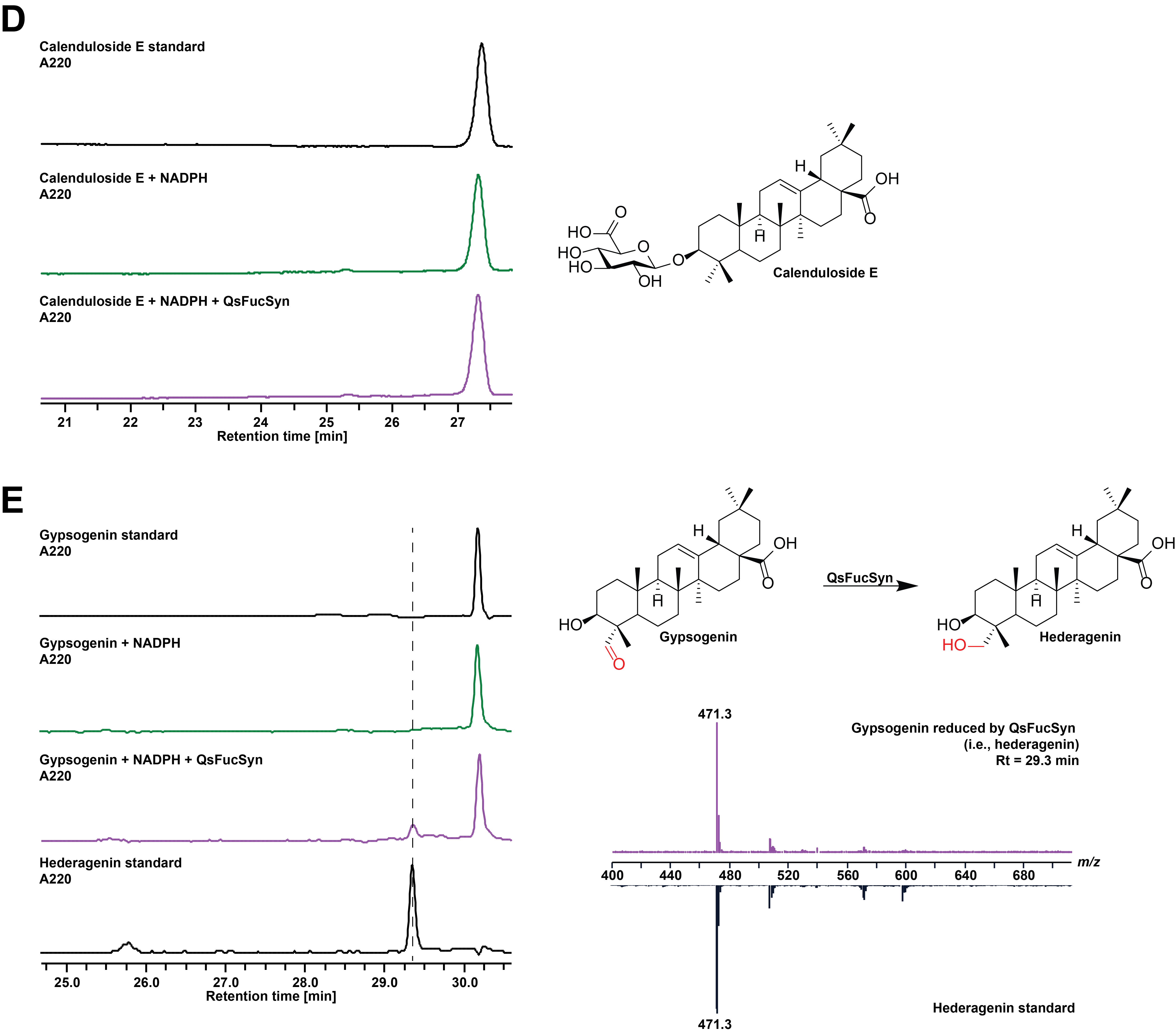


**Figure S13:** In vitro glycosylation of TriX-QA with UDP-D-Glc by QsFucT.

(A) The reaction between TriX-QA and UDP-D-Glc catalyzed by QsFucT. The starting material and product are depicted in the deprotonated form anticipated to be observed in negative mode ESI-MS. (B) A220 HPLC and negative mode TIC chromatograms of the glycosylation reaction. (C) ESI-MS of the product peak at Rt = 15.0 min.


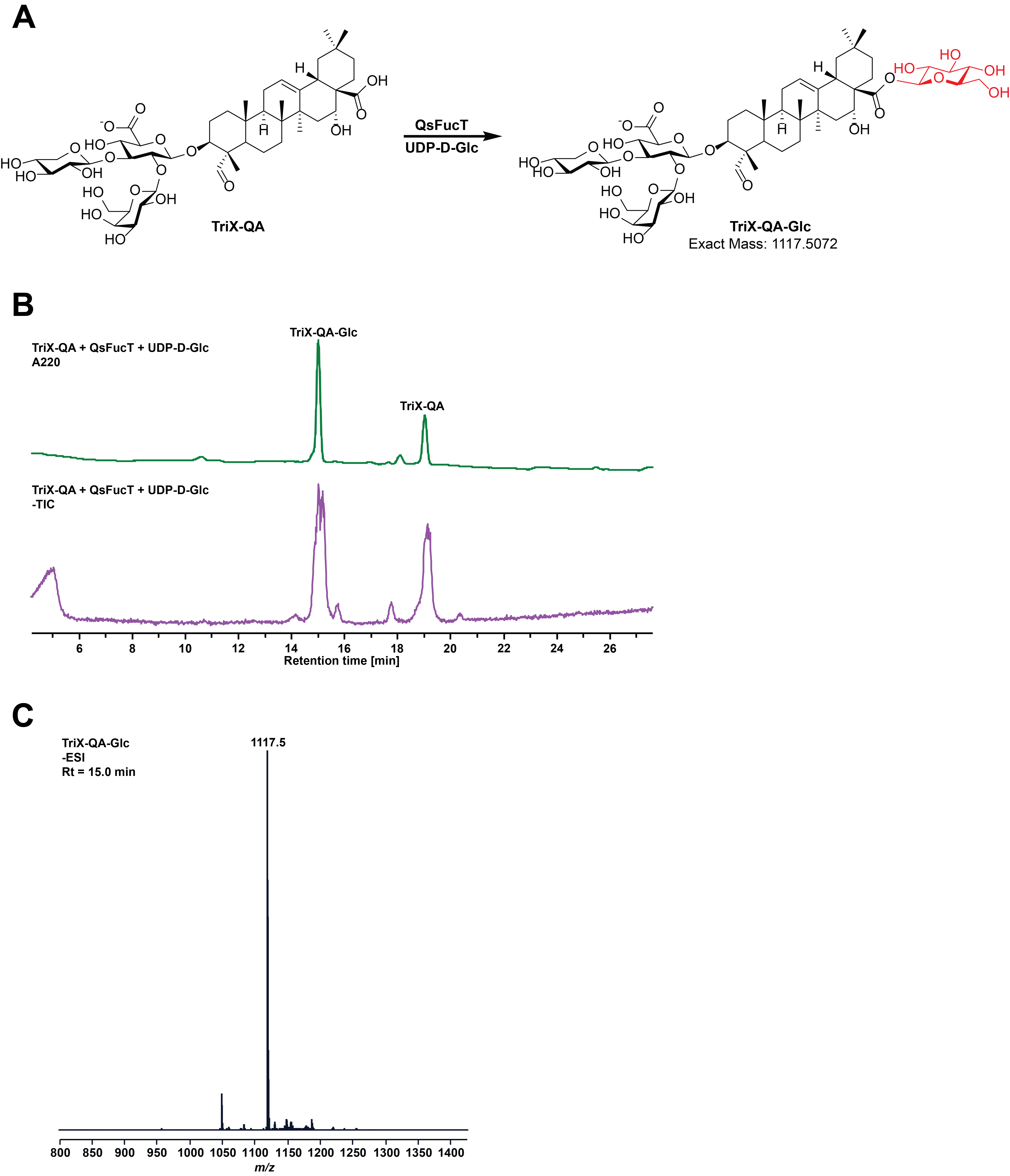


**Figure S14:** In vitro glycosylation of TriX-QA with UDP-D-Gal by QsFucT.

(A) The reaction between TriX-QA and UDP-D-Gal catalyzed by QsFucT. The starting material and product are depicted in the deprotonated form anticipated to be observed in negative mode ESI-MS. (B) A220 HPLC and negative mode TIC chromatograms of the glycosylation reaction. (C) ESI-MS of the product peak at Rt = 14.8 min.


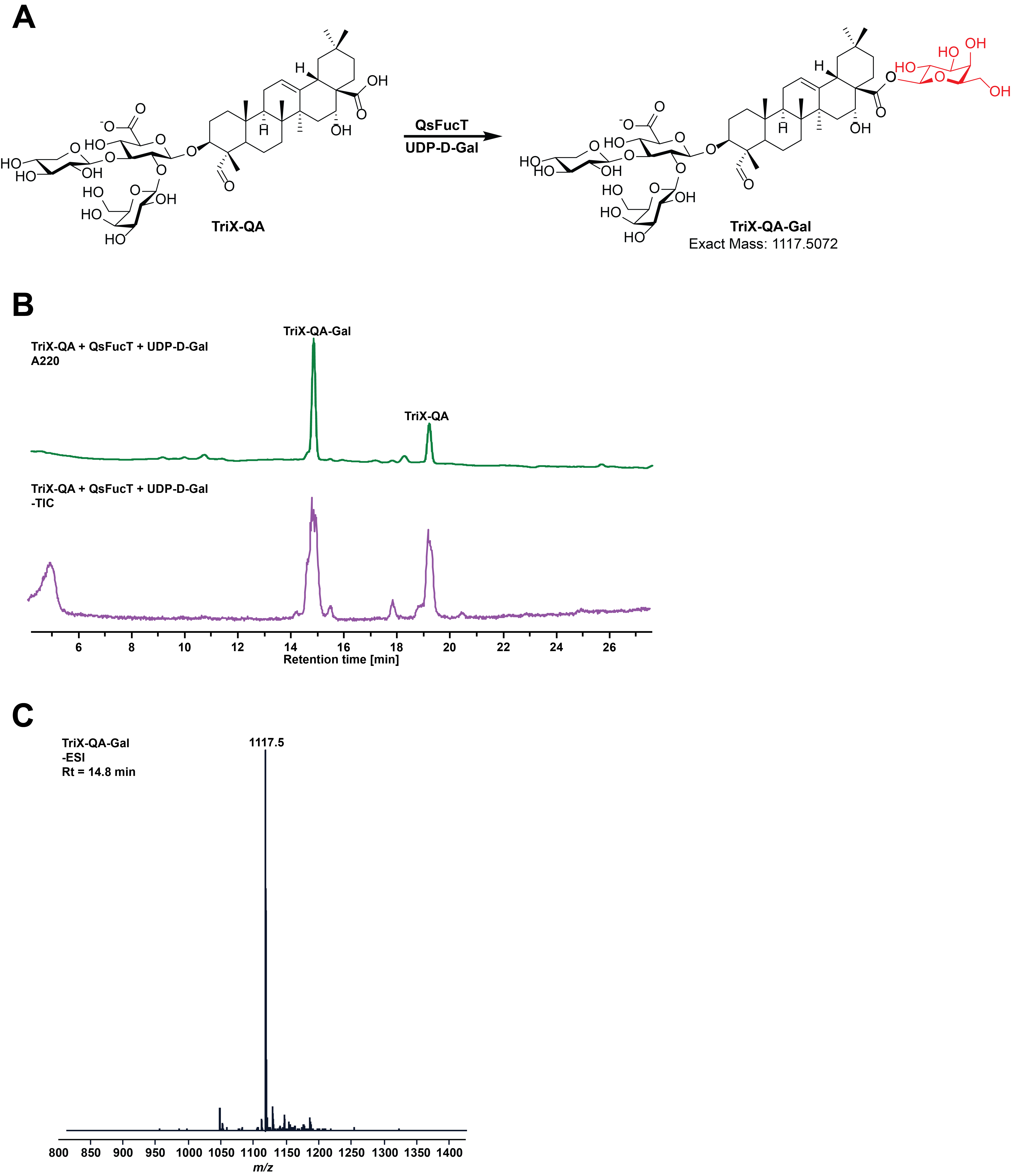


**Figure S15:** In vitro glycosylation of TriX-QA with UDP-L-Rha by QsFucT.

(A) The non-reaction between TriX-QA and UDP-L-Rha catalyzed by QsFucT. The starting material and product are depicted in the deprotonated form anticipated to be observed in negative mode ESI-MS. (B) A220 HPLC and negative mode TIC chromatograms of the glycosylation reaction. No significant products were observed.


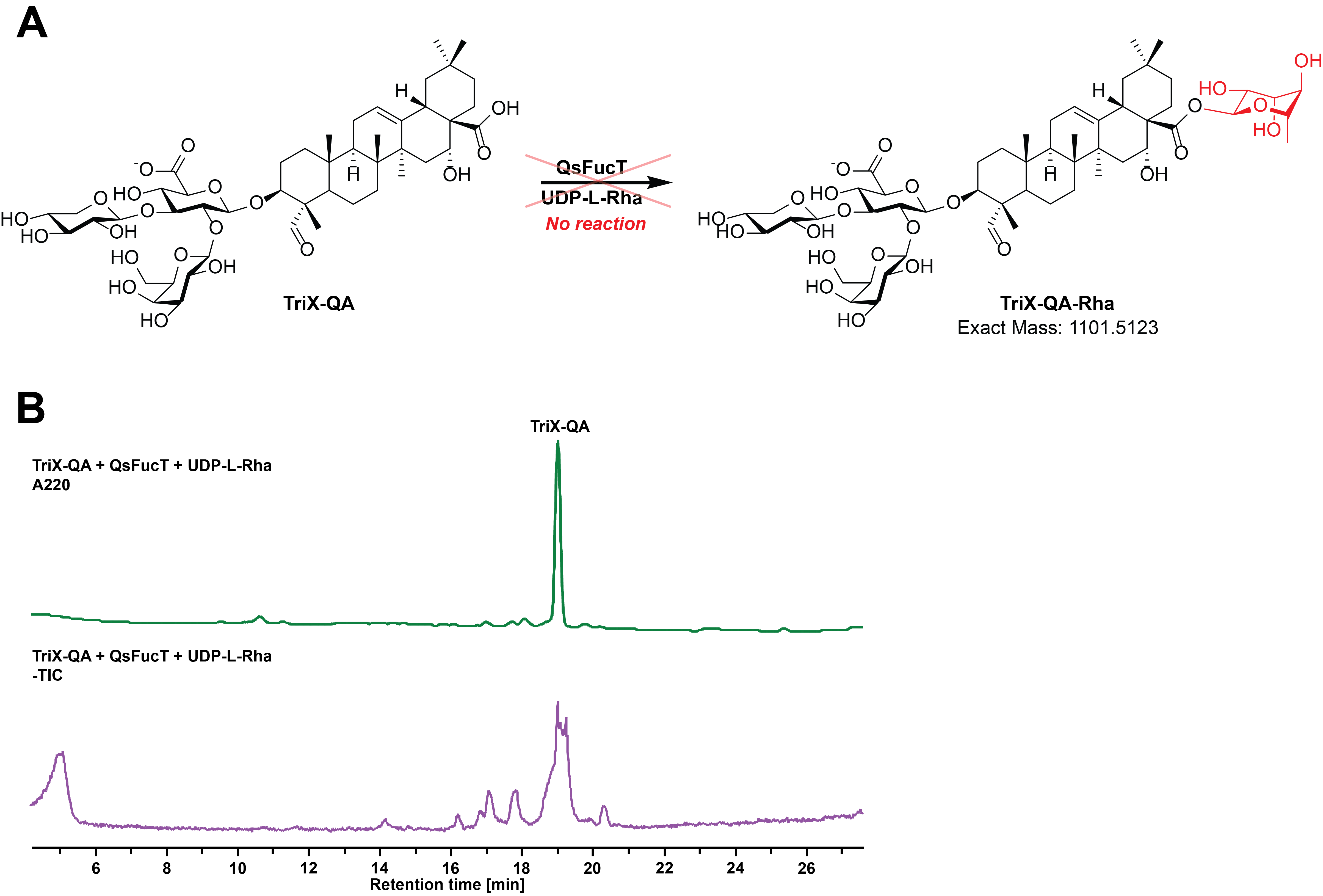


Figure S16: In vitro glycosylation of TriX-QA with UDP-D-Xyl by QsFucT.

(A) The reaction between TriX-QA and UDP-D-Xyl catalyzed by QsFucT. The starting material and product are depicted in the deprotonated form anticipated to be observed in negative mode ESI-MS. (B) A220 HPLC and negative mode TIC chromatograms of the glycosylation reaction. (C) ESI-MS of the product peak at Rt = 17.0 min.


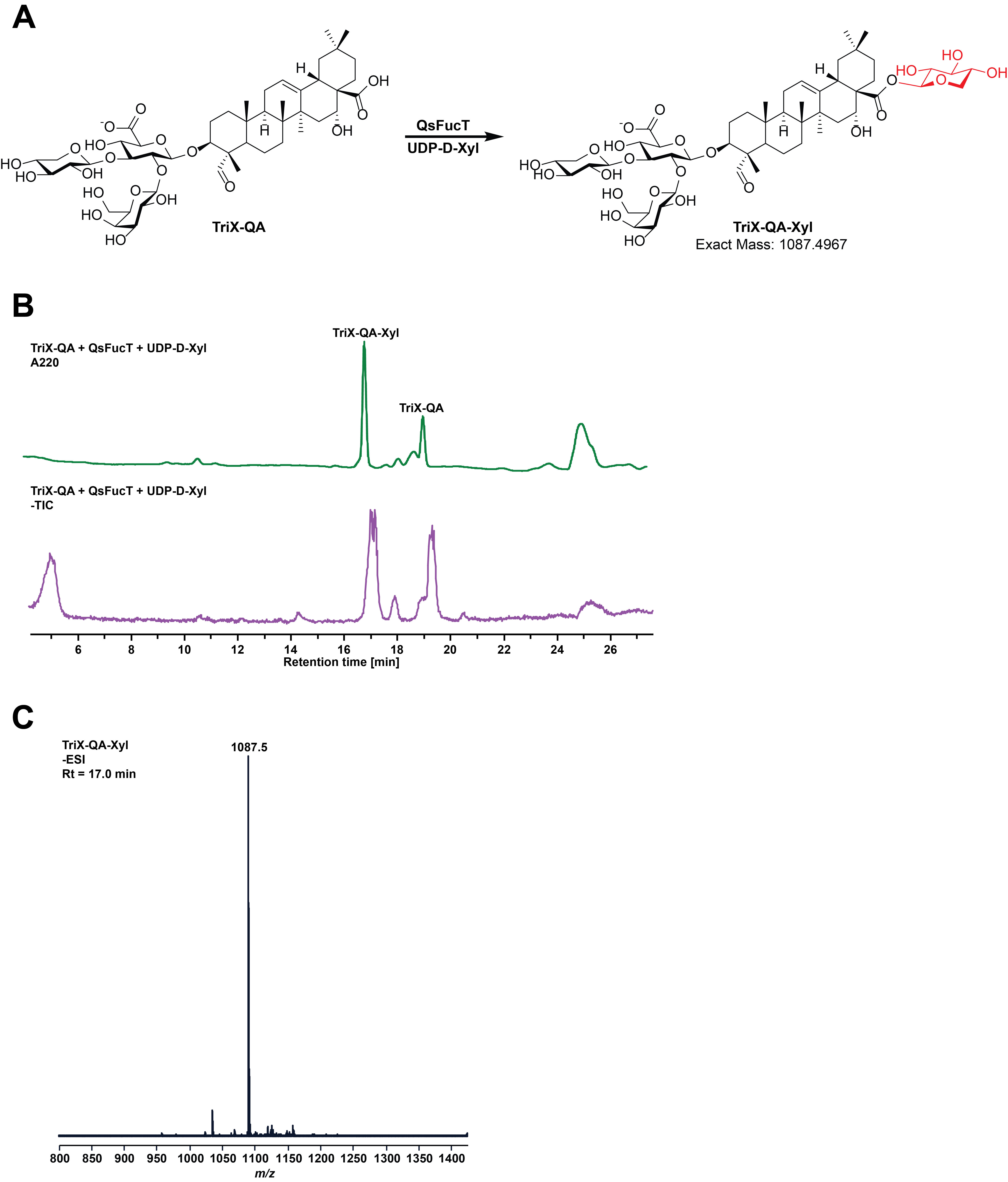


**Figure S17:** In vitro glycosylation of TriX-QA with UDP-L-Ara*p* by QsFucT.

(A) The reaction between TriX-QA and UDP-L-Ara*p* catalyzed by QsFucT. The starting material and product are depicted in the deprotonated form anticipated to be observed in negative mode ESI-MS. (B) A220 HPLC and negative mode TIC chromatograms of the glycosylation reaction. (C) ESI-MS of the product peak at Rt = 16.5 min.

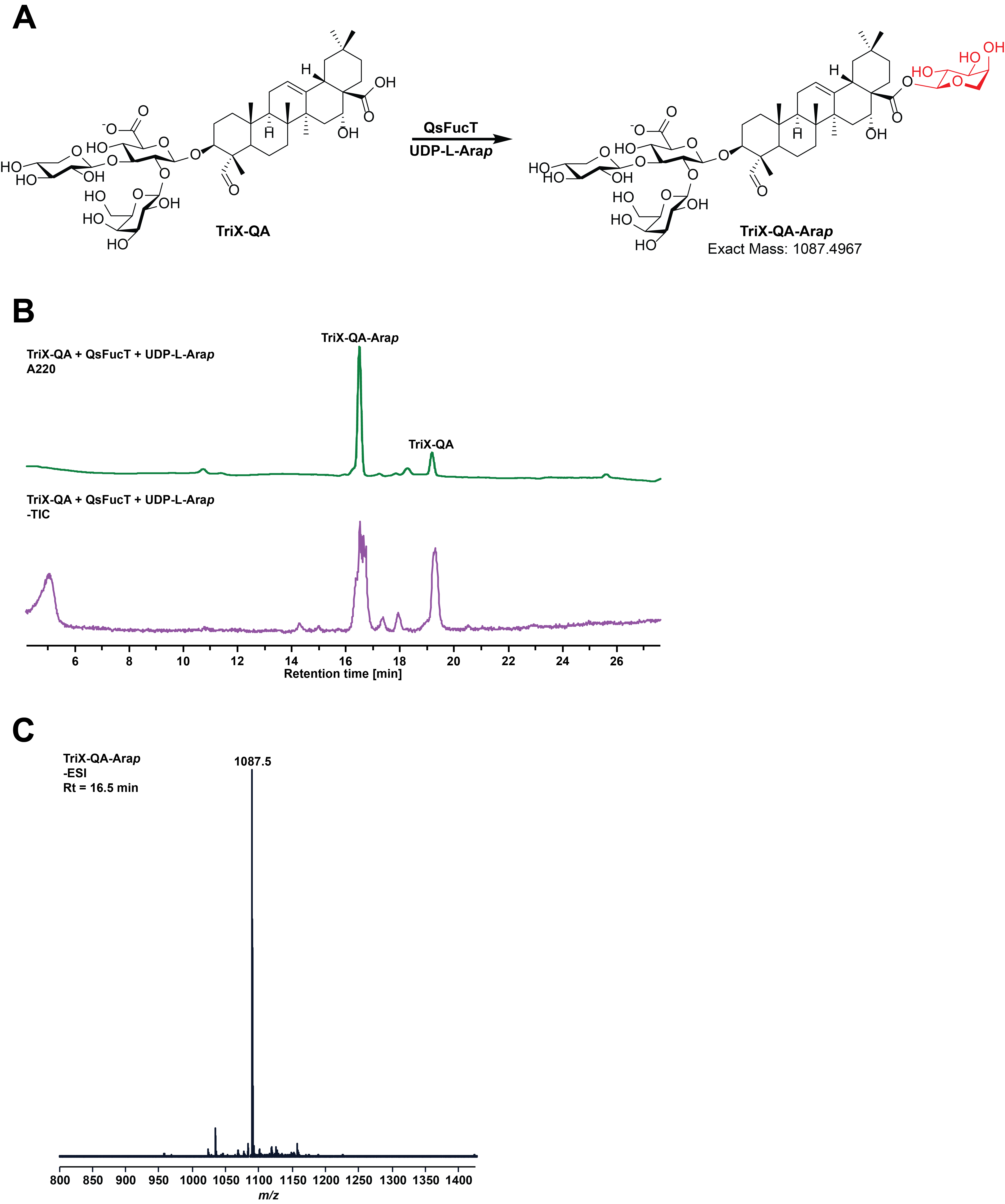


**Figure S18:** In vitro glycosylation of TriX-QA with UDP-L-Ara*f* by QsFucT.

(A) The reaction between TriX-QA and UDP-L-Ara*f* catalyzed by QsFucT. The starting material and product are depicted in the deprotonated form anticipated to be observed in negative mode ESI-MS. (B) A220 HPLC and negative mode TIC chromatograms of the glycosylation reaction. (C) ESI-MS of the product peak at Rt = 16.1 min.


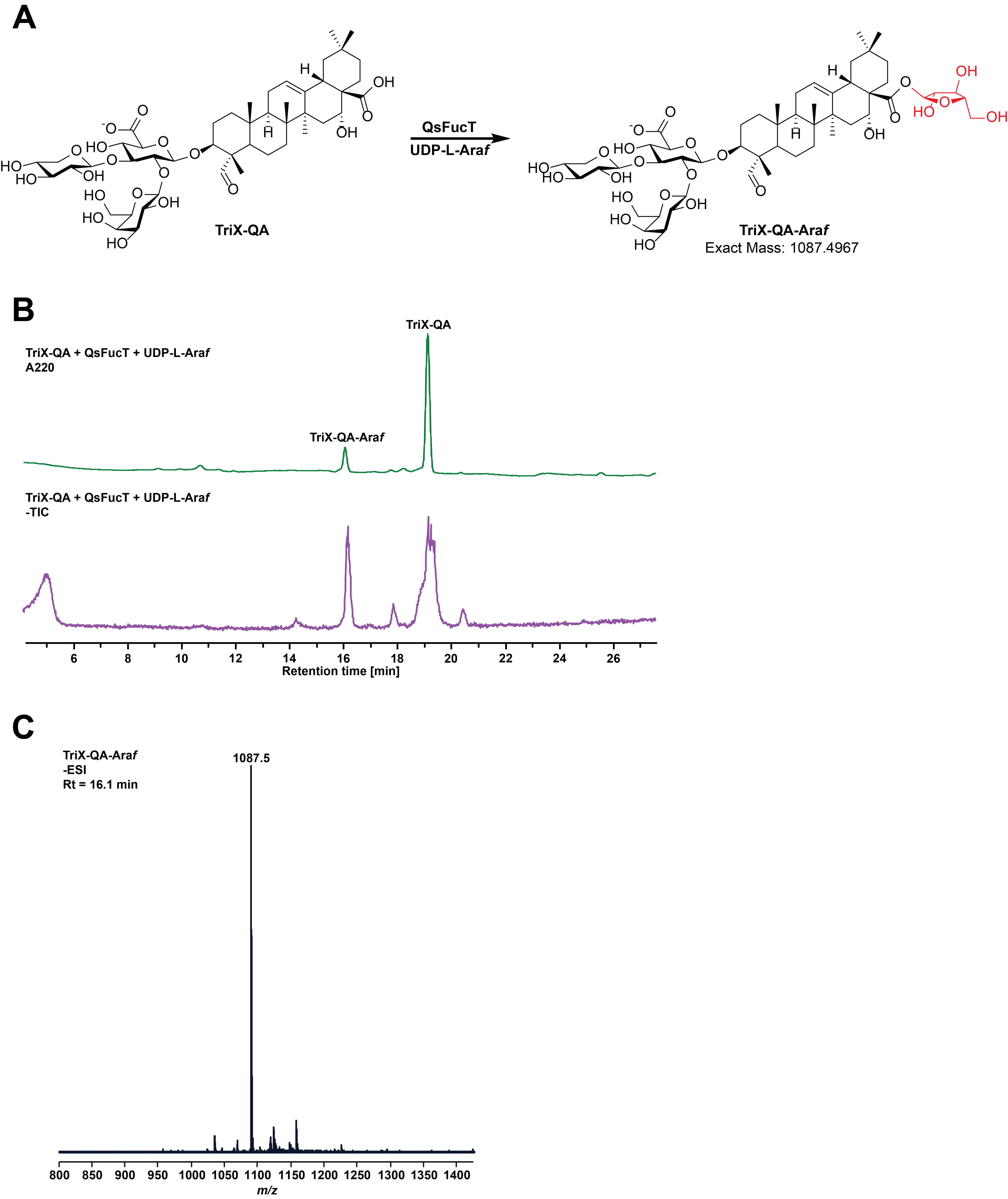


**Figure S19:** In vitro glycosylation of TriX-QA with UDP-D-GlcA by QsFucT.

(A) The non-reaction between TriX-QA and UDP-D-GlcA catalyzed by QsFucT. The starting material and product are depicted in the deprotonated form anticipated to be observed in negative mode ESI-MS. (B) A220 HPLC and negative mode TIC chromatograms of the glycosylation reaction. No significant products were observed.


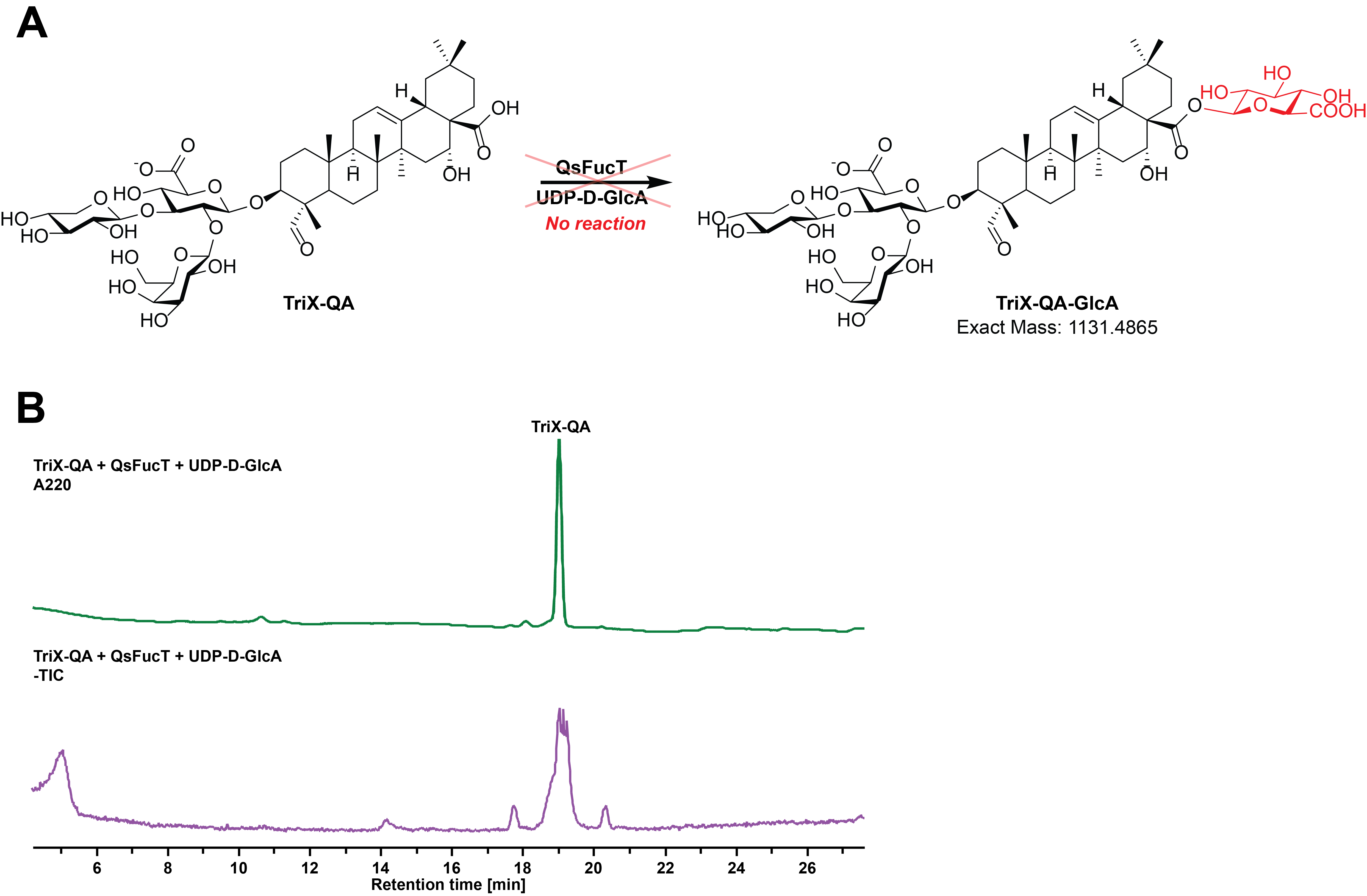


**Figure S20:** In vitro glycosylation of TriX-QA with UDP-D-GalA by QsFucT.

(A) The non-reaction between TriX-QA and UDP-D-GalA catalyzed by QsFucT. The starting material and product are depicted in the deprotonated form anticipated to be observed in negative mode ESI-MS. (B) A220 HPLC and negative mode TIC chromatograms of the glycosylation reaction. No significant products were observed.


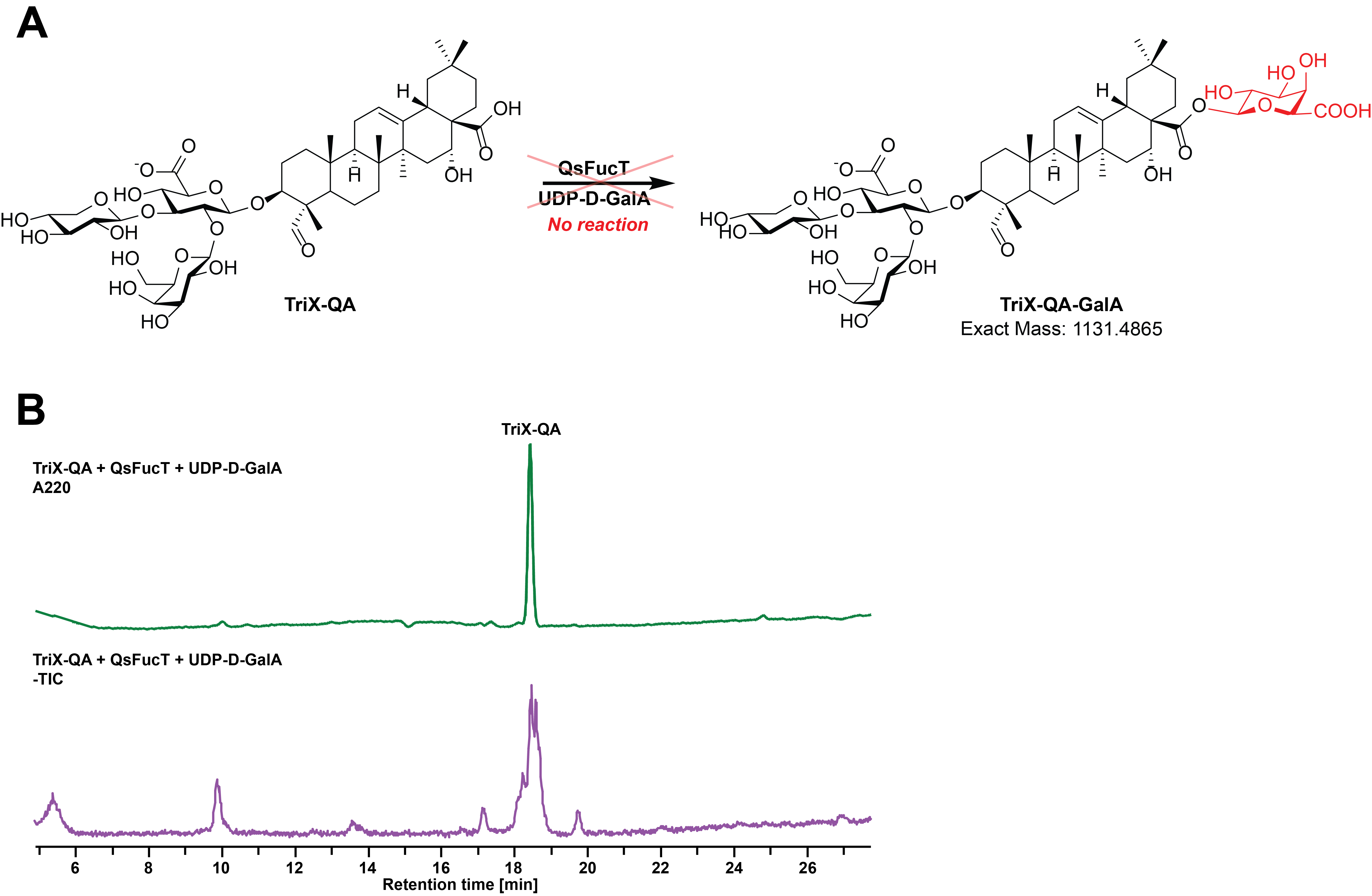


**Figure S21:** In vitro glycosylation of TriX-QA with UDP-D-GlcNAc by QsFucT.

(A) The non-reaction between TriX-QA and UDP-D-GlcNAc catalyzed by QsFucT. The starting material and product are depicted in the deprotonated form anticipated to be observed in negative mode ESI-MS. (B) A220 HPLC and negative mode TIC chromatograms of the glycosylation reaction. No significant products were observed.

**
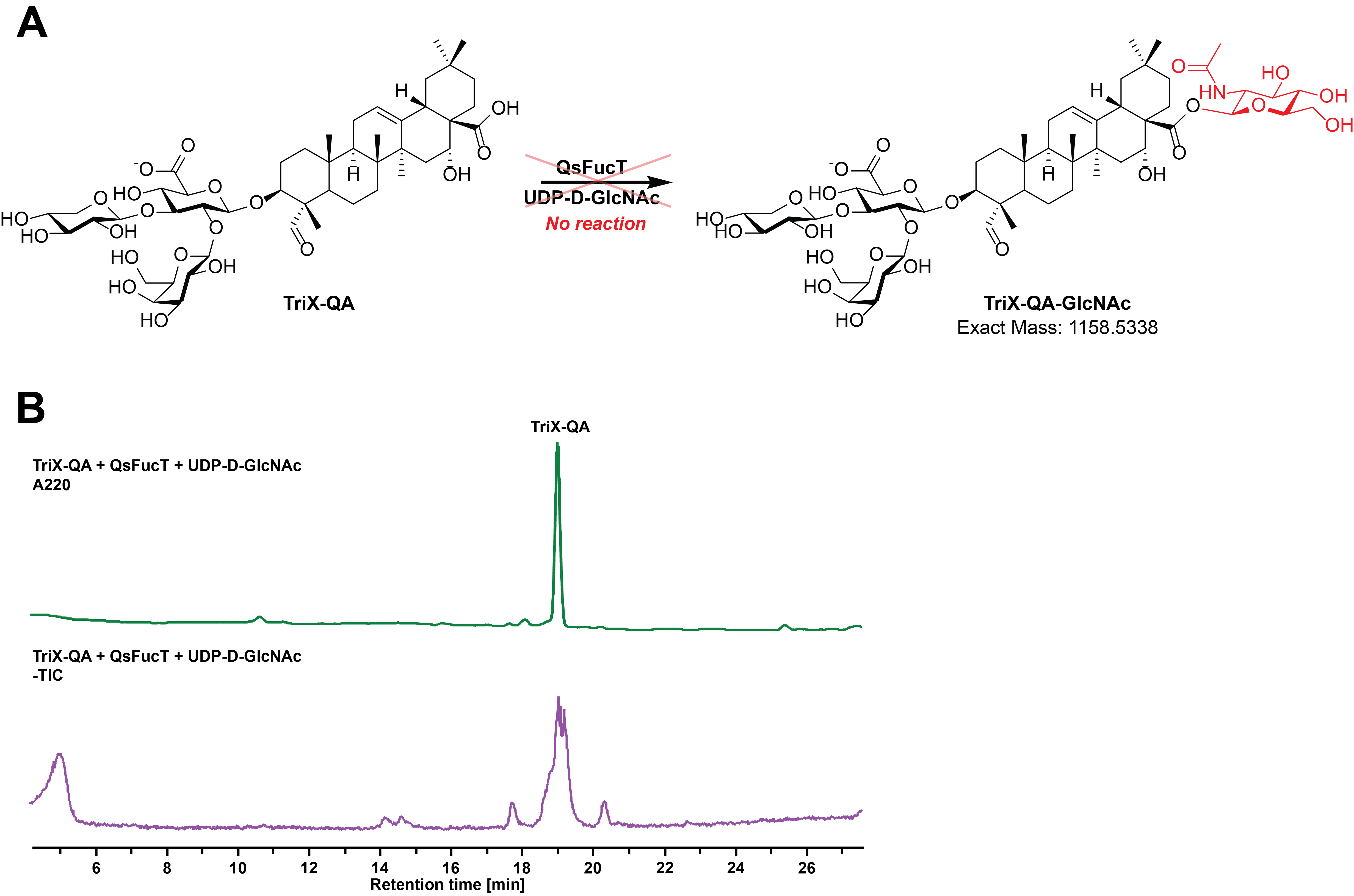
**

**Figure S22:** In vitro glycosylation of TriX-QA with UDP-D-GlcNAz by QsFucT.

(A) The reaction between TriX-QA and UDP-D-GlcNAz catalyzed by QsFucT. The starting material and product are depicted in the deprotonated form anticipated to be observed in negative mode ESI-MS. (B) A220 HPLC and negative mode TIC chromatograms of the glycosylation reaction. (C) ESI-MS of the product peak at Rt = 15.5 min.


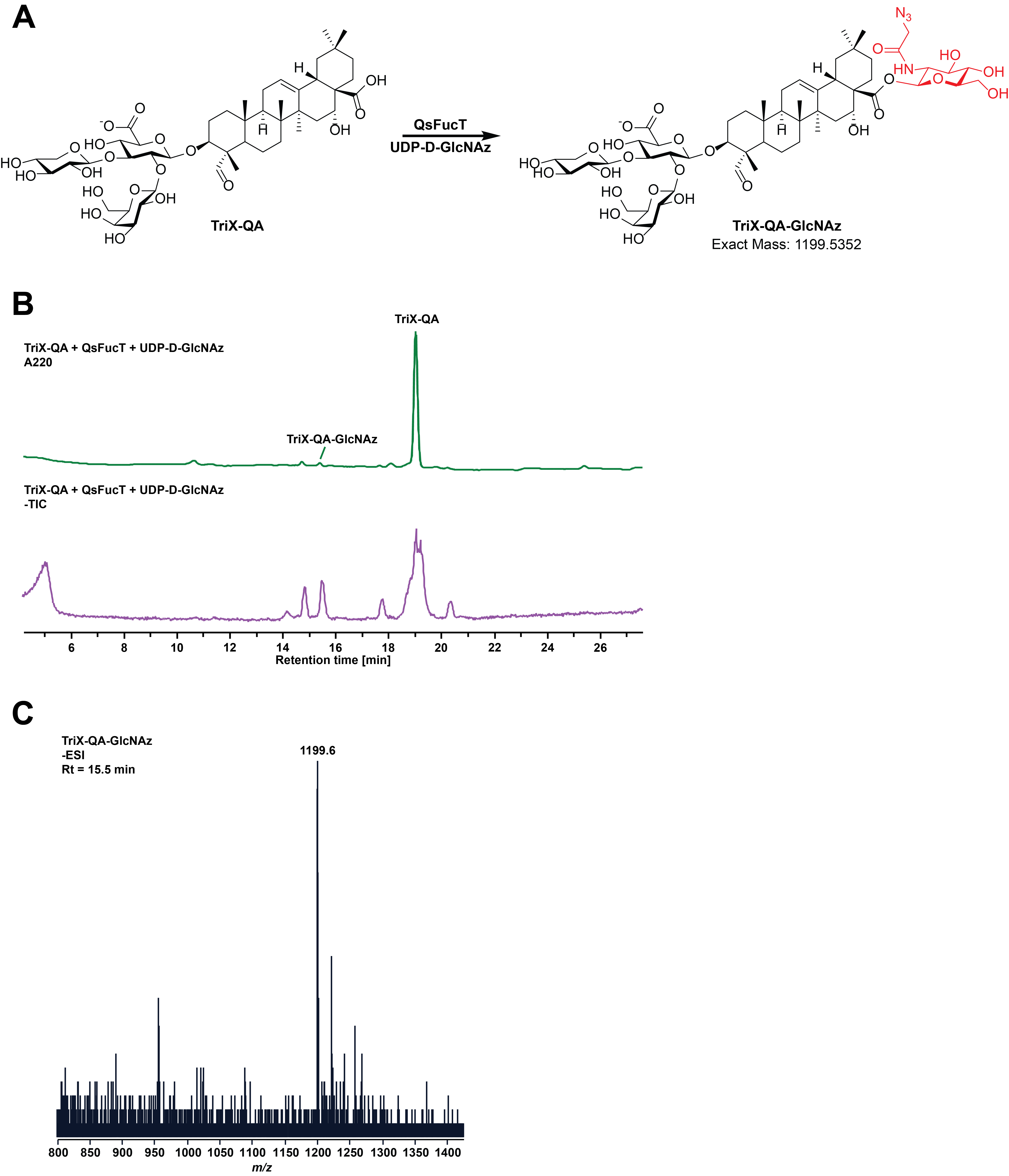


**Figure S23:** In vitro glycosylation of TriX-QA with UDP-6-azido-6-deoxy-D-Glc by QsFucT.

(A) The reaction between TriX-QA and UDP-6-azido-6-deoxy-D-Glc catalyzed by QsFucT. The starting material and product are depicted in the deprotonated form anticipated to be observed in negative mode ESI-MS. (B) A220 HPLC and negative mode TIC chromatograms of the glycosylation reaction. Note that the product peak partially overlaps with the starting material. (C) ESI-MS of the product peak at Rt = 19.4 min.

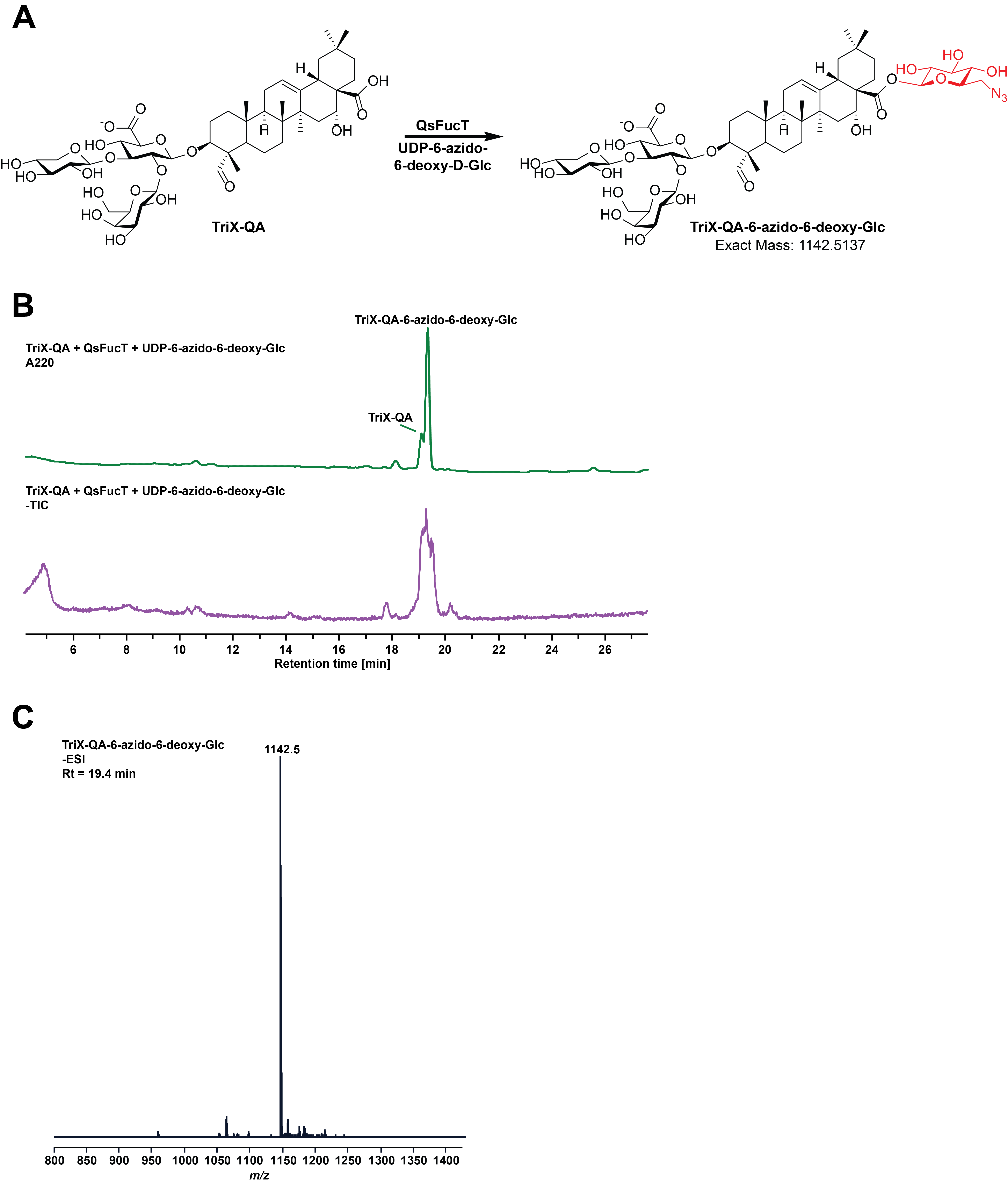


**Figure S24:** In vitro glycosylation of TriX-QA with UDP-D-Glc by SvFucT.

(A) The reaction between TriX-QA and UDP-D-Glc catalyzed by SvFucT. The starting material and product are depicted in the deprotonated form anticipated to be observed in negative mode ESI-MS. (B) A220 HPLC and negative mode TIC chromatograms of the glycosylation reaction. (C) ESI-MS of the product peak at Rt = 15.0 min.


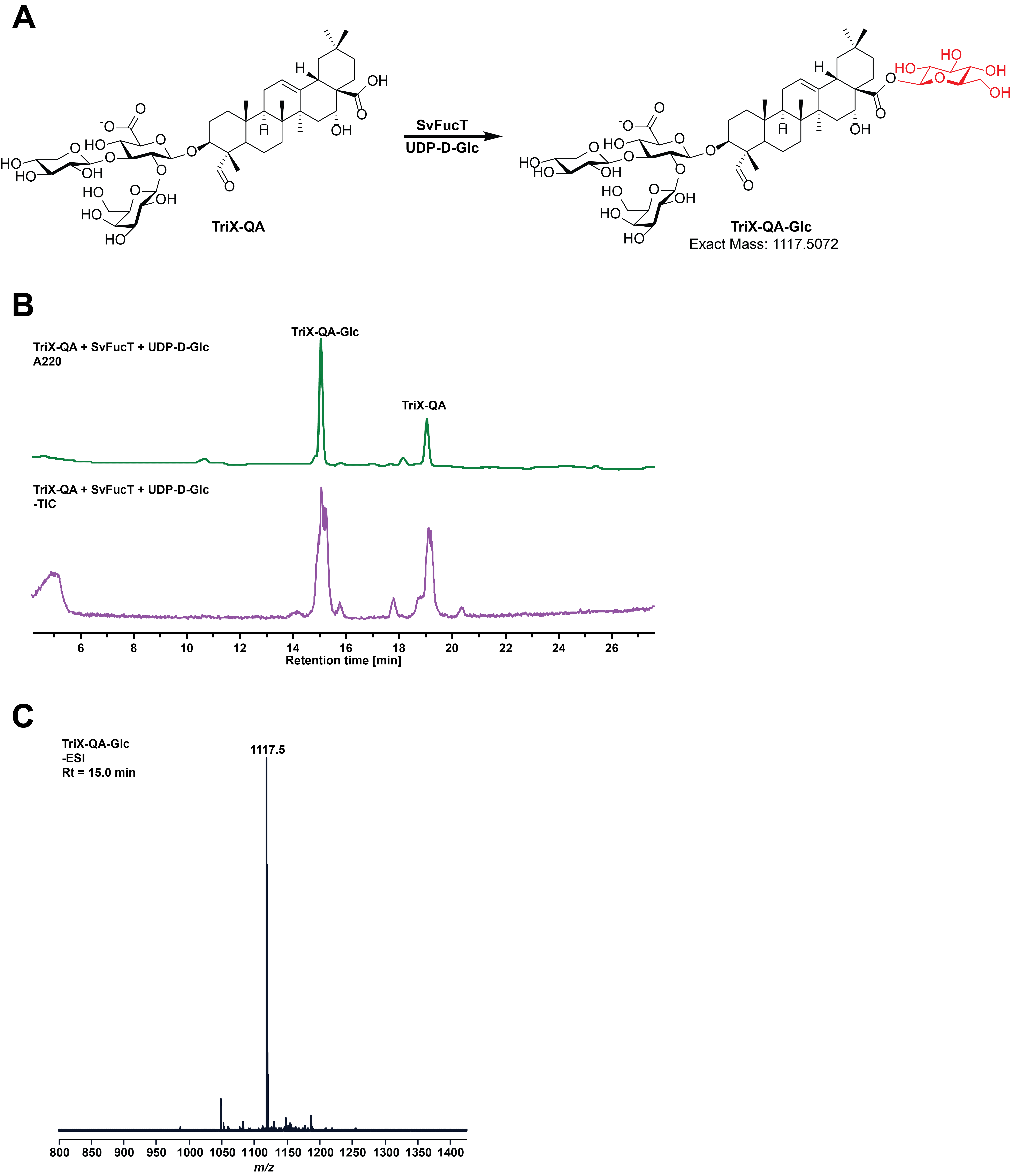


**Figure S25:** In vitro glycosylation of TriX-QA with UDP-D-Gal by SvFucT.

(A) The reaction between TriX-QA and UDP-D-Gal catalyzed by SvFucT. The starting material and product are depicted in the deprotonated form anticipated to be observed in negative mode ESI-MS. (B) A220 HPLC and negative mode TIC chromatograms of the glycosylation reaction. (C) ESI-MS of the product peak at Rt = 14.8 min

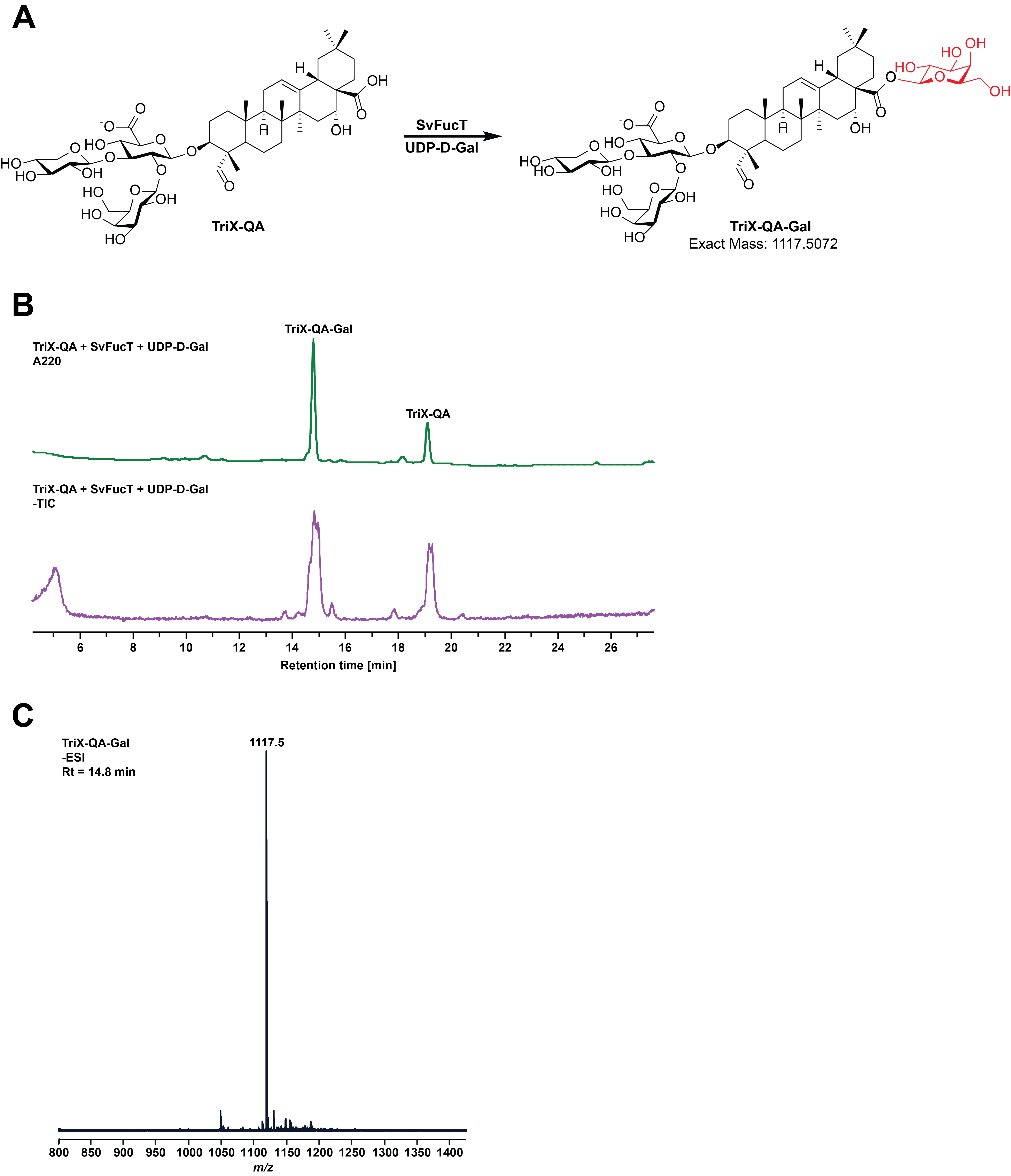


**Figure S26:** In vitro glycosylation of TriX-QA with UDP-L-Rha by SvFucT.

(A) The reaction between TriX-QA and UDP-L-Rha catalyzed by SvFucT. The starting material and product are depicted in the deprotonated form anticipated to be observed in negative mode ESI-MS. (B) A220 HPLC and negative mode TIC chromatograms of the glycosylation reaction. (C) ESI-MS of the product peak at Rt = 16.1 min.

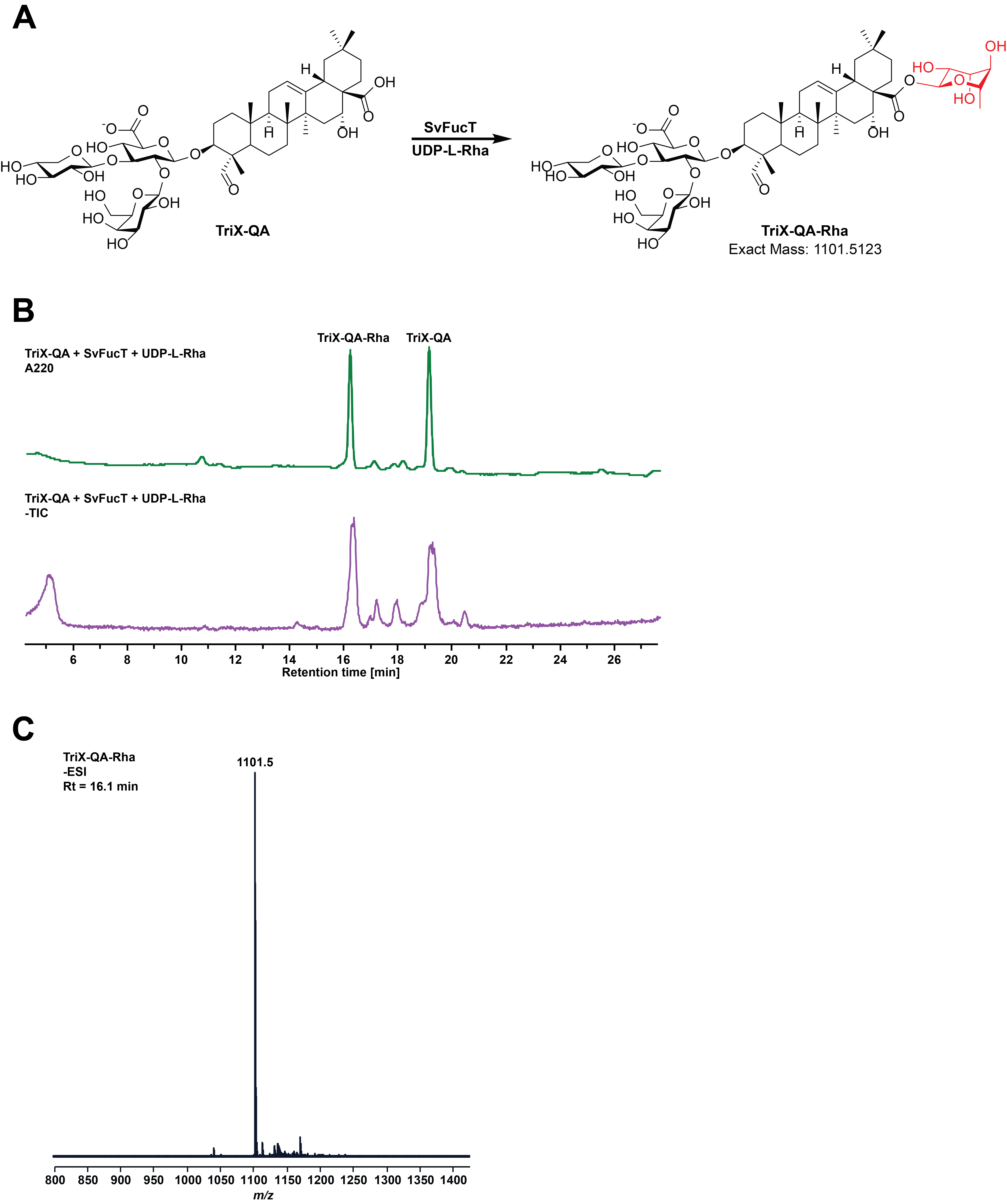


**Figure S27:** In vitro glycosylation of TriX-QA with UDP-D-Xyl by SvFucT.

(A) The reaction between TriX-QA and UDP-D-Xyl catalyzed by SvFucT. The starting material and product are depicted in the deprotonated form anticipated to be observed in negative mode ESI-MS. (B) A220 HPLC and negative mode TIC chromatograms of the glycosylation reaction. (C) ESI-MS of the product peak at Rt = 17.0 min.

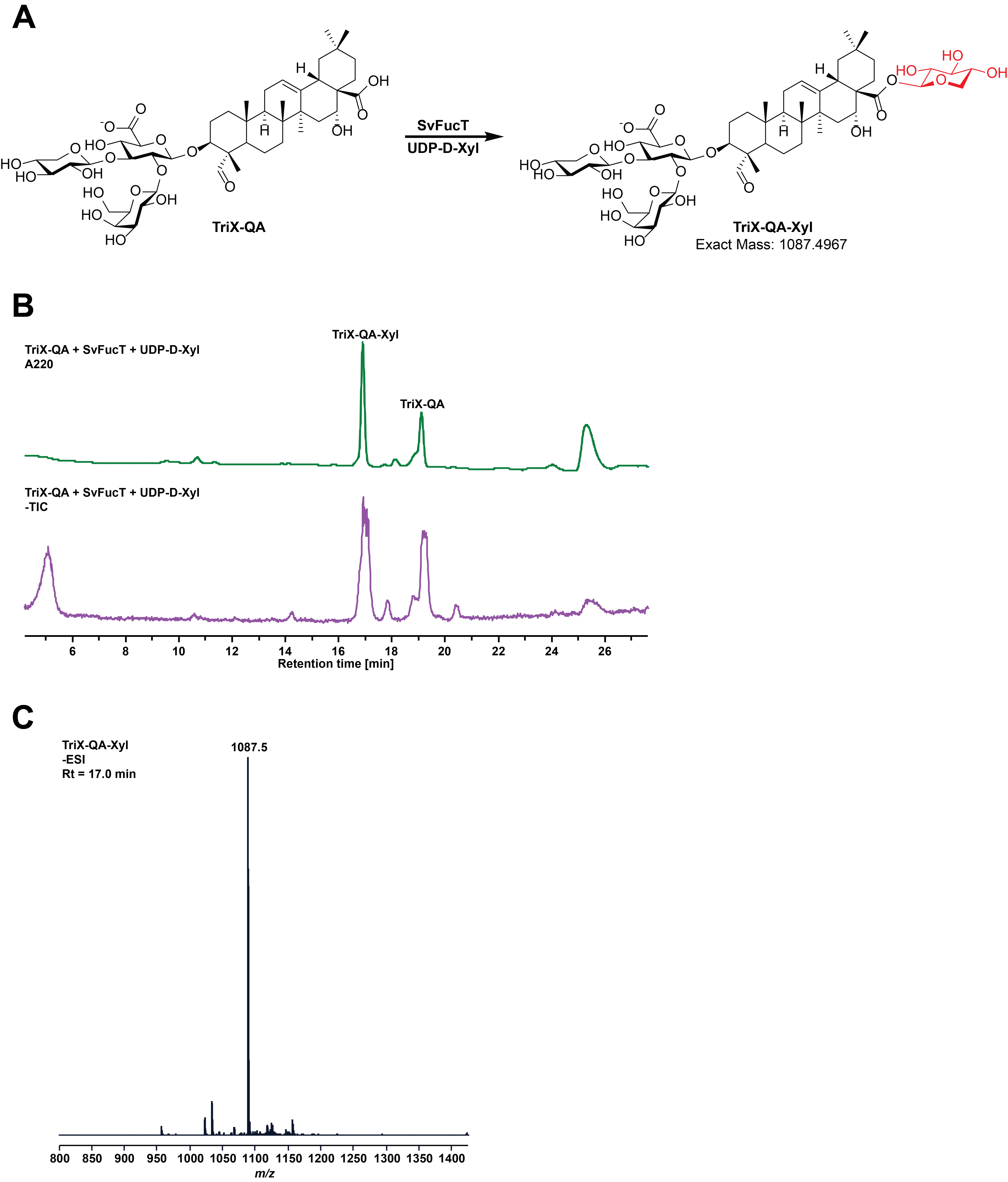


**Figure S28:** In vitro glycosylation of TriX-QA with UDP-L-Ara*p* by SvFucT.

(A) The reaction between TriX-QA and UDP-L-Ara*p* catalyzed by SvFucT. The starting material and product are depicted in the deprotonated form anticipated to be observed in negative mode ESI-MS. (B) A220 HPLC and negative mode TIC chromatograms of the glycosylation reaction. (C) ESI-MS of the product peak at Rt = 16.5 min.


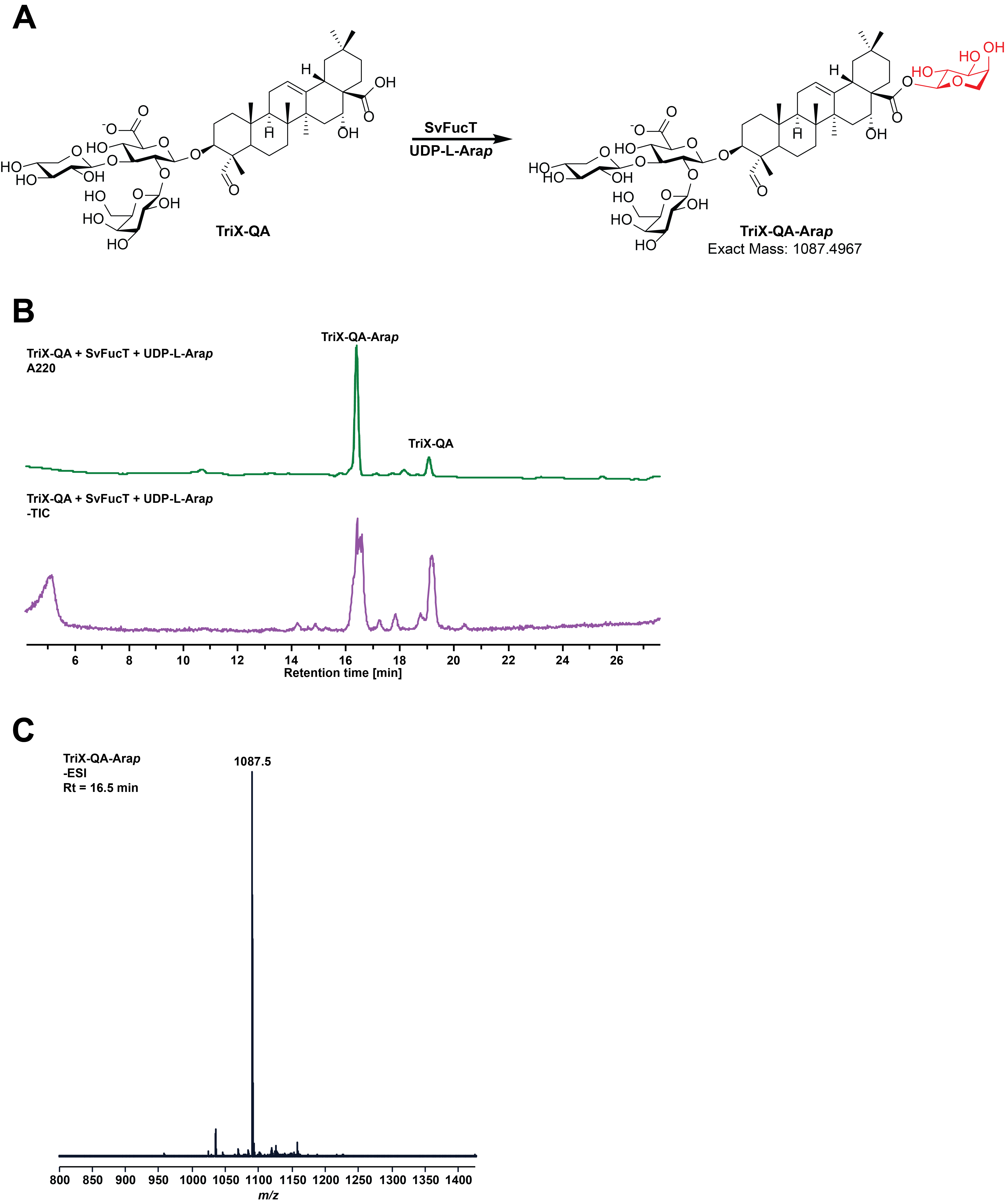


**Figure S29:** In vitro glycosylation of TriX-QA with UDP-L-Ara*f* by SvFucT.

(A) The reaction between TriX-QA and UDP-L-Ara*f* catalyzed by SvFucT. The starting material and product are depicted in the deprotonated form anticipated to be observed in negative mode ESI-MS. (B) A220 HPLC and negative mode TIC chromatograms of the glycosylation reaction. (C) ESI-MS of the product peak at Rt = 16.1 min.


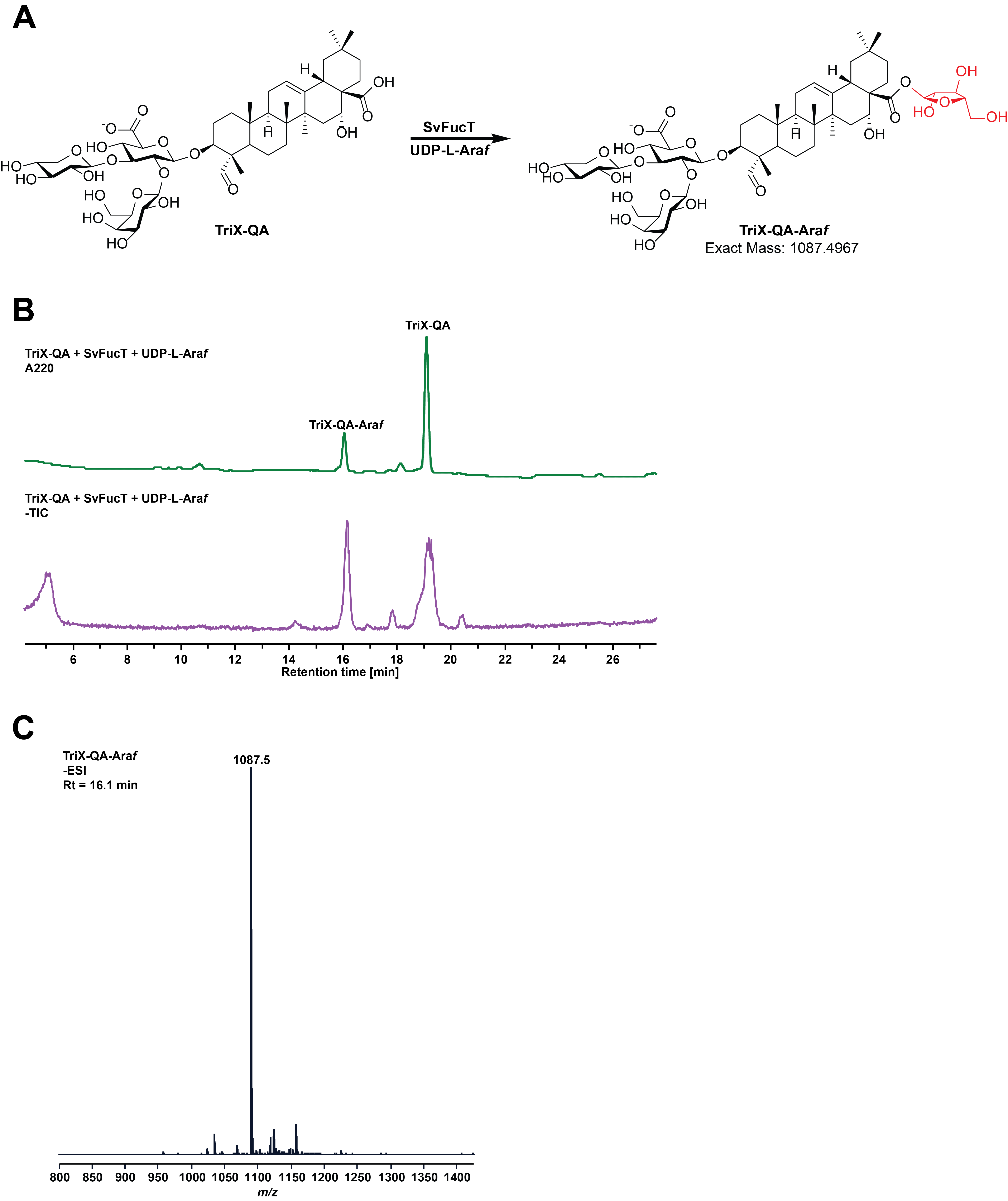


**Figure S30:** In vitro glycosylation of TriX-QA with UDP-D-GlcA by SvFucT.

(A) The non-reaction between TriX-QA and UDP-D-GlcA catalyzed by SvFucT. The starting material and product are depicted in the deprotonated form anticipated to be observed in negative mode ESI-MS. (B) A220 HPLC and negative mode TIC chromatograms of the glycosylation reaction. No significant products were observed.


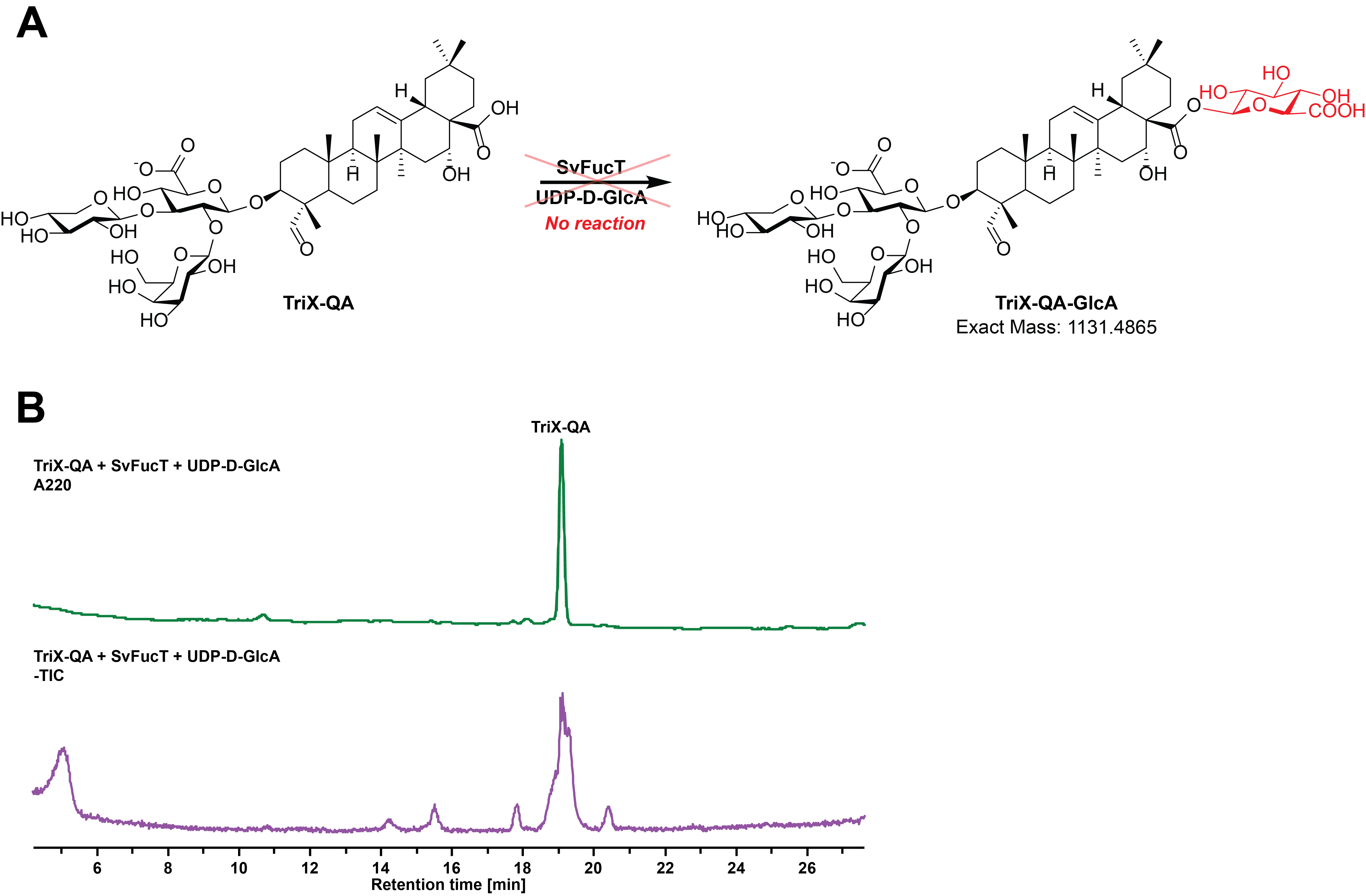


**Figure S31:** In vitro glycosylation of TriX-QA with UDP-D-GalA by SvFucT.

(A) The non-reaction between TriX-QA and UDP-D-GalA catalyzed by SvFucT. The starting material and product are depicted in the deprotonated form anticipated to be observed in negative mode ESI-MS. (B) A220 HPLC and negative mode TIC chromatograms of the glycosylation reaction. No significant products were observed.


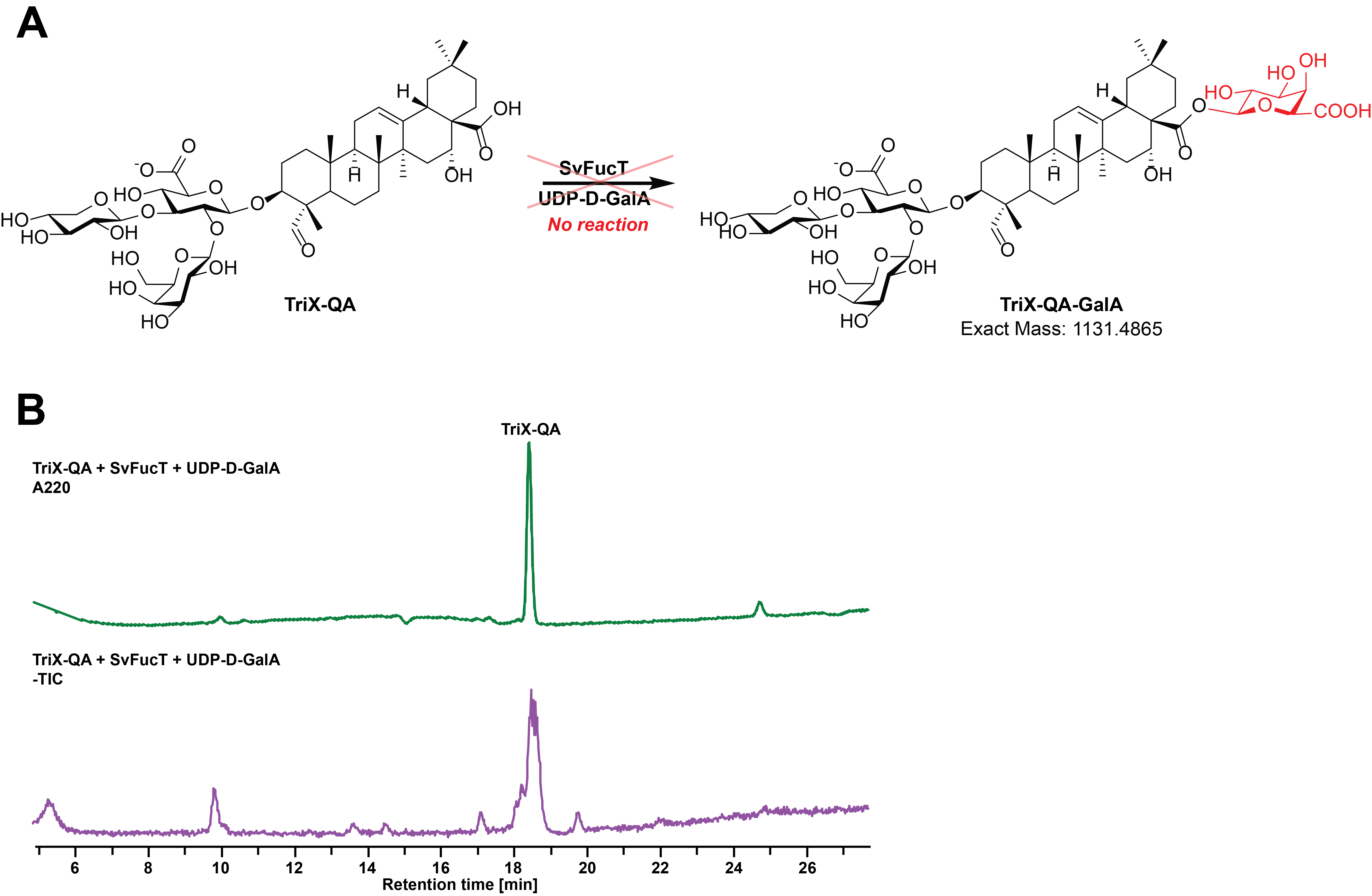


**Figure S32:** In vitro glycosylation of TriX-QA with UDP-D-GlcNAc by SvFucT.

(A) The reaction between TriX-QA and UDP-D-GlcNAc catalyzed by SvFucT. The starting material and product are depicted in the deprotonated form anticipated to be observed in negative mode ESI-MS. (B) A220 HPLC and negative mode TIC chromatograms of the glycosylation reaction. (C) ESI-MS of the product peak at Rt = 14.7 min.

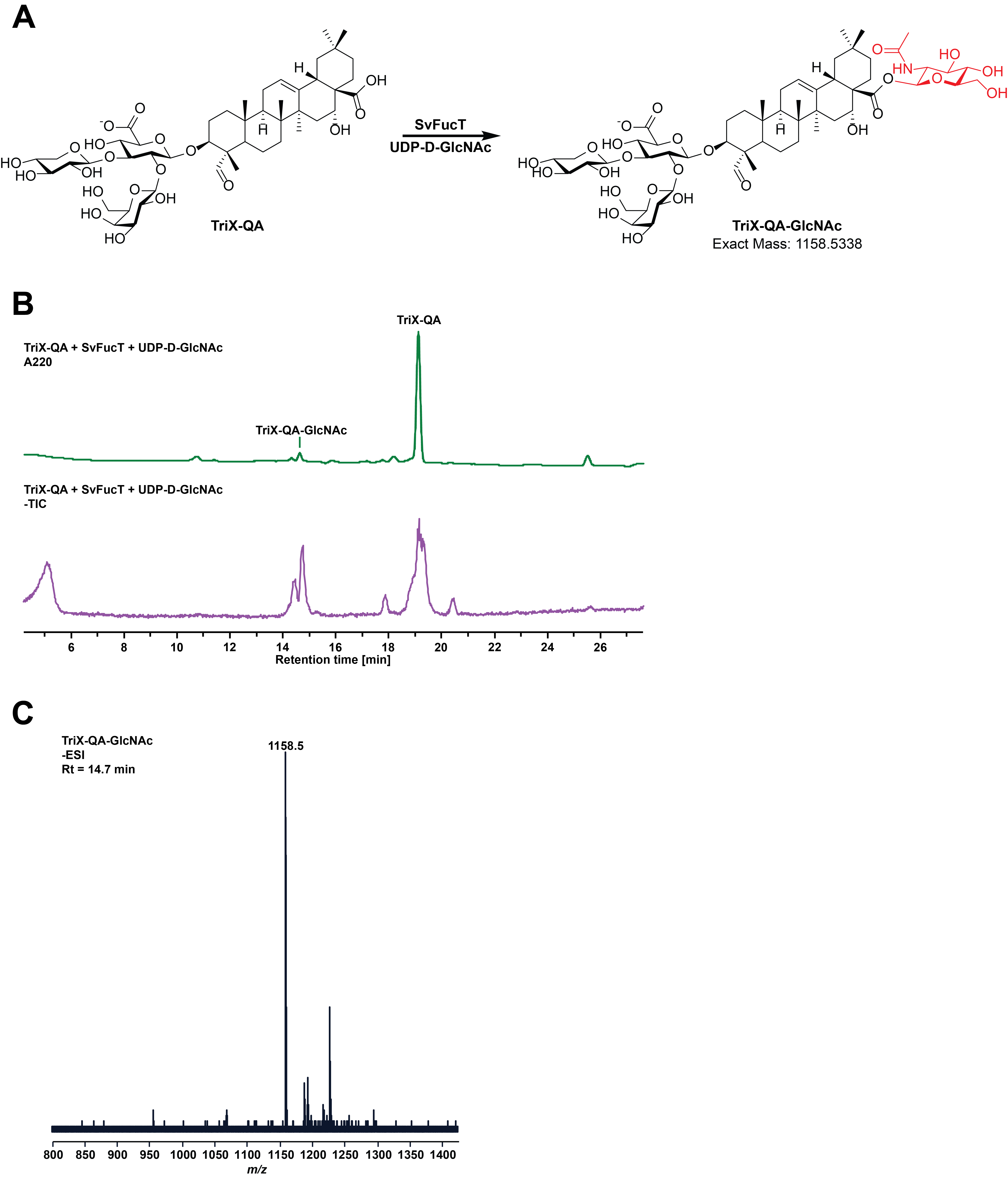


**Figure S33:** In vitro glycosylation of TriX-QA with UDP-D-GlcNAz by SvFucT.

(A) The reaction between TriX-QA and UDP-D-GlcNAz catalyzed by SvFucT. The starting material and product are depicted in the deprotonated form anticipated to be observed in negative mode ESI-MS. (B) A220 HPLC and negative mode TIC chromatograms of the glycosylation reaction. (C) ESI-MS of the product peak at Rt = 15.5 min.


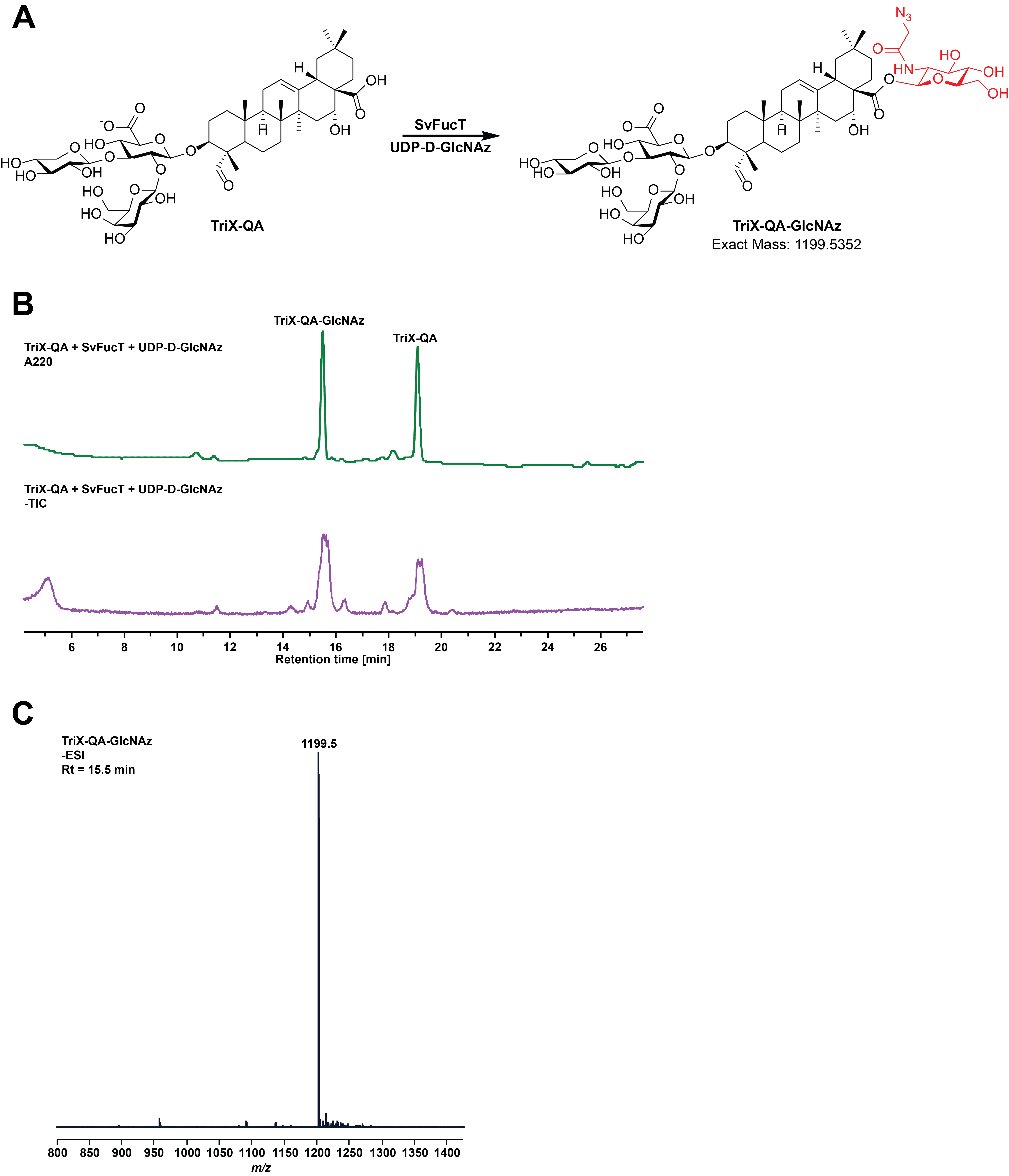


**Figure S34:** In vitro glycosylation of TriX-QA with UDP-6-azido-6-deoxy-D-Glc by SvFucT.

(A) The reaction between TriX-QA and UDP-6-azido-6-deoxy-D-Glc catalyzed by SvFucT. The starting material and product are depicted in the deprotonated form anticipated to be observed in negative mode ESI-MS. (B) A220 HPLC and negative mode TIC chromatograms of the glycosylation reaction. Note that the product peak partially overlaps with the starting material. (C) ESI-MS of the product peak at Rt = 19.4 min.

**
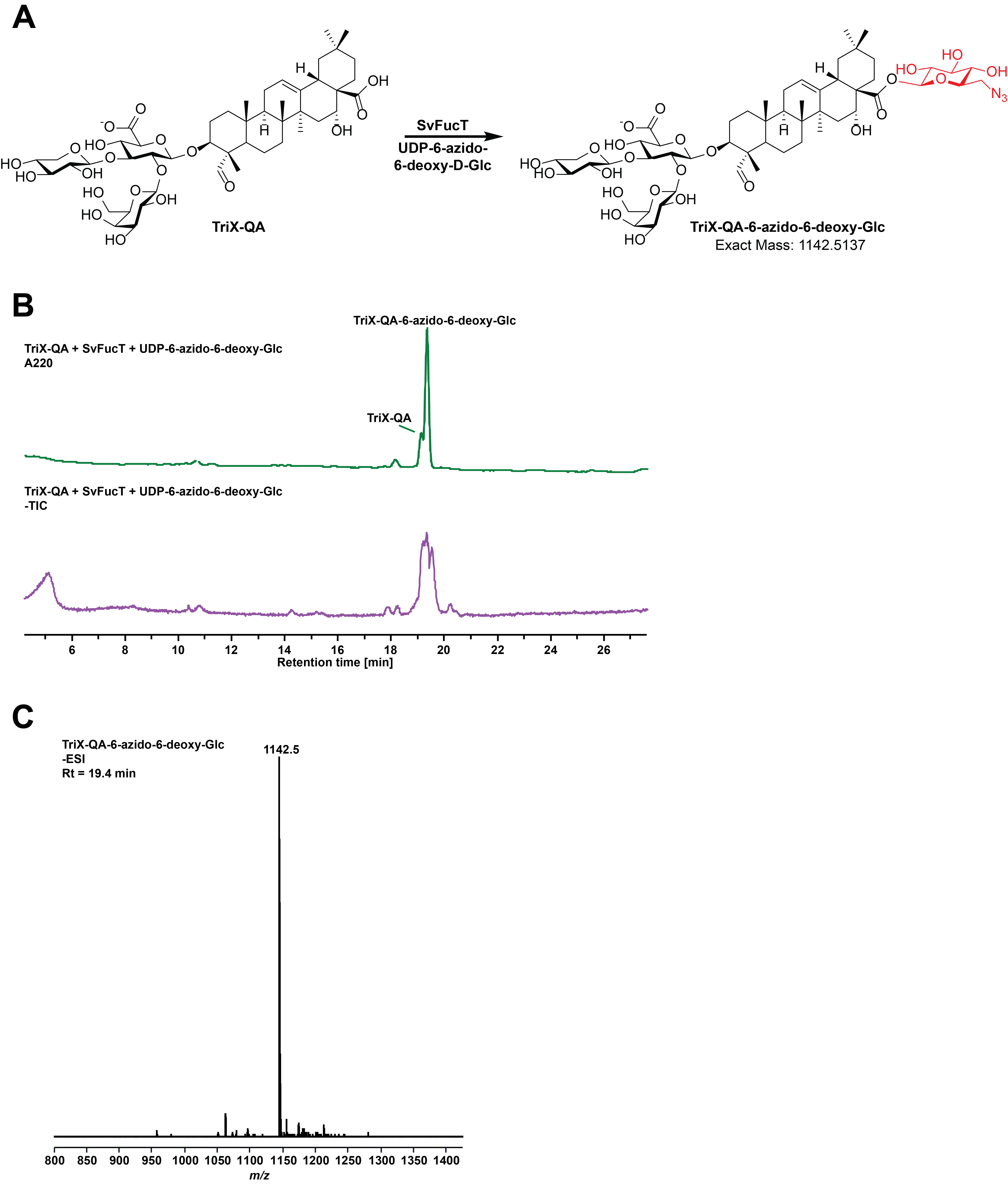
**

**Figure S35:** Kinetic assays of QsFucT with non-cognate UDP-sugar donors.

Shown are luminescence readings after a 10-minute glycosylation reaction at various reactant concentrations. Luminescence data were fit to the Michaelis-Menten model using OriginPro 2022b with the Orthogonal Distance Regression iteration algorithm. V_max_ in luminescence was converted to μmol UDP generated using a standard curve generated from UDP. Only donors that showed significant conversion in the endpoint glycosylation assays above were assayed. Curves were not fit if a reaction velocity plateau was not observed.


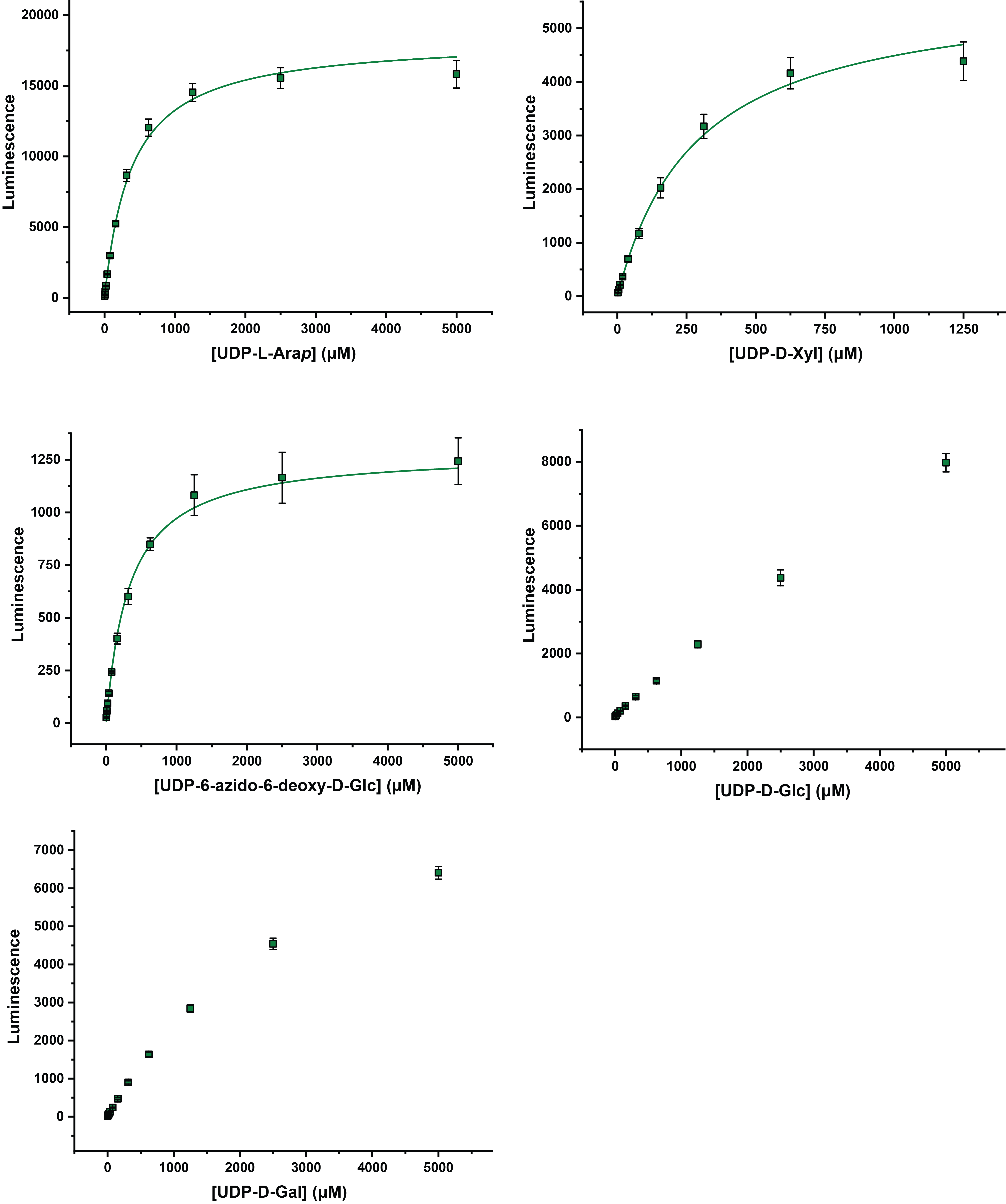


**Figure S36:** Kinetic assays of SvFucT with non-cognate UDP-sugar donors.

Shown are luminescence readings after a 10-minute glycosylation reaction at various reactant concentrations. Luminescence data were fit to the Michaelis-Menten model using OriginPro 2022b with the Orthogonal Distance Regression iteration algorithm. V_max_ in luminescence was converted to μmol UDP generated using a standard curve generated from UDP. Only donors that showed significant conversion in the endpoint glycosylation assays above were assayed. Curves were not fit if a reaction velocity plateau was not observed.


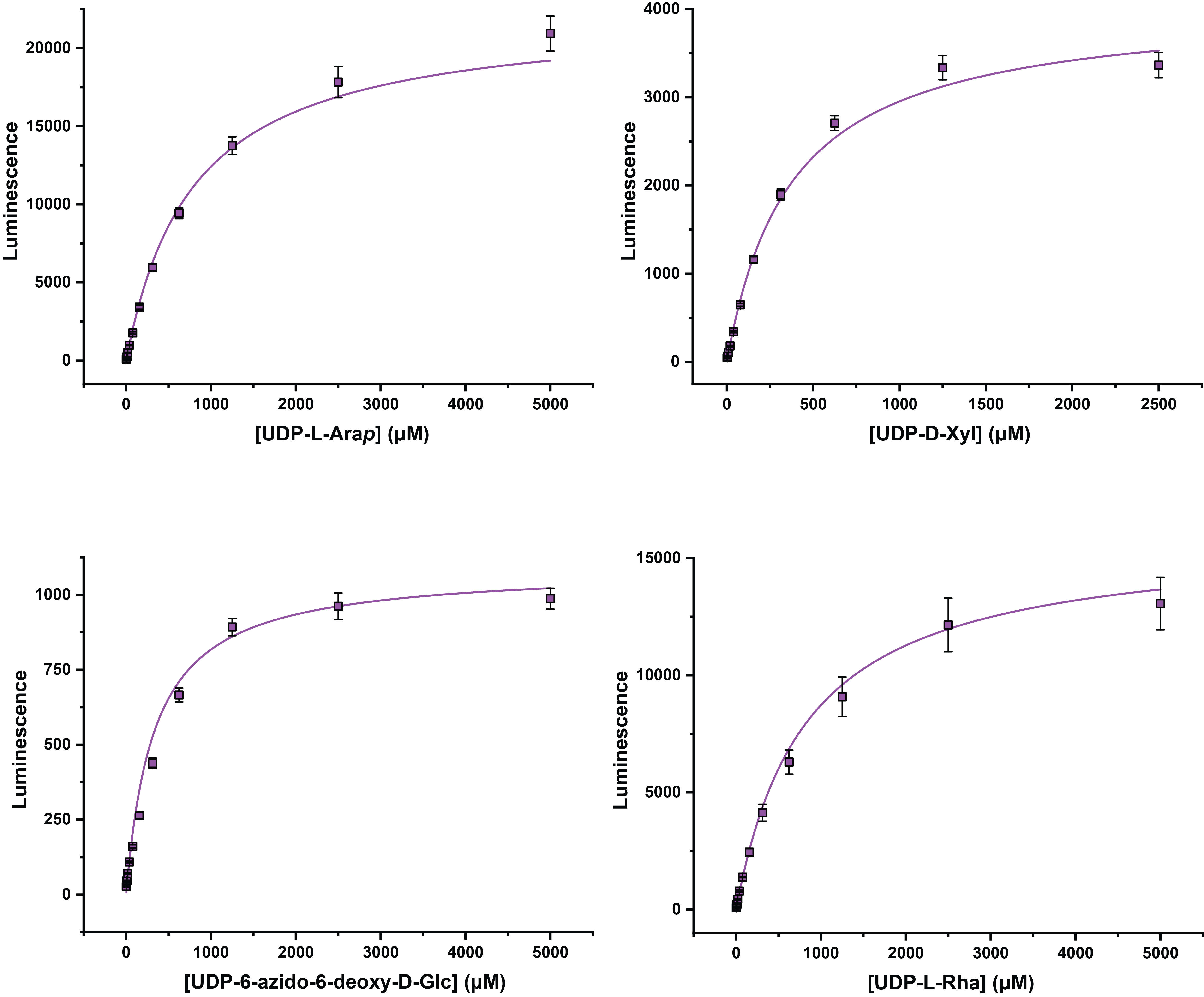


**Figure S36** (continued)


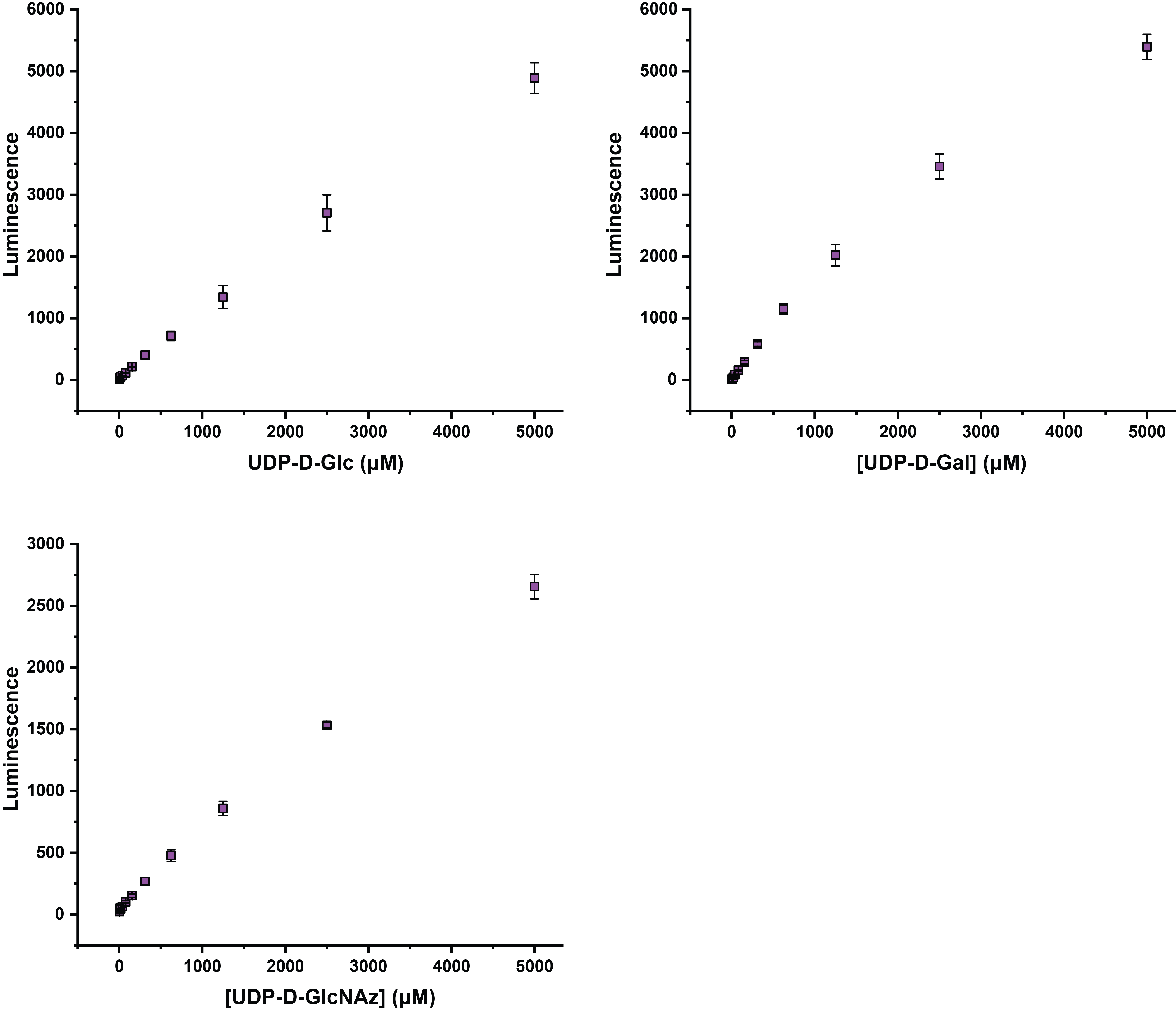


Figure S37: In vitro glycosylation of TriX-QA by QsFucT and SvFucT catalytic dyad substitution variants.

(A) In vitro glycosylation of TriX-QA using wild-type QsFucT, QsFucT (H21A) or QsFucT (D113N) at 20 μM. (B) In vitro glycosylation of TriX-QA using wild-type SvFucT, SvFucT (H20A), or SvFucT (D119N) at 20 μM. (C) In vitro glycosylation of TriX-QA using wild-type QsFucT, QsFucT (H21A) or QsFucT (D113N) at 200 nM (D) In vitro glycosylation of TriX-QA using wild-type SvFucT, SvFucT (H20A), or SvFucT (D119N) at 20 μM. All reactions also included UDP-4-keto-6-deoxy-D-Glc, QsFucSyn, and NADPH at standard assay concentrations noted in Experimental Methods.


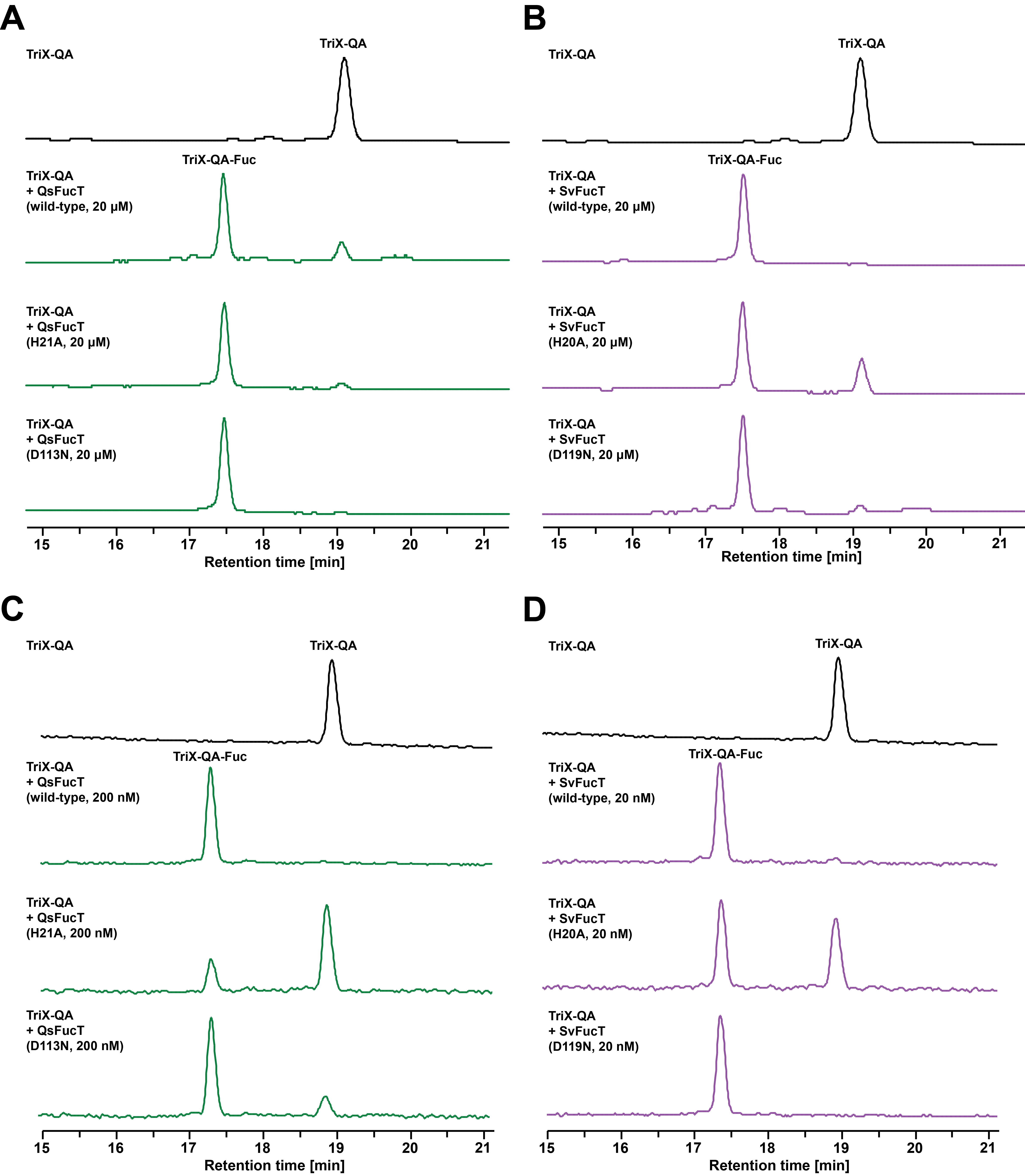


Table S4: Crystallographic statistics data for refinement of QsFucT and SvFucT

| Data collection | QsFucT - UDP | SvFucT - UDP | |
| --- | --- | --- | --- |
| Space group | P 21 | P 21 21 21 |  |
| Unit-Cell parameters (Å) | 55.237 76.552 58.975 90 115.398 90 | 50.564 110.73 170.548 90 90 90 |  |
| Resolution range (Å) | 43.73  - 2.11 (2.185  - 2.11) | 48.48  - 1.74 (1.802  - 1.74) |  |
| Total reflections | 94320 (7754) | 728444 (35030) |  |
| Unique reflections | 25419 (2539) | 91222 (7425) |  |
| R_merge_ (%) | 0.079 (1.16) | 0.106 (1.96) |  |
| *I*/σ*I* | 7.6 (0.8) | 11.8 (0.9) |  |
| Wilson B-factor | 39.83 | 21.8 |  |
| Completeness (%) | 99.14 (98.72) | 100 (99.6) |  |
| Redundancy | 3.8 (3.7) | 7.3 (7.3) |  |
| CC_1/2_ | 0.996 (0.320) | 0.999 (0.335) |  |
| *Refinement* |  |  |  |
| Resolution range (Å) | 43.73  - 2.11 (2.185  - 2.11) | 48.48 – 1.74 (1.8  - 1.74) |  |
| Reflections used in refinement | 25412 (2538) | 91218 (7425) |  |
| Reflections used for *R*_free_ | 1992 (199) | 1840 (153) |  |
| *R*_work_ | 0.2071 (0.3580) | 0.1749 (0.3133) |  |
| *R*_free_ | 0.2347 (0.3967) | 0.2100 (0.3592) |  |
| No. atoms |  |  |  |
| Proteins | 3417 | 7283 |  |
| Solvent | 107 | 846 |  |
| RMS from ideal geometry |  |  |  |
| Bond lengths (Å) | 0.003 | 0.012 |  |
| Bond angles (º) | 0.519 | 1.02 |  |
| Average B-factor | 54.98 | 31.27 |  |
| Macromolecules | 55.17 | 30.69 |  |
| Solvent | 50.10 | 36.61 |  |
| Ramachandran favored (%) | 96.93 | 96.78 |  |
| Ramachandran allowed (%) | 3.0 | 2.66 |  |
| Ramachandran outliers (%) | 0 | 0.55 |  |
| Clashscore | 5.91 | 6.30 |  |

Figure S38: Detailed depiction of the active sites of QsFucT and SvFucT.

UDP was bound in the solved crystallographic structures while the attached D-fucose sugar was added via docking.


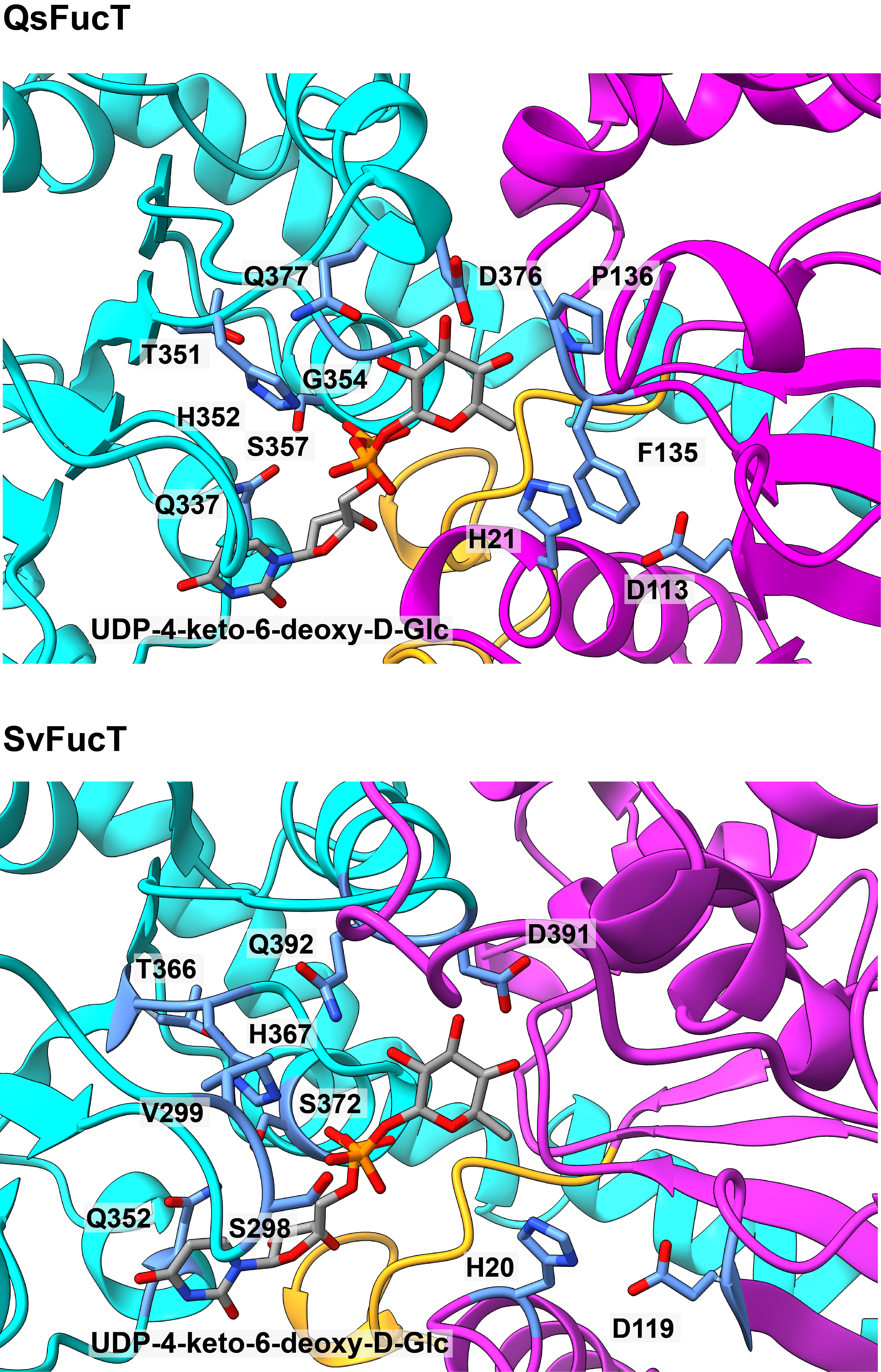


Figure S39: In vitro glycosylation of TriX-QA by additional active site variants.

Conversion percentages may be found on the next page in **Table S5**.

**
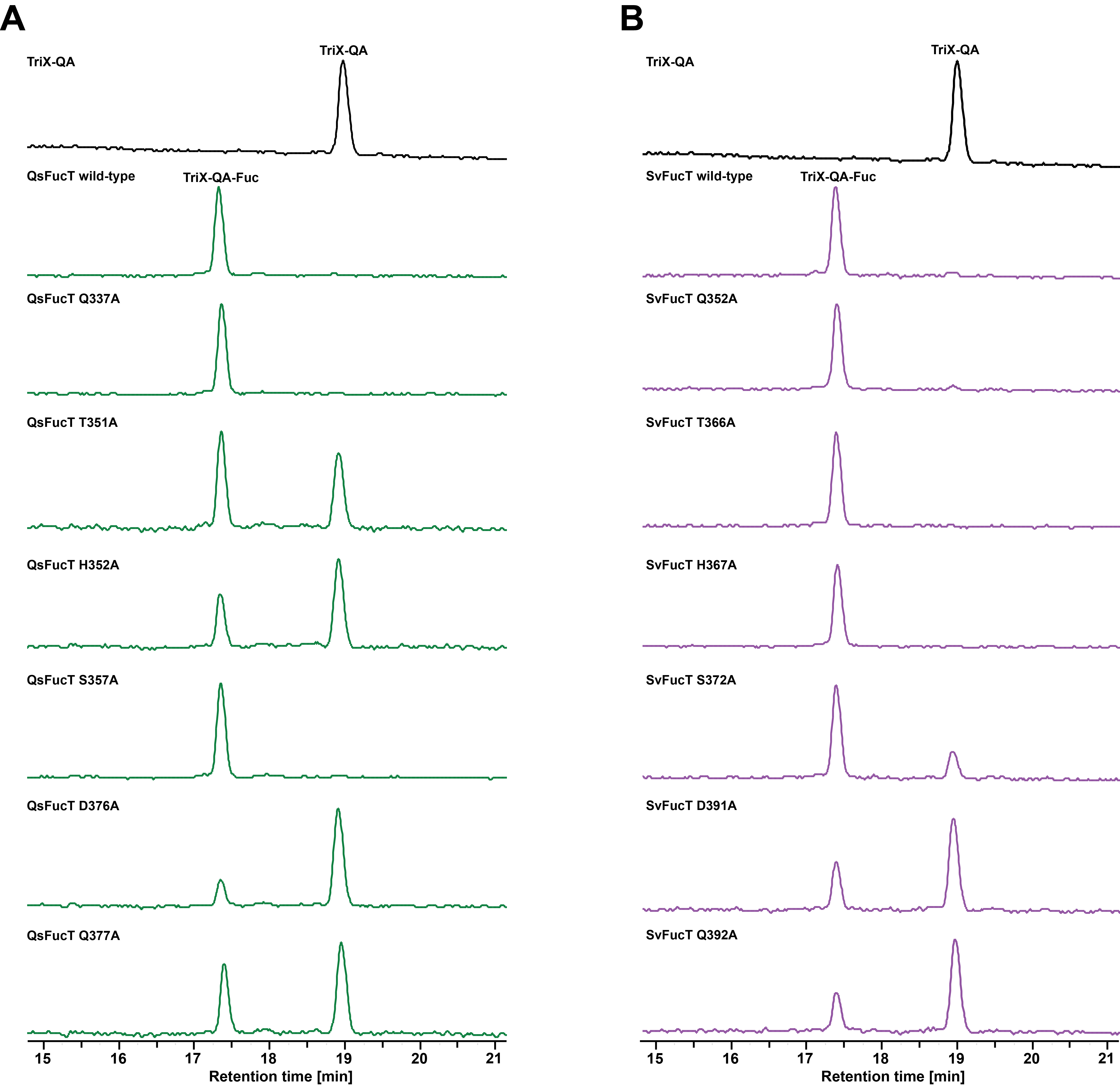
**

Table S5: Conversion data of tested QsFucT and SvFucT variants.

| **FucT variant** | **% conversion** |
| --- | --- |
| QsFucT Q337A | 100 |
| QsFucT T351A | 51.8 |
| QsFucT H352A | 36.3 |
| QsFucT S357A | 100 |
| QsFucT D376A | 42.1 |
| QsFucT Q377A | 19.8 |
| SvFucT Q352A | 95.6 |
| SvFucT T366A | 100 |
| SvFucT H367A | 100 |
| SvFucT S372A | 76.6 |
| SvFucT D391A | 31.1 |
| SvFucT Q392A | 25.7 |

Supporting Information References

(1) Wilkins, M. R.; Gasteiger, E.; Bairoch, A.; Sanchez, J.-C.; Williams, K. L.; Appel, R. D.; Hochstrasser, D. F. Protein Identification and Analysis Tools in the ExPASy Server. In *2-D Proteome Analysis Protocols*; Humana Press: New Jersey, 2003; Vol. 112, pp 531–552.

(2) Wilson, D. B.; Hogness, D. S. The Enzymes of the Galactose Operon in Escherichia Coli. I. Purification and Characterization of Uridine Diphosphogalactose 4-Epimerase. *J. Biol. Chem.* **1964**, *239*, 2469–2481.

(3) Reed, M. C.; Lieb, A.; Nijhout, H. F. The Biological Significance of Substrate Inhibition: A Mechanism with Diverse Functions. *Bioessays* **2010**, *32* (5), 422–429.

(4) Pereira, J. H.; McAndrew, R. P.; Tomaleri, G. P.; Adams, P. D. Berkeley Screen: A Set of 96 Solutions for General Macromolecular Crystallization. *J. Appl. Crystallogr.* **2017**, *50* (Pt 5), 1352–1358.

(5) Winter, G.; Lobley, C. M. C.; Prince, S. M. Decision Making in Xia2. *Acta Crystallogr. D Biol. Crystallogr.* **2013**, *69* (Pt 7), 1260–1273.

(6) McCoy, A. J.; Grosse-Kunstleve, R. W.; Adams, P. D.; Winn, M. D.; Storoni, L. C.; Read, R. J. Phaser Crystallographic Software. *J. Appl. Crystallogr.* **2007**, *40* (Pt 4), 658–674.

(7) Jumper, J.; Evans, R.; Pritzel, A.; Green, T.; Figurnov, M.; Ronneberger, O.; Tunyasuvunakool, K.; Bates, R.; Žídek, A.; Potapenko, A.; Bridgland, A.; Meyer, C.; Kohl, S. A. A.; Ballard, A. J.; Cowie, A.; Romera-Paredes, B.; Nikolov, S.; Jain, R.; Adler, J.; Back, T.; Petersen, S.; Reiman, D.; Clancy, E.; Zielinski, M.; Steinegger, M.; Pacholska, M.; Berghammer, T.; Bodenstein, S.; Silver, D.; Vinyals, O.; Senior, A. W.; Kavukcuoglu, K.; Kohli, P.; Hassabis, D. Highly Accurate Protein Structure Prediction with AlphaFold. *Nature* **2021**, *596* (7873), 583–589.

(8) Liebschner, D.; Afonine, P. V.; Baker, M. L.; Bunkóczi, G.; Chen, V. B.; Croll, T. I.; Hintze, B.; Hung, L. W.; Jain, S.; McCoy, A. J.; Moriarty, N. W.; Oeffner, R. D.; Poon, B. K.; Prisant, M. G.; Read, R. J.; Richardson, J. S.; Richardson, D. C.; Sammito, M. D.; Sobolev, O. V.; Stockwell, D. H.; Terwilliger, T. C.; Urzhumtsev, A. G.; Videau, L. L.; Williams, C. J.; Adams, P. D. Macromolecular Structure Determination Using X-Rays, Neutrons and Electrons: Recent Developments in Phenix. *Acta Crystallogr. D Struct. Biol.* **2019**, *75* (Pt 10), 861–877.

(9) Afonine, P. V.; Grosse-Kunstleve, R. W.; Echols, N.; Headd, J. J.; Moriarty, N. W.; Mustyakimov, M.; Terwilliger, T. C.; Urzhumtsev, A.; Zwart, P. H.; Adams, P. D. Towards Automated Crystallographic Structure Refinement with Phenix.Refine. *Acta Crystallogr. D Biol. Crystallogr.* **2012**, *68* (Pt 4), 352–367.

(10) Emsley, P.; Cowtan, K. Coot: Model-Building Tools for Molecular Graphics. *Acta Crystallogr. D Biol. Crystallogr.* **2004**, *60* (Pt 12 Pt 1), 2126–2132.

(11) Williams, C. J.; Headd, J. J.; Moriarty, N. W.; Prisant, M. G.; Videau, L. L.; Deis, L. N.; Verma, V.; Keedy, D. A.; Hintze, B. J.; Chen, V. B.; Jain, S.; Lewis, S. M.; Arendall, W. B., 3rd; Snoeyink, J.; Adams, P. D.; Lovell, S. C.; Richardson, J. S.; Richardson, D. C. MolProbity: More and Better Reference Data for Improved All-Atom Structure Validation. *Protein Sci.* **2018**, *27* (1), 293–315.

(12) Chen, X.; Hudson, G. A.; Mineo, C.; Amer, B.; Baidoo, E.; Crowe, S. A.; Liu, Y.; Keasling, J. D.; Scheller, H. V. Deciphering Triterpenoid Saponin Biosynthesis by Leveraging Transcriptome Response to Methyl Jasmonate Elicitation in Saponaria Vaccaria. *Nat. Commun.* **2023**, *14*, 7101.

(13) Madeira, F.; Madhusoodanan, N.; Lee, J.; Eusebi, A.; Niewielska, A.; Tivey, A. R. N.; Lopez, R.; Butcher, S. The EMBL-EBI Job Dispatcher Sequence Analysis Tools Framework in 2024. *Nucleic Acids Res.* **2024**, *52* (W1), W521–W525.
